# Decision-Rule Architecture in Fungal Biomarker-Guided Antifungal Stewardship for Critically Ill Adults: A Systematic Review and *Candida*-Focused Randomised Meta-Analysis

**DOI:** 10.3390/jof12090681

**Published:** 2026-09-10

**Authors:** Giuseppe Neri, Giuseppe Mazza, Jessica Ielapi, Alessandro Russo, Francesca Serapide, Helenia Mastrangelo, Aldo Mesiti, Zaninni Caroleo, Stefano Fresilli, Andrea Bruni, Simona Gigliotti, Angela Quirino, Giovanni Matera, Federico Longhini, Eugenio Garofalo

**Affiliations:** 1 Department of Medical and Surgical Sciences, Magna Graecia University, 88100 Catanzaro, Italy; 2 Department of Health Sciences, “Renato Dulbecco” University Hospital, 88100 Catanzaro, Italy; 3 Department of Pharmacy, Health and Nutritional Sciences, University of Calabria, 87036 Rende, Italy

**Keywords:** antifungal stewardship, fungal biomarkers, β-(1→3)-D-glucan, *Candida*, critical care, decision rule, meta-analysis

## Abstract

Fungal biomarkers may support antifungal stewardship in critically ill adults, but their clinical effect depends on the decision rule linking test results to treatment. We systematically reviewed ICU or ICU-relevant adult studies in which fungal biomarkers or rapid fungal diagnostics explicitly informed antifungal management. Searches were updated through 24 July 2026 (PROSPERO CRD420261432481), and clinically compatible *Candida* randomised outcomes were synthesised by rule direction. Twenty-one reports represented 19 independent studies. Five core *Candida* trials randomised 677 participants (661 analysed). Rule-in assignment increased systemic antifungal receipt (two trials; RR 2.06, 95% CI 1.58–2.67) without demonstrated patient benefit. In three rule-out trials, mortality was 34/126 versus 38/132 (RR 0.94, 95% CI 0.63–1.40), while only 16 post-randomisation invasive *Candida* events informed highly uncertain fungal-safety estimates (RR 1.85, 95% CI 0.51–6.65). The evidence is therefore insufficient to establish a clinically acceptable fungal-safety margin for biomarker-guided discontinuation. Biomarkers should therefore support, not replace, risk-based reassessment: timely negative results may justify supervised discontinuation, whereas isolated positivity should not be a stand-alone treatment trigger.

## 1. Introduction

Invasive candidiasis in critically ill adults creates a recurrent therapeutic asymmetry. Delayed active therapy can be consequential in patients with true disease, yet the low prevalence and nonspecific presentation of suspected infection expose many patients without invasive candidiasis to empirical antifungals. Blood cultures remain insensitive and slow, tissue confirmation is rarely feasible in unstable patients, and colonisation or clinical risk factors alone lack sufficient specificity. Contemporary global and US guidance therefore frames diagnosis as an integrated probability assessment combining host risk, clinical syndrome, microbiology, imaging, and non-culture diagnostics rather than as the output of a single assay [1,2,3,4].

Among non-culture tests, serum β-(1→3)-D-glucan (BDG) is the most extensively studied, while *Candida* mannan or anti-mannan assays and rapid molecular tests may provide complementary or earlier information [5,6]. Their clinical performance is conditional on disease prevalence, assay threshold, timing and repetition of sampling, fungal species, organ support, and non-infectious causes of positivity [5,7,8,9,10]. The 2025 global candidiasis guideline treats BDG as one component of a broader diagnostic assessment and does not support isolated BDG positivity as a stand-alone trigger for antifungal initiation; current IDSA guidance recognises that a negative non-culture assay with high negative predictive value can support stopping empirical therapy in selected nonresponding ICU patients; and European ICU consensus emphasises access to results within 48 h to inform reassessment and, when appropriate, cessation of empirical treatment [2,3,4].

These recommendations reveal a central distinction: diagnostic accuracy and stewardship utility are not interchangeable. A positive-result algorithm can expand treatment, whereas a negative or serially negative result can narrow it. The biomarker is therefore not the intervention; the intervention is the decision rule, timing, and clinical context that convert probability into action. In a low-prevalence setting, even a reasonably sensitive assay may perform poorly as a universal rule-in trigger, while negative evidence may be more useful during structured reassessment. The recent ICU literature likewise emphasises integrated, pathway-based interpretation rather than isolated test positivity [1].

The randomised *Candida* evidence has not consistently been organised around this therapeutic distinction. Five trials embedded biomarkers in different clinical tasks: pre-emptive initiation, discontinuation or interruption, and early termination [11,12,13,14,15]. They enrolled different populations, used different biomarker strategies and sampling schedules, and measured antifungal exposure through non-equivalent endpoints. Pooling these studies as a single construct of “biomarker-guided therapy” risks averaging interventions that move prescribing in opposite directions. Beyond *Candida*, critical-care evidence remains dominated by diagnostic or observational studies, which can inform plausibility and implementation but cannot establish causal treatment effects.

We therefore conducted a systematic review, *Candida*-focused randomised meta-analysis, and structured narrative synthesis of fungal biomarker-guided and biomarker-informed antifungal management in critically ill adults. We organised the evidence by study design, fungal target, biomarker, and algorithmic intent; estimated effects on short-term mortality, systemic antifungal exposure, and subsequent invasive *Candida* infection; and examined implementation signals without conflating diagnostic performance with treatment effect. The central question was not whether fungal biomarkers “work” in the abstract, but which biomarker–action–patient pathways alter prescribing without compromising clinically important outcomes.

## 2. Materials and Methods

### 2.1. Ethics

Ethics approval was not required because this systematic review used only published aggregate data and involved no direct participant contact or access to identifiable information.

### 2.2. Review Design, Registration and Reporting

This systematic review, randomised trial meta-analysis, and structured narrative synthesis were conducted according to PRISMA 2020 and PRISMA-S and prospectively registered in PROSPERO (CRD420261432481) on 25 June 2026 [16,17]. The registered record prespecified separate interpretation of initiation/pre-emptive and exposure-reduction strategies. The review evaluated fungal biomarker-guided or biomarker-informed antifungal management in critically ill adults and organised the evidence by study design, fungal target, biomarker, and algorithmic intent. Extended operational methods and the complete statistical analysis plan are provided in the Electronic Supplemental Materials (ESM), together with Supplementary Tables S1–S8 covering search strategies and PRISMA reconciliation (Table S1), data-extraction and narrative/diagnostic outcome frameworks (Table S2), statistical rules and sensitivity analyses (Table S3), report-level exclusions (Table S4), included-study characteristics and synthesis classification (Table S5), risk-of-bias assessments (Table S6), GRADE certainty assessments (Table S7), and randomised event counts, study-level effects, and sensitivity analyses (Table S8).

### 2.3. Eligibility Criteria and PICO Framework

Eligible populations were adults admitted to an ICU or managed in an ICU-relevant setting with suspected or proven invasive fungal disease. Eligible interventions used a fungal biomarker or rapid fungal diagnostic result to inform a prespecified or observed antifungal action. Tests included BDG, *Candida* mannan or anti-mannan, T2Candida/T2MR, galactomannan, pathogen-specific or panfungal PCR, cryptococcal antigen, and metagenomic next-generation sequencing. Comparators included usual, culture-based, clinician-directed, empirical, pre-emptive, historical, contemporaneous, or alternative diagnostic-management pathways. Outcomes included mortality, systemic antifungal exposure, initiation, withholding, discontinuation or de-escalation, proven or probable invasive fungal infection, candidemia, breakthrough infection, restart or rescue therapy, adverse events, diagnostic performance, implementation, and resource use.

Rule-in strategies were defined as result-contingent increases in treatment initiation or acceleration; rule-out strategies were defined as result-contingent reductions in continuation of already-started therapy. Randomised exposure-reduction trials were analysed separately from initiation/pre-emptive trials because the policies addressed different decisions and could alter treatment in opposite directions. Non-randomised management and pathway studies were eligible for structured narrative synthesis. Blinded diagnostic or decision-analytic reports were retained only when they addressed an explicit treatment-relevant rule and were used to inform test behaviour or pathway plausibility, not treatment effect or safety. Diagnostic-only studies without an explicit antifungal decision were excluded. Linked reports were retained for unique information without double-counting study populations.

### 2.4. Search, Selection, and Data Extraction

MEDLINE/PubMed and Scopus were searched from inception on 25 June 2026 and updated through 24 July 2026. CENTRAL was searched on 28 June and updated on 24 July 2026; ClinicalTrials.gov and WHO ICTRP were searched on 29 June 2026. Citation searching, known-trial checks, and PROSPERO overlap checks were completed before final synthesis. No language or date restrictions were applied to the original searches; date/year limits were used only to operationalise the update where supported by the interface.

Two reviewers independently screened the records and full texts, with disagreements resolved by discussion and, when necessary, senior adjudication. Full strategies, source accounting, deduplication, and update reconciliation are reported in Table S1 (in the ESM); report-level exclusions are listed in Table S4 (in the ESM).

Two reviewers independently extracted and cross-checked the study design, population, fungal target, assay and threshold, sampling and turnaround, algorithmic intent, antifungal action, comparator, follow-up, implementation, and outcome data. Randomised extraction included arm denominators, event counts, continuous outcomes, and time points; non-randomised and diagnostic-management extraction included antifungal exposure, safety, diagnostic performance, adherence, override, and resource-use measures. Unreported values were not imputed. The primary clinical outcome was 28- or 30-day all-cause mortality; stewardship outcomes captured systemic antifungal receipt, timing, duration, discontinuation, antifungal-free days, and consumption. The pooled fungal-safety endpoint included only invasive *Candida* events first diagnosed after randomisation or explicitly reported as subsequent or follow-up events. The complete extraction framework is in Table S2 (in the ESM).

### 2.5. Risk of Bias and Certainty

Risk of bias was assessed independently using RoB 2 for randomised results [18], ROBINS-I for comparative non-randomised intervention estimates [19], and QUADAS-3 version 1.2 for diagnostic accuracy estimates [20]. Reports without a defensible causal comparator received a structured design-limitations appraisal. GRADE was applied to decision-specific randomised outcomes [21,22], with no unitary certainty rating assigned to the descriptive mortality aggregation across opposing rule directions. Detailed risk-of-bias and GRADE judgements are reported in Tables S6 and S7 (in the ESM).

### 2.6. Statistical Analysis

For dichotomous outcomes, crude risk ratios (RRs) with 95% confidence intervals (CIs) were calculated. Meta-analysis required at least two clinically compatible randomised comparisons and used the independent study population as the unit of analysis. Primary quantitative synthesis was restricted to core *Candida* biomarker-guided RCTs and stratified by rule direction; the mixed-fungal acute-on-chronic liver failure (ACLF) RCT was analysed separately. Random-effects synthesis was prespecified. DerSimonian–Laird inverse-variance analysis was selected before pooling as the primary calculation [23], with a 0.5 continuity correction when one arm contained zero events. Heterogeneity was described using Q, τ^2^, and I^2^ and interpreted cautiously because few studies contributed to each pool. Because continuity corrections can materially alter sparse-event estimates and may attenuate them toward the null in some configurations, uncorrected Mantel–Haenszel analyses and, for fungal safety, uncorrected risk-difference analyses were used as sensitivity analyses.

Robustness analyses included REML/Wald, modified Hartung–Knapp, Mantel–Haenszel [24,25,26], and, for fungal safety, risk-difference models. Alternative estimators, leave-one-out analyses, the proven-only Rouzé analysis, missing-outcome scenarios, and the low-activation Erb exclusion, were post hoc. Prespecified decision-specific analyses included fixed-time mortality, exposure-reduction mortality, and post-randomisation invasive *Candida* events under rule-out assignment; the pooled rule-in systemic-antifungal-receipt analysis was exploratory. Clinically non-equivalent continuous exposure endpoints were not pooled. A descriptive mortality aggregation across opposing rule directions was retained only for context and was not interpreted as a unitary intervention effect. Full endpoint definitions, formulae, pooling rules, sensitivity analyses, and narrative-only criteria are provided in Table S3; study-level counts and quantitative results are presented in Table S8 (in the ESM). Meta-analyses and forest plots were performed using Review Manager (RevMan), version 5.4.1 (The Cochrane Collaboration). Additional statistical and sensitivity analyses not implemented in RevMan were performed using Python 3.13.5, NumPy 2.3.5, and SciPy 1.17.0. Funnel-plot or small-study-effect assessment was not undertaken because no synthesis included ten studies.

### 2.7. Structured Narrative Synthesis

Narrative synthesis followed SWiM principles [27] and grouped studies a priori by design, fungal target, algorithmic intent, and outcome domain. When pooling was inappropriate, the synthesis compared rule direction, trigger logic, threshold, sampling and reporting time, baseline risk, algorithm activation, adherence or override, and rescue provisions. Non-randomised evidence was restricted to feasibility, implementation, post-test probability, and hypothesis-generating pathway interpretation.

## 3. Results

**Figure 1:**
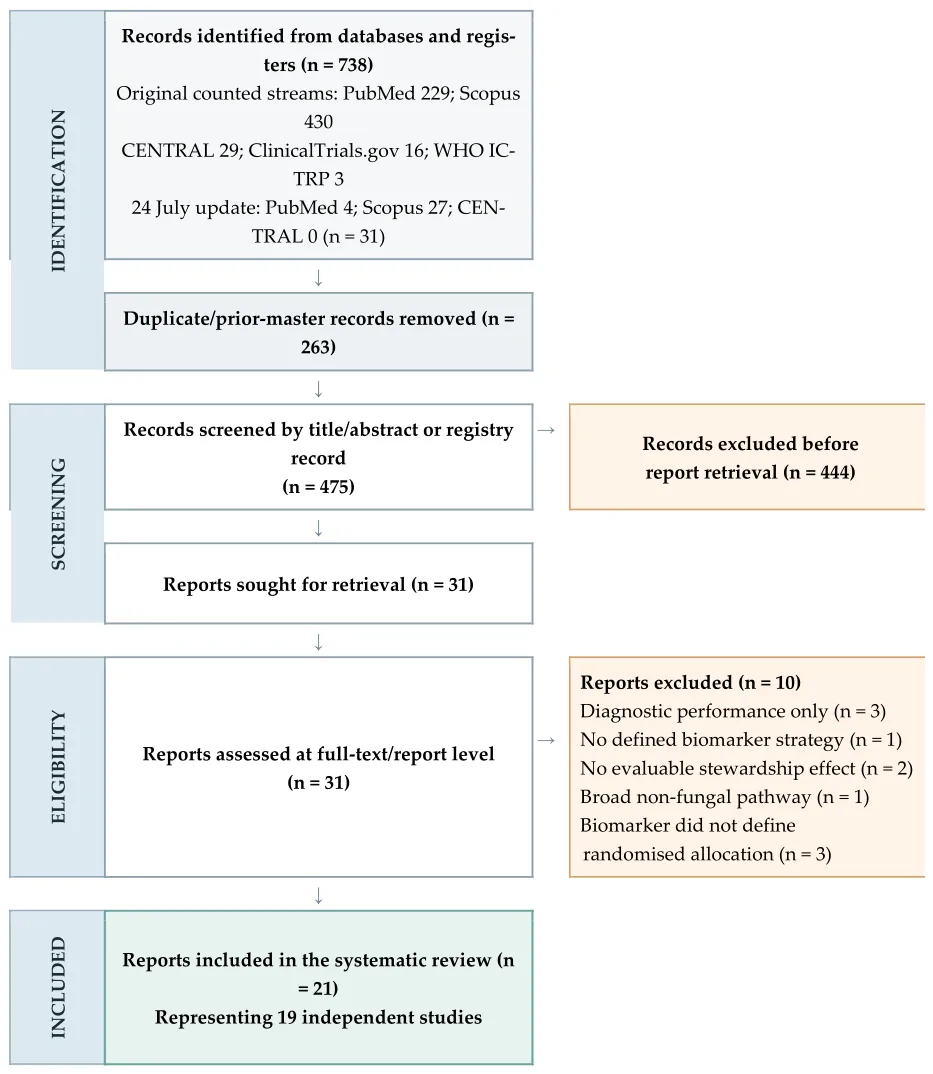
PRISMA 2020 flow diagram. PRISMA 2020 flow diagram showing 738 records identified, 263 duplicate or previously screened records removed, 475 unique records screened, 31 reports assessed for eligibility, 10 reports excluded after full-text or report-level assessment, and 21 reports representing 19 independent studies included.

Study selection is summarised in Figure 1. The original searches identified 707 records, and the database update through 24 July 2026 identified 31 additional records: four from PubMed, 27 from Scopus, and none from CENTRAL. Within the updated search, removal of five duplicate occurrences left 26 update-unique records; 22 were already present in the original screening master and four were newly identified. After reconciliation across the original and updated searches, 475 unique records underwent title-and-abstract or registry-record screening. Complete source-level counts and reconciliation are reported in Table S1 (in the ESM).

Among the four newly identified records, Albanell-Fernández et al. proceeded to full-text assessment and met the eligibility criteria [28], whereas the other three were excluded at the title-and-abstract screening stage. Abi Kheir et al. had already been captured in the original screening master and, following reconciliation, proceeded to full-text assessment and was included [29]. Overall, 444 records were excluded before retrieval, and 31 reports were retrieved and assessed at the full-text or report level.

Ten reports were excluded; report-level exclusions and categorised reasons are reported in Table S4 (in the ESM). Twenty-one reports representing 19 independent studies met the eligibility criteria. Five core *Candida* biomarker-guided RCTs and one separate mixed-fungal ACLF RCT comprised the randomised evidence [11,12,13,14,15,30]. Eleven previously identified independent narrative or pathway studies [31,32,33,34,35,36,37,38,39,40,41], together with Albanell-Fernández [28] and Abi Kheir [29], contributed to the structured narrative synthesis. Linked reports derived from the Posteraro and CandiSep populations were retained for unique ancillary information but were not counted as additional independent studies [42,43]. The excess of reports over studies reflects two linked analyses: Standl et al. analysed the *CandiSep* population and Giacobbe et al. re-analysed the Posteraro cohort; neither added an independent study population [42,43].

### 3.1. Evidence Base and Synthesis Roles

Included studies were classified according to the therapeutic action encoded by the biomarker-linked strategy. Initiation/pre-emptive *Candida* algorithms used biomarker positivity to start or accelerate antifungal therapy, whereas exposure-reduction algorithms used negative or serial results, together with clinical reassessment, to support treatment interruption or discontinuation [11,12,13,14,15].

The mixed-fungal ACLF trial evaluated a distinct confirmation-dependent management pathway and was therefore analysed separately from the *Candida*-focused evidence [30]. LAMBDA evaluated a prospective single-arm cover-then-reassess strategy and contributed feasibility evidence only [33]. Other non-randomised studies contributed diagnostic-probability, implementation, or pathway evidence without providing randomised policy effects.

These therapeutic-action categories defined the synthesis strata; initiation, withholding, discontinuation, and de-escalation strategies were not pooled across clinically different decision rules. Table 1 summarises the randomised biomarker-linked strategies and their analytic roles, while complete study-level characteristics and synthesis classifications are reported in Table S5 (in the ESM).

### 3.2. Risk of Bias and Certainty Framework

Risk of bias varied by study design and outcome. Mortality was objective and measured consistently in most core randomised trials. Exposure outcomes were more susceptible to open-label management, clinician adherence and algorithm activation. Mycological safety outcomes were limited by sparse events, different definitions and unequal follow-up windows.

Non-randomised pathway and stewardship studies were used to describe feasibility, implementation and associations, not causal policy effects. Major limitations were confounding by indication, historical-period confounding, selection for biomarker testing, clinician override, heterogeneous thresholds, variable turnaround, incomplete implementation fidelity and incomplete capture of downstream fungal safety events.

GRADE certainty was low for mortality under *Candida* exposure-reduction strategies because imprecision was very serious, and very low for invasive *Candida* events diagnosed after randomisation because of the risk of bias, indirectness across definitions/windows, and extreme imprecision. Certainty was moderate for the direction of increased systemic treatment under initiation/pre-emptive strategies. Certainty for antifungal exposure reduction was low because the effects were inconsistent, endpoints were not combinable, and the small evidence base included a null, low-activation algorithm. CandiSep mortality and the separate ACLF mortality comparison were of low certainty. The descriptive mortality aggregation across opposing rule directions was not assigned a unitary rating. Risk-of-bias judgements are presented in Supplementary Table S6. Detailed outcome-level GRADE judgements, reasons for downgrading, and corresponding absolute-effect estimates are reported in Table S7 (in the ESM).

### 3.3. Primary Randomised Candida Synthesis

**Figure 2:**
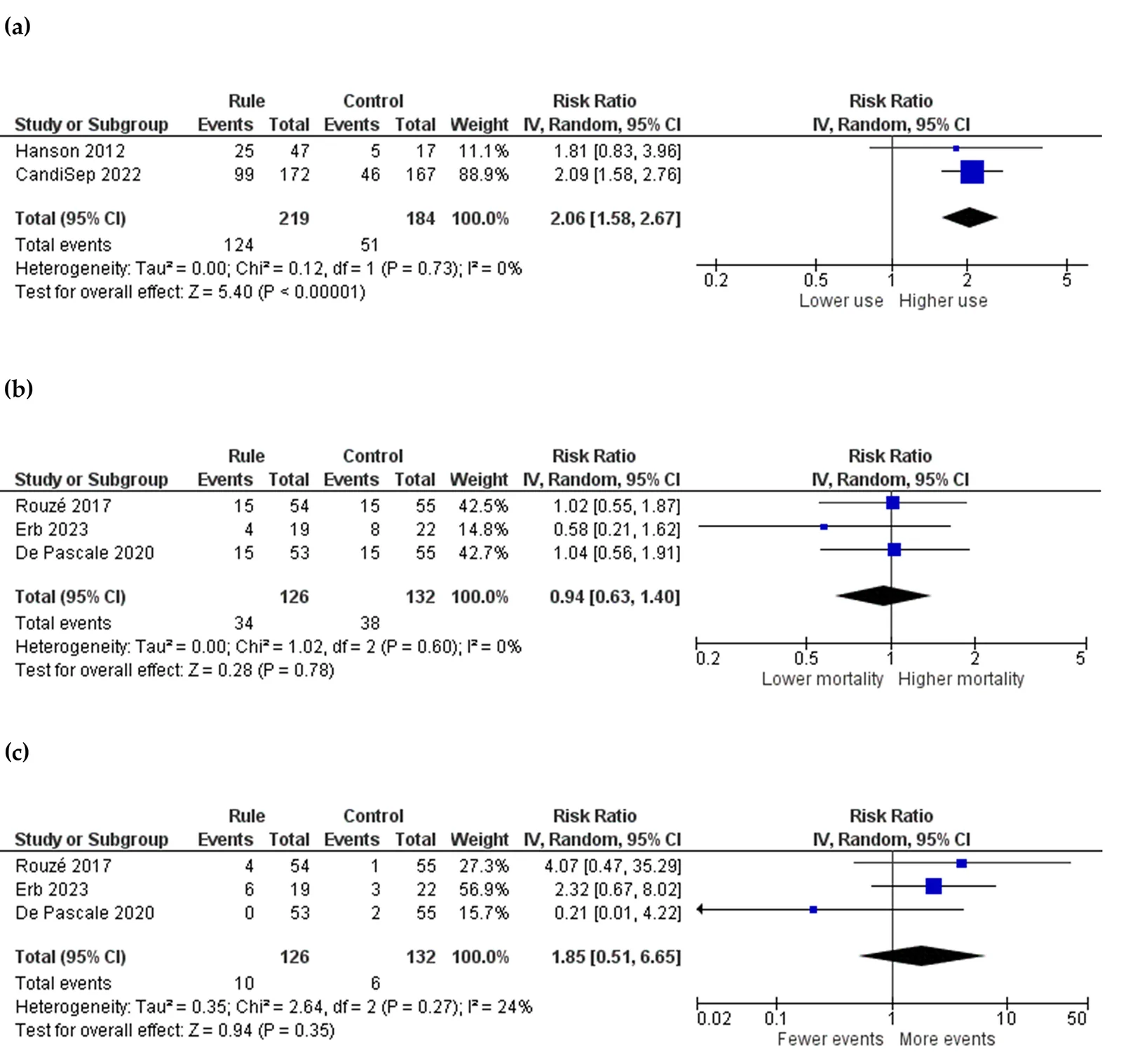
Direction-specific randomised *Candida* evidence. Three-panel forest plot of direction-specific randomised *Candida* evidence. Panel (**a**) shows increased systemic antifungal receipt under rule-in assignment (RR 2.06, 95% CI 1.58–2.67). Panel (**b**) shows short-term mortality under rule-out assignment (RR 0.94, 95% CI 0.63–1.40). Panel (**c**) shows post-randomisation invasive *Candida* events under rule-out assignment (RR 1.85, 95% CI 0.51–6.65). CandiSep 28-day mortality is reported in the text and Table 2 rather than as a single-study forest panel [11,12,13,14,15].

The five core trials included two initiation/pre-emptive and three discontinuation, interruption or early-termination strategies [11,12,13,14,15]. They all enrolled critically ill adults and were *Candida*-focused, but they tested different therapeutic decisions; mortality was therefore synthesised according to rule direction rather than as one generic biomarker effect. Quantitative results are reported in Table 2 and Figure 2. Extracted event counts, study-level effect estimates, primary pooled analyses, and robustness analyses are reported in Table S8 (in the ESM).

Mortality was not pooled across opposing rule directions in the primary presentation. In the CandiSep rule-in trial, death by day 28 occurred in 58/172 versus 51/167 participants (recalculated crude RR 1.10, 95% CI 0.81–1.51). The estimate neither demonstrated mortality benefit nor excluded clinically relevant harm [12]. The descriptive aggregation across CandiSep and the three rule-out trials are reported only in Table S8 (in the ESM).

In the decision-specific exposure-reduction subgroup, death occurred in 34/126 patients assigned to biomarker-guided management and in 38/132 controls [11,13,14]. The pooled RR was 0.94 (95% CI 0.63–1.40; I^2^ = 0%; *p* = 0.78; Figure 2b). The interval ranged from a 37% relative reduction to a 40% increase in mortality. Because these trials were not non-inferiority studies, mortality neutrality was not established.

Antifungal exposure showed the clearest rule-dependent pattern. Positive-result initiation increased systemic antifungal receipt (124/219 versus 51/184; RR 2.06, 95% CI 1.58–2.67; I^2^ = 0%; *p* < 0.001; Figure 2a), corresponding to 293 additional recipients per 1000 at the pooled control risk (95% CI 162–463 more) [12,15]. Hanson and CandiSep both increased treatment exposure, although the magnitude depended on the trigger and sampling strategy. Trial-level treatment thresholds, timing, and diagnostic-performance details are summarised in Table 1 and Table S8 (in the ESM).

Stopping strategies produced heterogeneous reductions in antifungal exposure. Rouzé and De Pascale achieved substantial treatment separation, whereas the Erb all-negative gate rarely became actionable and produced little difference in antifungal consumption [11,13,14]. This cross-trial pattern suggests that gate activation and delivery are important determinants of stewardship effect, although these comparisons were not randomised and should not be interpreted as evidence of effect modification. Trial-level activation, adherence, and exposure data are reported in Table 1 and Table S8 (in the ESM). The observed delivered-stop proportions ranged from 2/19 (10.5%) in Erb to 29/54 (53.7%) in Rouzé and 37/53 (69.8%) by day 5 in De Pascale; these cross-trial differences are descriptive and do not define a validated activation threshold.

Policy-level fungal safety was the least certain randomised endpoint. Invasive *Candida* events diagnosed after randomisation under rule-out assignment occurred in 10/126 versus 6/132 patients. Only infections first diagnosed after randomisation or explicitly reported as subsequent/follow-up entered this pool; in Rouzé, the prespecified proven-or-probable endpoint was used (4/54 versus 1/55), and in De Pascale, 11 infections present at enrolment (6/53 versus 5/55) were excluded while the two follow-up infections (0/53 versus 2/55) were counted [11,13,14]. The pooled RR was 1.85 (95% CI 0.51–6.65; I^2^ = 24.3%; *p* = 0.35; Figure 2c). This intention-to-treat contrast is a policy-assignment effect, not the safety of discontinuation among patients whose gate opened. With 16 events, heterogeneous definitions and windows, and markedly different gate activation, no clinically acceptable fungal-safety margin can be inferred.

Sensitivity analyses using REML/Wald, modified HKSJ, Mantel–Haenszel and risk-difference models all remained compatible with benefit and harm; the proven-only Rouzé sensitivity yielded RR 1.65 (95% CI 0.54–4.98). No estimator resolved the precision limit imposed by 16 events (Table S8 in the ESM). A post hoc sensitivity analysis excluding the very low-activation Erb trial yielded an RR of 1.03 (95% CI 0.67–1.58) for rule-out mortality and an RR of 1.12 (95% CI 0.06–20.13) for post-randomisation invasive *Candida* events. The substantive conclusion was unchanged, while fungal-safety uncertainty became even wider.

Leave-one-out and bounded missing-outcome analyses for De Pascale yielded RRs ranging from 0.82 to 1.09, with all confidence intervals crossing the null; mortality uncertainty therefore remained unresolved (Table S8 in the ESM) [13].

Individual trial safety observations were sparse and could not establish comparative safety. Notably, in Erb, one of the two patients who stopped therapy after initially negative biomarkers subsequently developed candidemia and required treatment restart, illustrating the importance of serial surveillance and rescue [11]. Additional trial-level safety events are reported in Table 1 and Table S8 (in the ESM).

Adverse-event, ecological, resistance, and cost outcomes were too sparsely reported for synthesis. Hanson recorded 15 possibly drug-related events in 10/21 participants receiving pre-emptive anidulafungin, none of which were serious [15]. CandiSep reported median antifungal costs of €4451 versus €2800 per patient (*p* = 0.52) [12]. No core trial quantified resistance selection or ecological consequences.

### 3.4. Separate Mixed-Fungal Randomised Evidence

One additional pragmatic randomised trial enrolled 216 adults with ACLF, multiple fungal-risk factors, and clinical suspicion of invasive fungal infection [30]. The confirmation-dependent arm could initiate therapy after mycological or radiological evidence, a single BDG value ≥ 150 pg/mL or repeated values ≥80 pg/mL, a single galactomannan index ≥ 1.0 or repeated values ≥ 0.5, or other prespecified evidence. Thus, the contrast was a bundled diagnostic-management strategy, not one biomarker. Antifungals began a median of 0 versus 3 days after randomisation; 107/108 versus 89/108 patients were treated, and the median duration among treated participants was 8 days in both arms. The principal separation was early delay or non-initiation, not prolonged treatment duration. This trial was retained to inform the effect of a complete confirmation-dependent mixed-fungal strategy within its own high-risk liver-ICU population, not to derive *Candida*-specific stewardship recommendations or to support extrapolation to lower-risk ICU populations.

At 28 days, survival was 38/108 (35.2%) with immediate empirical therapy and 14/108 (13.0%) with confirmation-dependent pre-emptive therapy; the corresponding mortality rates were 70/108 (64.8%) and 94/108 (87.0%), respectively [30]. Expressed as death, the crude RR for pre-emptive versus empirical therapy was 1.34 (95% CI 1.15–1.57), with an absolute increase of 22.2 percentage points (Newcombe 95% CI 10.9–32.9). The reported mortality HR for empirical versus pre-emptive therapy was 0.64 (95% CI 0.47–0.88). Treatment success among treated participants was post-randomisation and was not interpreted causally.

### 3.5. Candida-Focused and Mixed ICU Pathway Evidence

*Candida*-focused non-randomised studies primarily informed post-test probability, pathway timing, implementation, and feasibility rather than causal treatment effects. In the Helweg–Larsen programme, a negative combined T2MR/mannan result reduced modelled invasive candidiasis probability from 12% to 3% in one ICU setting and from 28% to 10% in a higher-prevalence setting [35]. Observational discontinuation pathways in Nucci, Hare, and Gill were associated with earlier antifungal discontinuation in selected implementation settings, but these findings are hypothesis-generating: confounding by indication, clinician behaviour, co-interventions, and temporal trends preclude causal inference on efficacy or safety [36,37,38]. Detailed implementation, exposure, and safety outcomes are reported in Table S2 (in the ESM).

LAMBDA evaluated a different cover-then-reassess strategy: all 40 high-risk postoperative patients received liposomal amphotericin B on day 1, and 26/40 discontinued therapy on day 3 after a negative baseline BDG result [33]. Without a concurrent comparator, the study demonstrates operational feasibility but does not estimate comparative efficacy, safety, or net antifungal exposure.

### 3.6. Linked Candida Diagnostic-Performance Evidence

The linked CandiSep diagnostic-performance analysis contributed no additional participants or treatment-policy effect. Antigen assays, particularly BDG and mannan, were more informative than anti-*Candida* antibody assays, but manufacturer cutoffs performed suboptimally, optimised thresholds were cohort-dependent, and assay combinations showed no clear advantage over selected single tests [42]. Diagnostic estimates were imprecise because only 48 invasive *Candida* infections and 14 candidemias were available; detailed results are reported in Table S2 (in the ESM). This was a secondary linked diagnostic analysis and did not contribute to the primary randomised policy-effect conclusions.

### 3.7. Recent Candida Implementation Evidence

Two recent non-randomised implementation studies provided hypothesis-generating signals regarding timely results and pathway delivery, rather than causal treatment-effect evidence. In the study by Albanell-Fernández et al., BDG-negative patients were more frequently discontinued early and had shorter antifungal exposure, although two patients with invasive candidiasis initially had negative BDG results [28]. In Abi Kheir, the pharmacy-driven programme did not reduce overall micafungin duration, but shorter exposure was observed when BDG results were available within 48 h and negative results were followed by protocol-concordant discontinuation [29]. Both studies were non-randomised and therefore identify implementation signals rather than causal efficacy or safety; detailed exposure, adherence, and clinical outcomes are reported in Table S2 (in the ESM).

### 3.8. Beyond Candida: Decision Signals Without Validated Treatment Rules

Beyond *Candida*, non-randomised studies linked panfungal BDG to treatment initiation or discontinuation, tracheal aspirate galactomannan to pre-emptive treatment in a CAPA-risk pathway, and BAL mNGS to organism identification and clinician-selected regimen adjustment [31,34,41]. These studies provided diagnostic-management and implementation signals, but none randomised the biomarker-linked action or established a causal exposure–safety trade-off. Study-level diagnostic and clinical results are reported in Table S2 (in the ESM). No eligible ICU study tested a prespecified therapeutic policy for Pneumocystis, cryptococcosis, mucormycosis, or endemic mycoses.

## 4. Discussion

This review shifts the unit of inference from the assay to the decision rule. Across the randomised *Candida* evidence, positive-result strategies consistently increased antifungal treatment, whereas negative-result strategies reduced exposure only when the stopping gate became actionable and the intended intervention was delivered. Neither direction established patient benefit or fungal safety: mortality remained imprecise and only 16 post-randomisation invasive *Candida* events informed the rule-out safety analysis. Stewardship success should therefore be judged direction-specifically, balancing earlier appropriate therapy against overtreatment for rule-in and exposure reduction against prespecified fungal-safety margins for rule-out [11,12,13,14,15]. No validated numerical fungal-safety margin was identified in the evidence base.

The principal design error exposed by the full texts is to select a laboratory cutoff and then treat it as a therapeutic threshold. An assay cutoff describes an operating point for discrimination; a treatment threshold additionally depends on pre-test probability and the unequal consequences of delayed therapy, unnecessary exposure, toxicity, ecological pressure and diagnostic delay. Classical threshold theory and decision-curve methods formalise this distinction: clinical utility depends on whether a result moves estimated risk across an action threshold, not on sensitivity, specificity or area under the curve alone [44,45].

At the bedside, this produces three zones rather than a single positive/negative split. Above a treatment boundary, therapy and diagnostic work-up proceed in parallel. Below a stop-eligibility boundary, a timely negative result can support stopping empirical therapy only when clinical work-up and trajectory are concordant; the randomised evidence did not validate initial withholding. Between the two lies an uncertainty zone for repeat or orthogonal testing, cultures, imaging, source assessment and short-interval reassessment. The assay updates risk; it does not choose the action [44,45].

In the evaluated stopping pathways, rule-out functioned as a conjunctive gate rather than as a synonym for a single negative result. Eligibility, timely valid testing, concordant clinical reassessment, and capacity for surveillance and rescue all contributed to whether stopping became appropriate. Serial testing adds information but may also reduce gate activation when any positive result vetoes discontinuation; conversely, requiring repeated positivity for rule-in may reduce overtreatment at the cost of delayed action. Number, timing, and AND/OR logic should therefore be considered components of the treatment policy rather than properties of the assay alone [11,13,14,15,42].

Timing and baseline risk jointly determine whether a biomarker result can alter management. Sampling too early may provide false reassurance, whereas reporting too late makes even an accurate result therapeutically irrelevant. The acceptable interval for diagnostic confirmation also narrows as untreated risk increases; the adverse outcome of the confirmation-dependent ACLF strategy illustrates the potential cost of delay in a highly selected high-risk population without establishing that confirmation-dependent care is universally harmful [11,12,28,29,30,37].

Execution is therefore part of the intervention: eligibility → valid sample → timely result → gate activation → recommendation → delivered action → surveillance and, when necessary, rescue. Failure at any node can attenuate treatment separation. Intention-to-treat estimates should remain primary because analyses restricted to activated, adherent, or discontinued patients select post-randomisation subgroups; activation, override, restart, and rescue are better treated as mechanism and safety measures unless prespecified causal methods are used [11,12,13,14,28,29,36,37]. These cross-cutting decision-rule components, their clinical meaning, and the corresponding implications for trial design and reporting are summarised in Table 3. No minimum clinically useful gate-activation proportion can be inferred from these trials. The near-10% delivered stopping rate in Erb produced little population-level treatment separation, whereas Rouzé and De Pascale achieved substantially higher delivered-stop rates and larger exposure separation; however, these were not randomised comparisons of activation thresholds. Future trials should therefore prespecify the expected actionable fraction and an implementation-futility criterion tied to the treatment separation required for the exposure endpoint, rather than adopt a universal percentage.

### 4.1. Clinical Interpretation Within Current Evidence Limits

**Figure 3:**
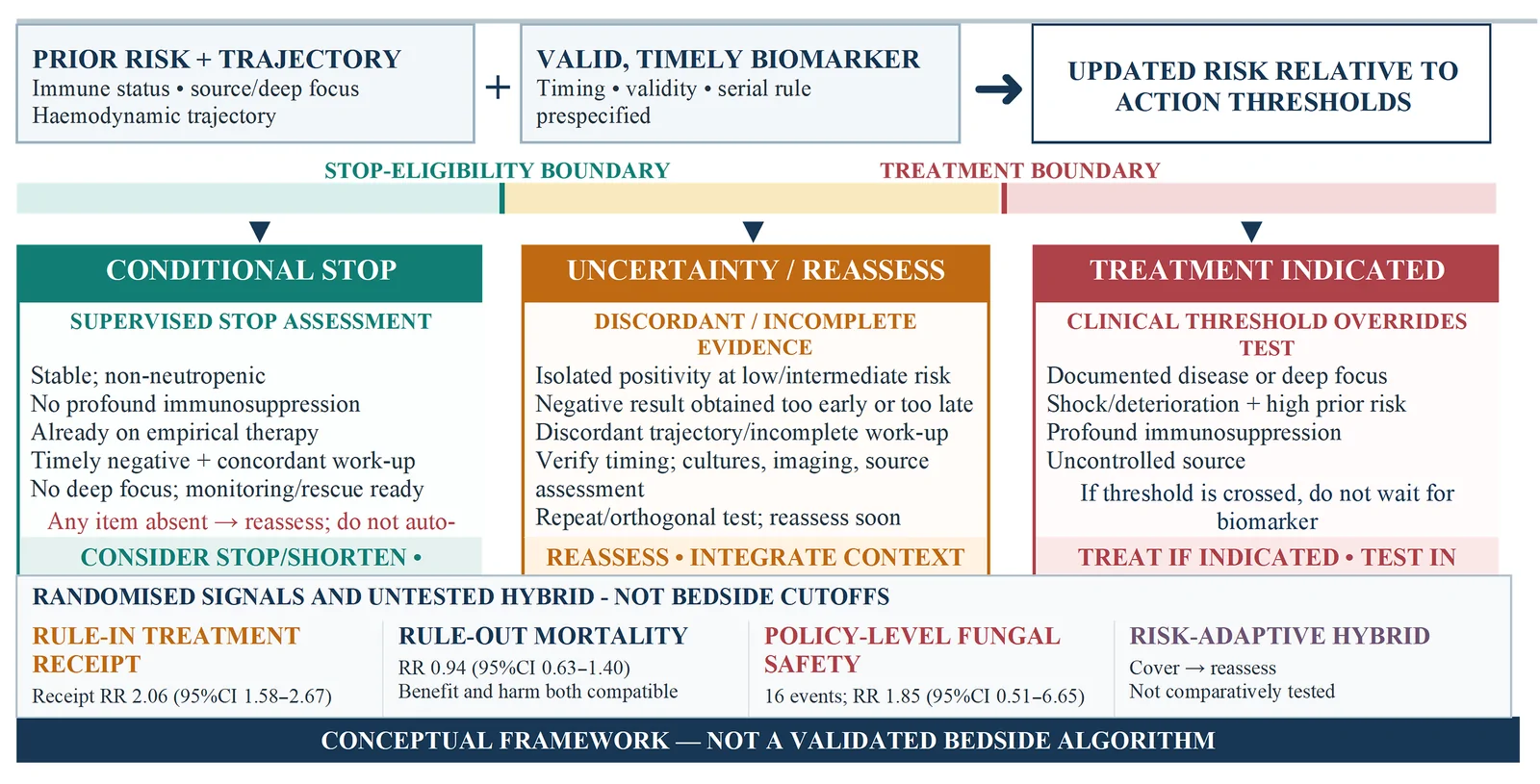
Risk-conditioned architecture for fungal biomarker stewardship. Conceptual three-zone framework for fungal biomarker stewardship. Baseline fungal risk, clinical trajectory, and a timely valid biomarker result determine whether a patient falls within a treatment zone, an uncertainty and reassessment zone, or a conditional supervised stop-assessment zone. The framework emphasises clinical action thresholds rather than assay cutoffs and incorporates timing, concordant diagnostic work-up, monitoring, and rescue capacity. A lower strip summarises randomised rule-in and rule-out signals and identifies a risk-adaptive cover-then-reassess strategy as an untested comparative hypothesis.

Clinically, the three-zone framework should be applied according to the baseline risk, clinical trajectory, and the timing and concordance of the biomarker results (Figure 3). Treatment should not await biomarker confirmation when established disease, rapid deterioration, profound immunosuppression, a deep focus, or an uncontrolled source already places untreated risk above the treatment threshold. Conversely, a timely negative or protocol-defined serial-negative result may support supervised discontinuation in a clinically stable patient already receiving empirical therapy only when the clinical work-up, trajectory, monitoring, and capacity for rapid rescue are concordant. Discordant, delayed, or isolated biomarker results should prompt reassessment and, when appropriate, repeat or orthogonal testing rather than automatic treatment initiation or discontinuation [2,3,4,11,13,14,28,29,35,36,37,38,42].

*CandiSep* illustrates a protocol-specific approach to discordance: any one of two BDG values ≥ 80 pg/mL triggered antifungal initiation; if only one value was positive and blood cultures remained *Candida*-negative, therapy was subsequently discontinued, whereas two positive BDG values led to continuation despite negative blood cultures [12]. Outside a validated protocol, discordance should be treated as an uncertainty state rather than an automatic start/stop signal.

Clinical scores and host-risk features are most useful for estimating prior probability, while biomarkers update that probability. In the Posteraro cohort, BDG was evaluated in septic ICU patients with a *Candida* score ≥ 3 [39], and Helweg–Larsen showed that the same negative combined test produced different post-test risks when the prior risk was 12% versus 28% [35]. Thus, a score should not be added mechanically to a BDG cutoff; the combined information is clinically useful only if it moves the estimated risk across a prespecified treatment or stop threshold [44,45]. No included randomised trial validated a specific clinical-score-plus-BDG composite decision policy.

No included trial establishes the optimal BDG threshold, the number or timing of samples, or the superiority of a biomarker combination. Cohort-derived threshold optimisation cannot be treated as external validation, and the linked CandiSep analysis found no clear diagnostic advantage from combining the evaluated assays. A local pathway can specify the operational values for study or quality-improvement purposes, but those values should be labelled as protocol choices rather than as evidence-proven treatment thresholds [12,42]. The practical question is not “is BDG positive or negative?” but “did the complete, timely evidence move this patient across a prespecified action boundary, and are all safeguards for that action present?”

### 4.2. How the Next Trials Should Be Designed

Future trials should randomise the complete decision policy at the point when the therapeutic action is genuinely contestable. For rule-out strategies, this is early reassessment after empirical therapy has begun rather than ICU admission. Eligibility should reflect the baseline fungal risk, established disease, source control, and immunosuppression. The primary estimand should also match the rule direction: rule-in trials should quantify the all-randomised policy effect while separately describing time to appropriate therapy and overtreatment, whereas rule-out trials should pair antifungal-free days or total antifungal exposure with an adjudicated fungal-safety endpoint.

Safety should determine the sample size for exposure-reduction trials, with the upper confidence bound judged against a prespecified clinically acceptable absolute margin rather than relying on a non-significant *p*-value. Surveillance, adjudication, and follow-up should be comparable between groups, with baseline disease distinguished from infection first detected after assignment and restart, rescue, and breakthrough infection explicitly reported. Threshold development should precede policy evaluation: assay calibration and post-test risk should be validated in the intended population, and the cutoff, sampling schedule, and logical operator should be locked before confirmatory testing rather than selected retrospectively [44,45]. For trial planning only, the RR 1.25 “appreciable harm” threshold used in our GRADE/OIS imprecision audit can serve as a provisional benchmark rather than a bedside cutoff. At the pooled rule-out control risk of 4.5%, RR 1.25 corresponds to an absolute increase of approximately 1.1 percentage points. This benchmark is not validated as a universal clinical margin and should be prespecified according to baseline risk, disease severity, follow-up, and rescue capacity; the current confidence and risk-difference intervals do not exclude substantially larger harm.

Trials should report denominators and timing across the full pathway (from eligibility and valid sampling to actionable results, gate activation, recommendation, override, delivered action, and rescue) so that implementation failure can be distinguished from biological failure. Evidence from rapid phenotypic antimicrobial susceptibility testing similarly illustrates that faster diagnostic information has clinical value only when delivered in time to modify treatment, while high-dimensional decision-support models highlight the need for external validation, calibration, and prospective impact assessment before integration into clinical pathways [46,47]. A risk-adaptive cover-then-reassess strategy remains an important comparative hypothesis: LAMBDA demonstrates feasibility of early cover followed by biomarker-guided reassessment in one selected cohort, whereas the ACLF trial illustrates the potential cost of confirmation-dependent delay in a high-risk population. Neither establishes the effect of the hybrid; a definitive trial should compare complete pathways and jointly evaluate time to initial therapy, downstream antifungal exposure, fungal safety, and rescue [30,33].

### 4.3. Beyond Candida

Beyond the separate mixed-fungal ACLF strategy trial, pathogen-specific non-*Candida* evidence was non-randomised and highly context-specific; the decision-rule framework may nevertheless be conceptually transferable. Panfungal BDG, respiratory galactomannan, and BAL mNGS address different treatment decisions and therefore cannot support a single transferable antifungal rule; none of the included studies randomised the biomarker-linked action [31,34,41]. Evidence from highly selected populations, including patients receiving ECMO, further illustrates how baseline risk, co-infection, and antifungal-pharmacology uncertainties may limit transportability [48]. Accordingly, *Candida*-derived BDG start/stop thresholds should not be extrapolated to mould disease. For suspected invasive aspergillosis, respiratory galactomannan, *Aspergillus* PCR, or mNGS should be interpreted within a pathogen-specific pathway integrating host factors, imaging, conventional microbiology, timing, and clinical trajectory; the included evidence did not validate a randomised biomarker-triggered start/stop policy. For mucormycosis, no eligible ICU study tested a prespecified biomarker-linked therapeutic policy, so this review cannot support biomarker-based withholding or discontinuation recommendations.

### 4.4. Strengths and Limitations

A principal strength of this review is its decision-based synthesis across 21 reports representing 19 independent studies, preserving report-to-study linkage and avoiding double-counting. Initiation and stopping strategies were not averaged into a generic biomarker effect, randomised policy effects were separated from diagnostic accuracy and implementation associations, and full-text data were used to characterise threshold, seriality, timing, gate activation, adherence, and rescue.

The evidence base nevertheless limits operationalisation. The five core *Candida* RCTs were small and open-label, exposure endpoints were not construct-equivalent, gate activation varied, and fungal events were sparse and variably defined. Few-study and sparse-event meta-analysis therefore remained imprecise: sensitivity analyses supported treatment expansion under rule-in but could not establish a decision-grade fungal-safety margin from only 16 post-randomisation events. Cross-study comparisons cannot determine interactions among threshold, seriality, baseline risk, and turnaround time, and no trial directly compared alternative logical operators or a risk-adaptive hybrid. Accordingly, the review cannot identify a universal assay, cutoff, sampling interval, or biomarker combination. Observational implementation evidence is also vulnerable to confounding by programme intensity, clinician behaviour, and laboratory throughput, while resistance, ecological consequences, avoided toxicity, costs, and implementation burden were incompletely measured. Formal assessment of publication bias or small-study effects was not feasible because every pooled outcome included fewer than ten randomised comparisons. Publication and selective-reporting bias therefore cannot be excluded, particularly given the small, predominantly open-label randomised evidence base and the additional limitations of non-randomised studies.

## 5. Conclusions

Fungal biomarker stewardship should be understood as a risk-conditioned, time-bounded decision policy rather than treatment by laboratory cutoff. Randomised evidence shows a clear prescribing effect: positive-result rules increase antifungal use without demonstrated patient benefit, whereas negative-result strategies can reduce exposure only when timely results make the stopping rule actionable and the intended intervention is delivered. The available evidence does not establish isolated positive BDG as a general stand-alone treatment trigger or the safety of autonomous biomarker-guided discontinuation. A negative result may instead support supervised reassessment and discontinuation in appropriately selected patients with concordant clinical work-up, monitoring, and rapid rescue, while treatment should not await biomarker confirmation once the clinical treatment threshold has been crossed. Future trials should randomise the complete decision pathway and jointly evaluate appropriate treatment, antifungal exposure, and adjudicated fungal safety.

## Figures and Tables

**Table 1:** Randomised biomarker decision rules and their clinical consequences.

Study/Reference	Design and Population	Biomarker Decision Rule	Prescribing Consequence	Patient-Important/Fungal Outcome	Contribution to Synthesis
Hanson 2012 [15]	**Open-label pilot RCT.** 64 randomised/analysed ICU adults at increased invasive candidiasis risk; 3:1 allocation.	**Rule-in.** BDG twice weekly; a subsequent value ≥ 60 pg/mL triggered pre-emptive anidulafungin.	**Treatment receipt.** 25/47 versus 5/17; protocol anidulafungin in 21/47.	ICU-discharge survival 85% versus 81%; exact event counts were not reported and were not reconstructed.	Rule-in treatment-receipt pool; mortality narrative only.
Rouzé 2017 [14]	**Open-label RCT.** 110 randomised; 109 analysed mixed-ICU adults already receiving empirical antifungals.	**Rule-out.** BDG, mannan and anti-mannan on days 0 and 4; a negative multimarker algorithm recommended stopping before day 7.	**Gate and delivery.** 32/54 recommendations; 29 followed. Early stop 29/54 versus 1/55; duration 6 versus 13 days.	28-day deaths 15/54 versus 15/55; subsequent proven or probable invasive *Candida* 4/54 versus 1/55.	Rule-out mortality and fungal-safety pools; exposure narrative.
De Pascale 2020 [13]	**Open-label RCT.** 120 randomised; 108 included in the available-case analysis after post-randomisation exclusions.	**Rule-out.** BDG at enrolment and every 48–72 h; the first negative result (<80 pg/mL) triggered interruption.	**Stopping.** Stopped by day 5 in 37/53; median duration 2 versus 10 days.	30-day deaths 15/53 versus 15/55; subsequent invasive *Candida* 0/53 versus 2/55.	Rule-out mortality and fungal-safety pools; exposure narrative.
CandiSep 2022 [12]	**Open-label multicentre RCT.** 342 randomised; 339 analysed adults with sepsis and predefined invasive *Candida* risk factors.	**Rule-in.** Two BDG samples in the first 2 study days; any value ≥ 80 pg/mL triggered initiation; discordant serial results followed protocol.	**Treatment acceleration.** Any trial antifungal 99/172 versus 46/167; within 96 h, 84/172 versus 10/167.	28-day deaths 58/172 versus 51/167.	Rule-in treatment-receipt and mortality analyses; linked diagnostic report added no participants.
Erb 2023 [11]	**Open-label RCT.** All 41 randomised ICU adults analysed.	**Rule-out with OR-positive veto.** BDG and mannan on days 1 and 2; all four measurements needed to be negative.	**Gate collapse.** 32/41 had ≥1 positive early marker; 17/19 intervention patients failed the gate and only 2/19 stopped; 11.3 versus 10.5 DDD.	28-day deaths 4/19 versus 8/22; proven *Candida* 6/19 versus 3/22. One stopped patient developed candidemia and restarted therapy.	Rule-out mortality and fungal-safety pools; implementation signal.
Verma/ACLF 2025 [30]	**Pragmatic RCT.** 216 adults with ACLF, multiple invasive fungal risks and very high short-term mortality.	**Bundled mixed-fungal strategy.** Immediate empirical treatment versus a confirmation-dependent pathway using biomarkers, cultures, imaging and prespecified criteria.	**Timing and receipt.** Median start 0 versus 3 days; treated 107/108 versus 89/108; median duration among treated patients 8 days in both groups.	Deaths 70/108 versus 94/108; confirmation-dependent versus empirical RR 1.34 (95% CI 1.15–1.57).	Separate high-risk randomised evidence; not pooled with *Candida* trials.

The five core *Candida* trials randomised 677 participants; 661 entered the published analysis populations. The ACLF trial is a separate mixed-fungal, high-risk strategy comparison and was not pooled with *Candida* evidence. Cross-trial differences are descriptive and were not tested as interactions. BDG, β-(1→3)-D-glucan; DDD, defined daily dose; RCT, randomised controlled trial.

**Table 2:** Direction-specific randomised estimands, robustness, and clinical interpretation.

Estimand/Reference	Evidence Base	Primary Estimate	Robustness/Absolute Effect	GRADE Certainty	Decision Message
Rule-out: short-term mortality [11,13,14]	3 RCTs; 258 analysed; 34/126 versus 38/132 deaths	**RR 0.94** (95% CI 0.63–1.40); I^2^ = 0%	REML/Wald 0.94 (0.63–1.40); mHKSJ 0.94 (0.39–2.26); missing-outcome bounds 0.82–1.09.	**Low**	Neither benefit nor harm was excluded; the stopping policy cannot be labelled mortality-neutral.
Rule-out: post-randomisation invasive *Candida* events [11,13,14]	3 RCTs; 258 analysed; 10/126 versus 6/132 events	RR 1.85 (95% CI 0.51–6.65); I^2^ = 24.3%	REML/Wald 2.00 (0.72–5.50); mHKSJ 2.00 (0.15–25.80); MH RR 1.83 (0.71–4.71); DL RD +2.4% (−6.8% to +11.7%).	**Very low**	Only 16 events: the fungal-safety signal is highly imprecise and an acceptable safety margin has not been established.
Rule-in: systemic antifungal receipt [12,15]	2 RCTs; 403 analysed; 124/219 versus 51/184 treated	**RR 2.06** (95% CI 1.58–2.67); I^2^ = 0%	Alternative RR 2.05–2.06; mHKSJ 2.06 (0.38–11.21); +293/1000 (+162 to +463).	**Moderate (direction only)**	Rule-in consistently expands exposure; certainty concerns the direction of increased receipt, not the precise pooled magnitude or patient benefit.
CandiSep: 28-day mortality [12]	1 RCT; 339 analysed; 58/172 versus 51/167 deaths	**RR 1.10** (95% CI 0.81–1.51)	Single-study estimate; no pooling.	**Low**	Positive-result assignment did not demonstrate a mortality benefit.
CandiSep: treatment within 96 h [12]	1 RCT; 339 analysed; 84/172 versus 10/167 treated	**RR 8.16** (95% CI 4.39–15.16)	Single-study estimate; no pooling.	Not separately graded	Quantifies the treatment acceleration produced by the positive-result trigger.
ACLF: confirmation-dependent versus empirical [30]	1 RCT; 216 analysed; 94/108 versus 70/108 deaths	**RR 1.34** (95% CI 1.15–1.57)	RD +22.2 percentage points (+10.9 to +32.9); inverse contrast HR 0.64 (0.47–0.88).	**Low**	A bundled high-risk strategy effect—not an isolated assay effect; do not extrapolate to lower-risk *Candida* stewardship.

Risk ratios > 1 indicate more deaths, fungal events, or antifungal receipt in the decision-rule arm, according to outcome. Modified HKSJ intervals are conservative stress tests and are not used mechanically when k = 2. De Pascale missing-outcome bounds restore all 120 randomised participants under the prespecified scenarios in Supplementary Table S8. For rule-in treatment receipt, the moderate GRADE rating applies to the direction of increased receipt, not to the pooled magnitude or transportability. CI, confidence interval; DL, DerSimonian–Laird; HKSJ, Hartung–Knapp–Sidik–Jonkman; MH, Mantel–Haenszel; RD, risk difference; REML, restricted maximum likelihood; RR, risk ratio.

**Table 3:** Decision-rule architecture: full-text signals, bedside meaning, and trial implications.

Algorithm Element	Evidence Type and Full-Text Signal	Clinical Meaning	Required Trial/Reporting Element
Rule direction	RCT signal: rule-in increased treatment receipt (RR 2.06, 95% CI 1.58–2.67); Rouzé and De Pascale reduced exposure, whereas the Erb gate rarely opened [11,12,13,14,15].	Rule-in expands exposure; rule-out reduces it only when the gate opens and the intended action is delivered. Patient benefit and fungal safety remain unestablished.	Use direction-specific estimands: appropriate early therapy/overtreatment for rule-in; exposure reduction plus a prespecified fungal-safety margin for rule-out.
Eligibility and spectrum	Transportability signal. CandiSep enrolled only 14.7% of screened patients; abdominal surgery predominated and may elevate BDG. [12]	Accuracy and treatment separation depend on who enters the pathway.	Report screened, eligible, sampled and analysed denominators separately.
Assay cutoff versus action threshold	CandiSep used BDG ≥ 80 pg/mL as a treatment trigger; a linked cohort derived > 280 pg/mL at 80% specificity but 46% sensitivity. Hanson modelled a stricter repeated-positive rule [12,15,42].	A diagnostic operating point is not a treatment threshold; prior risk and false-positive/false-negative costs matter.	Prespecify risk stratum, treatment threshold and uncertainty zone; validate calibration and net benefit.
Seriality and logical operator	Erb required all four day-1/day-2 BDG and mannan results to be negative; 17/19 failed the gate and one stopped patient later developed candidemia [11].	Seriality can detect late signal, but an any-positive veto may close the gate and delay action.	Prespecify sample number, timing and AND/OR logic; report gate-open fraction, vetoes, false-negative stops and delay.
Gate activation and adherence	Gate/delivery: Rouzé 29/54 stopped; De Pascale 37/53 stopped by day 5; Erb 2/19 stopped. Recent non-randomised pathways also showed variable acceptance and delivery [11,13,14,28,29].	Exposure falls only when the gate opens and the intended action is delivered; override occurs in both directions.	Report activation, vetoes, overrides, adherence and delivered action.
Two decision clocks	Timing signal: T2MR versus BDG turnaround was 0.4 versus 3.8 days; in Abi Kheir, mean BDG turnaround was 60 h and timely-result comparisons were non-randomised [11,12,29,37].	A result arriving after the target decision is inert; an early negative may precede detectability.	Separate sample-to-result from result-to-action time and define delayed/invalid handling.
Prior risk and confirmation cost	Prior-risk signal: the same negative combined test modelled risk from 12% to 3% but from 28% to 10%; ACLF therapy began at a median of 0 versus 3 days [30,35].	The same result may cross a stopping threshold at one prior risk but not another; ACLF tested a bundled pathway.	Stratify prior risk and prespecify the maximum acceptable confirmation interval.
Risk-adaptive hybrid	LAMBDA used immediate cover then day-3 BDG reassessment in a single-arm cohort; ACLF tested a different bundled confirmation-dependent strategy [30,33].	Cover-then-reassess was feasible in one selected cohort but is untested as a comparative risk-adaptive pathway.	Randomise the complete pathway and prespecify delay, later exposure and fungal-safety margins.
Policy effect and delivery	Policy pathway: eligibility → valid sample → timely result → gate → recommendation → acceptance/override → action → surveillance/rescue. Observational delivery comparisons are non-causal [11,12,13,14,28,29,36,37].	Intention-to-treat estimates assignment to the policy; restricting the analysis to patients who stopped or adhered selects post-randomisation subgroups.	Keep intention-to-treat primary; report every denominator and interval; treat activation, adherence and rescue as mechanisms unless causal estimands are prespecified.

Conceptual synthesis; no head-to-head comparison. Observational implementation estimates are non-causal. T2MR, T2 magnetic resonance *Candida* panel.

## Data Availability

All aggregate randomised event counts, study-level effects, model sensitivities, and pooling decisions are reported in Table S8 (in the ESM). Search and PRISMA reconciliation, extraction, statistical rules, exclusions, study characteristics, risk of bias, and GRADE are reported in Supplementary Tables S1–S7. No individual participant data were used.

## Supplementary Materials

The following supporting information can be downloaded at: https://www.mdpi.com/article/10.3390/jof12090681/s1, Table S1: Search Strategies, Source Accounting, and PRISMA 2020 Reconciliation; Table S2: Data-Extraction Framework and Structured Narrative/Diagnostic Outcome Extraction; Table S3: Statistical Rules, Endpoint Definitions, Pooling Criteria, and Sensitivity Analyses; Table S4: Reports Excluded after Full-Text or Report-Level Eligibility Assessment; Table S5: Characteristics of Included Reports and Synthesis Classification; Table S6: Outcome-Specific Risk-of-Bias Judgements and Design-Appropriate Appraisal; Table S7: GRADE Summary of Findings and Certainty Assessment; Table S8: Randomised Event Counts, Study-Level Effects and Sensitivity Analyses.

## Abbreviations

ACLF	acute-on-chronic liver failure
BDG	β-(1→3)-D-glucan
CAPA	COVID-19-associated pulmonary aspergillosis
CI	confidence interval
DDD	defined daily dose
GRADE	Grading of Recommendations Assessment, Development and Evaluation
IC	invasive candidiasis
ICI	invasive *Candida* infection
ICU	intensive care unit
IFI	invasive fungal infection
L-AmB	liposomal amphotericin B
mHKSJ	modified Hartung–Knapp–Sidik–Jonkman
mNGS	metagenomic next-generation sequencing
PCR	polymerase chain reaction
PRISMA	Preferred Reporting Items for Systematic Reviews and Meta-Analyses
RCT	randomised controlled trial
REML	restricted maximum likelihood
RoB	risk of bias
RR	risk ratio
SWiM	Synthesis Without Meta-analysis
T2MR	T2 magnetic resonance
