# Supplementary material for: Decision-Rule Architecture in Fungal Biomarker-Guided Antifungal Stewardship for Critically Ill Adults: A Systematic Review and Candida-Focused Randomised Meta-Analysis
Source: J Fungi (Basel). 2026 Sep 10;12(9):681. doi: 10.3390/jof12090681 (PMC13608780; doi:10.3390/jof12090681)
Supplement: Supplementary file 1 [file jof-12-00681-s001.zip › jof-4531269-supplementary.pdf]

ELECTRONIC SUPPLEMENTARY MATERIALS

Decision-Rule Architecture in Fungal Biomarker-Guided Antifungal Stewardship for Critically Ill Adults: A Systematic Review and *Candida*-Focused Randomised Meta-Analysis

Giuseppe Neri[1]*; Giuseppe Mazza[1]*; Jessica Ielapi[1]; Alessandro Russo[1]; Francesca Serapide[1], Helenia Mastrangelo[2]; Aldo Mesiti[1]; Zaninni Caroleo[3]; Stefano Fresilli[3]; Andrea Bruni[3]; Simona Gigliotti[2]; Angela Quirino[2]; Giovanni Matera[2]; Federico Longhini[1]†; Eugenio Garofalo[1]†

*G.N. and G.M. contributedequally to this work.

† F.L. and E.G. contributed equally to senior supervision of this work.

[1]Department of Medical and Surgical Sciences, Magna Graecia University, Catanzaro, Italy;

[2]Department of Health Sciences, "Renato Dulbecco" University Hospital, 88100 Catanzaro, Italy

[3]Department of Pharmacy, Health and Nutritional Sciences, University of Calabria, 87036 Rende, Italy

Corresponding author:

Simona Gigliotti, PhD

Unit of Clinical Microbiology, “R. Dulbecco” University Hospital,

Department of Health Sciences,

“Magna Graecia” University, Viale Europa, 88100, Catanzaro, Italy

E-mail: s.gigliotti@unicz.it

Tel: +393400805929

EXTENDED REVIEW METHODS AND STATISTICAL ANALYSIS.

The main manuscript presents a condensed Methods section. This appendix provides the full operational methods, including eligibility definitions, search and screening procedures, extraction and appraisal methods, the complete quantitative analysis plan, and the structured narrative-synthesis framework.

Review design, registration, and scope

This systematic review, randomised trial meta-analysis, and structured narrative synthesis was conducted and reported according to PRISMA 2020 and PRISMA-S; the protocol was prospectively registered in PROSPERO (CRD420261432481) on 25 June 2026 [1-2]. The registered record prespecified separate interpretation of initiation/pre-emptive and exposure-reduction strategies. Additional analyses beyond the registered plan are identified explicitly as post hoc in the relevant sections below.

The review evaluated fungal biomarker-guided and biomarker-informed antifungal stewardship strategies in critically ill adults. Because the clinical meaning of a fungal biomarker depends on the therapeutic decision it supports, the synthesis was structured by study design, fungal target, biomarker type, and algorithmic intent rather than by biomarker class alone.

Ethics approval was not required because this systematic review analysed published aggregate data and involved no new contact with participants or access to identifiable information.

Eligibility criteria and PICO framework

Eligible studies included critically ill adults admitted to an intensive care unit or managed in an ICU-relevant setting. This population encompassed patients with sepsis or septic shock, multiorgan failure, severe respiratory failure, intra-abdominal infection, COVID-19-associated critical illness, immunocompromised critical illness, or other clinical syndromes associated with suspected or confirmed invasive fungal disease.

The intervention of interest was any antifungal management strategy guided or informed by a fungal biomarker. Eligible tests included β-(1→3)-D-glucan, Candida mannan or anti-mannan antibodies, T2Candida/T2MR, galactomannan, Aspergillus or Pneumocystis PCR, Mucorales or panfungal PCR, cryptococcal antigen, metagenomic next-generation sequencing, and other prespecified pathogen-specific diagnostic tests identified through the search. To be eligible, however, the test result had to be explicitly linked to an antifungal management decision, such as treatment initiation, withholding, continuation, discontinuation, or modification.

Eligible comparators included standard care, culture-based management, clinician-directed management, empirical or pre-emptive treatment strategies, historical or contemporaneous control groups, and alternative diagnostic-management pathways.

Outcomes of interest included all-cause mortality and measures of systemic antifungal exposure, including treatment initiation, withholding, interruption, discontinuation, de-escalation, and duration. We also considered proven or probable invasive fungal infection, candidemia, breakthrough infection, the need for treatment restart or rescue therapy, adverse events, diagnostic performance, implementation outcomes, and healthcare resource use.

Entry into the intervention synthesis required a fungal test to be explicitly linked to an observed or prespecified antifungal action. Rule-in was defined as a result-contingent increase in treatment initiation or acceleration, whereas rule-out was defined as a result-contingent reduction in continuation of already-started therapy.

A clinically defined exposure-reduction subgroup comprised randomised trials in which the biomarker was used primarily to reduce antifungal exposure through withholding, interruption, early discontinuation, or early termination of empirical therapy. This subgroup was analysed separately because initiation or pre-emptive algorithms and exposure-reduction algorithms address different clinical decisions and may influence antifungal exposure in opposite directions.

Non-randomised studies were eligible for structured narrative synthesis when a fungal biomarker, rapid molecular assay, or diagnostic pathway was explicitly linked to antifungal initiation,

withholding, discontinuation, de-escalation, escalation, or diagnostic-therapeutic stewardship. A secondary contextual domain also retained blinded or decision-analytic reports when they evaluated an explicit treatment-relevant rule against an active antifungal decision, such as discontinuation of empirical therapy. These reports could inform post-test probability, potential gate yield, or pathway design, but not treatment effect or clinical safety. Pure diagnostic-accuracy studies without an explicit antifungal action or treatment-relevant decision question were excluded. Linked diagnostic reports from included randomised populations were retained only for assay behaviour, thresholds, and plausibility, without double-counting participants.

Studies were excluded if they were conducted exclusively in paediatric or neonatal populations, did not include critically ill adults or an ICU-relevant population, lacked a fungal biomarker-informed antifungal management component, were case reports or small case series without a defined management strategy, were reviews, editorials, or commentaries, or were diagnostic-only studies not linked to antifungal decision-making.

Information sources and search strategy

MEDLINE/PubMed and Scopus were searched from inception on 25 June 2026 and updated through 24 July 2026 using the same Boolean concepts. CENTRAL was searched on 28 June and updated on 24 July 2026. ClinicalTrials.gov and WHO ICTRP were searched on 29 June 2026; citation searching, known-trial checks and PROSPERO overlap checks were completed before final synthesis.

The updated searches used the same Boolean concepts as the original searches. Records retrieved during the update were deduplicated within the updated set and reconciled against the original screening master. Records not previously captured underwent title-and-abstract screening and, where appropriate, full-text assessment according to the same predefined eligibility criteria. Complete database-specific strategies, search dates, deduplication procedures, and reconciliation methods are reported in Supplementary Table S1.

Search strategies combined controlled vocabulary and free-text terms for critical illness, intensive care, sepsis, antifungal therapy, fungal pathogens, fungal biomarkers, rapid fungal diagnostics, and stewardship-related therapeutic decision-making. The search was intentionally fungal-wide and was structured to capture three complementary evidence domains: *Candida*-focused randomised biomarker-guided therapeutic evidence, *Candida*-focused observational or pathway-based stewardship evidence, and non-*Candida* ICU biomarker-informed diagnostic-management evidence.

Search terms included *Candida*, candidiasis, candidemia, *Aspergillus*, aspergillosis, *Pneumocystis*, cryptococcosis, mucormycosis, BDG, mannan, anti-mannan, galactomannan, T2Candida, fungal PCR, mNGS, empirical therapy, pre-emptive therapy, antifungal initiation, withholding, interruption, discontinuation, de-escalation, algorithm-based care, and antifungal stewardship. No restriction was applied by fungal pathogen at the search level. No language or date restrictions were applied to the original searches; date/year limits were used only to operationalise the update where supported by the interface. Trial registries were searched to identify completed, ongoing, terminated, or linked studies; records without evaluable outcome reports were not treated as completed intervention evidence.

PubMed and Scopus records were deduplicated across the bibliographic searches before screening. CENTRAL and trial-register entries were screened as source-level records and linked to corresponding reports and independent studies during eligibility assessment. Reference lists of included reports and relevant reviews were checked to identify additional eligible reports.

Study selection, evidence categories, and overlap rules

Records were screened at title and abstract level, or at register-record level for trial registries, against predefined eligibility criteria. Two reviewers (G.N. and G.M.) independently screened records and assessed potentially eligible full-text reports. Disagreements were resolved by discussion and, when necessary, by consultation with a senior third reviewer (E.G.).

Reports proceeded to full-text or report-level assessment when they appeared eligible or when eligibility could not be determined from title, abstract, or registry entry alone. Full-text reports were assessed for population, study design, fungal target, biomarker or diagnostic strategy, antifungal management component, comparator structure, outcome availability, and overlap with other reports. Reasons for exclusion at full-text or report level were recorded using predefined categories.

After eligibility adjudication, each included report was assigned to a predefined evidence category according to its role in synthesis: core *Candida* biomarker-guided randomised evidence, separate mixed-fungal randomised management evidence, *Candida*-focused non-randomised or pathway evidence, non-*Candida* or mixed-fungal diagnostic-management evidence, or linked diagnostic-performance/decision-analytic evidence. Pure diagnostic-only reports without an explicit treatment-relevant decision did not enter the intervention synthesis. This category-based approach prevented inappropriate pooling across randomised trials, observational pathways, diagnostic-performance reports, and mixed-fungal evidence.

Overlapping reports were handled conservatively. When multiple reports derived from the same trial or study population, the primary clinical report was prioritised for clinical and meta-analytic outcomes. Linked reports were retained only when they contributed unique diagnostic, mechanistic, threshold, implementation, or contextual information. For quantitative analyses, the unit of analysis was the independent randomised study population, and participants were counted only once per outcome.

Data extraction and outcomes

Two reviewers (G.N. and G.M.) independently extracted and cross-checked data using a structured extraction framework. Disagreements or ambiguous extractions were resolved by discussion and, when necessary, by senior adjudication (E.G.).

Extracted domains included study design, country, setting, population, inclusion and exclusion criteria, fungal target, biomarker or diagnostic test, cutoff or positivity rule, timing and frequency of testing, algorithmic intent, antifungal decision supported by the biomarker, comparator, follow-up period, and reported outcomes. For randomised trials, additional fields included intervention and control denominators, event counts, continuous outcome measures, outcome time points, and intervention-control direction. For non-randomised, pathway-based, and diagnostic-management studies, additional fields included antifungal-exposure outcomes, safety outcomes, diagnostic-performance measures, implementation outcomes, effect estimates, confidence intervals, AUROC values, sensitivity, specificity, predictive values, and reported limitations. The prespecified extraction framework is provided in Supplementary Table S2. No unreported outcome values were imputed; missing or unclear items were recorded as not reported and did not contribute to quantitative synthesis.

The primary clinical outcome for the randomised synthesis was all-cause mortality at 28 or 30 days. The primary stewardship outcome was antifungal exposure, including systemic antifungal use, early antifungal initiation, treatment duration, early discontinuation, interruption, antifungal-free days, days of therapy, defined daily doses, or the proportion of patients receiving systemic antifungal therapy. Because exposure outcomes differed substantially in definition, timing, and scale, they were synthesised according to algorithmic intent rather than pooled across incompatible continuous endpoints.

Key safety outcomes included proven or probable invasive *Candida* infection, candidemia, breakthrough invasive fungal infection, antifungal restart, adverse events, serious adverse events, ICU mortality, hospital mortality, and other reported clinical safety outcomes. For the pooled policy-safety endpoint, only invasive *Candida* events first diagnosed after randomisation or explicitly reported as subsequent/follow-up were counted; infections present at enrolment were excluded when reports separated them. Diagnostic outcomes included sensitivity, specificity, positive and negative predictive values, AUROC, cutoff performance, *P* values for biomarker

comparisons, and decision-curve or net-benefit analyses. Implementation outcomes included feasibility, turnaround time, clinician adherence, clinician override, antifungal appropriateness, and resource use.

Risk of bias and certainty assessment

Risk of bias was assessed independently by two reviewers (G.N. and G.M.) using design-specific methods. Randomised trials were assessed with RoB 2 [3]. Comparative non-randomised intervention-effect estimates were assessed with ROBINS-I [4]; reports without a defensible causal comparator received a structured design-limitations appraisal rather than a pseudo-summary score. Diagnostic-accuracy estimates and linked ancillary reports were assessed at estimate level with QUADAS-3 version 1.2 [5]. Disagreements were resolved by discussion and, when necessary, by senior adjudication (E.G.).

Assessments were interpreted according to each report's role in the synthesis. Randomised trials informed treatment-effect interpretation. Non-randomised pathway studies informed feasibility, implementation, and hypothesis-generating stewardship signals. Diagnostic-performance studies informed biomarker behaviour, thresholds, and diagnostic plausibility, not treatment effect. Certainty was assessed with GRADE for decision-specific randomised outcomes: mortality and policy-level fungal safety under exposure-reduction strategies; antifungal exposure reduction; CandiSep mortality and systemic treatment receipt under initiation/pre-emptive strategies; and mortality in the separate ACLF comparison [6-7]. Two reviewers developed outcome-level judgments independently and resolved disagreements with senior adjudication. No unitary GRADE certainty rating was assigned to the descriptive 28/30-day mortality aggregation across opposing rule directions because it did not represent a single clinically coherent intervention effect.

Statistical analysis

For dichotomous outcomes, crude risk ratios (RRs) with 95% confidence intervals (CIs) were calculated from the extracted event counts. Quantitative synthesis was undertaken when at least two informative randomised comparisons were available for outcomes considered sufficiently compatible in terms of study design, patient population, algorithmic intent, endpoint definition, measurement scale, and follow-up time point. The independent randomised study population, rather than the individual report, was used as the unit of analysis.

Analyses were stratified according to the therapeutic direction of the biomarker-linked rule. Mortality under exposure-reduction strategies was treated as the principal decision-specific safety analysis for rule-out assignment, whereas mortality in CandiSep was analysed separately as a rule-in estimate. A descriptive aggregation of 28/30-day mortality across opposing rule directions was calculated for transparency but was not interpreted as a unitary treatment effect and was not assigned an overall GRADE certainty rating.

The primary quantitative synthesis was restricted to the core Candida biomarker-guided randomised trials. Observational studies, pathway studies, before-after studies, diagnostic-performance studies, and non-Candida or mixed-fungal studies were not pooled with the core randomised evidence because of differences in patient selection, fungal target, treatment indication, comparator structure, susceptibility to confounding, outcome definition, and algorithmic intent. The randomised mixed-fungal ACLF trial was analysed separately because it evaluated immediate empirical treatment against a bundled diagnostic- and biomarker-informed pre-emptive pathway in a clinically distinct, high-acuity population.

Risk ratios were pooled on the logarithmic scale, with study-level standard errors calculated from 2×2 event data. For inverse-variance analyses, a continuity correction of 0.5 was applied to all four cells of a study when one treatment arm contained no events. Random-effects meta-analysis was prespecified in PROSPERO, although the registration did not specify a between-study variance estimator. Before quantitative pooling and inspection of the pooled results, inverse-variance random-effects analysis using the DerSimonian-Laird estimator was selected as the

primary method [8]. Statistical heterogeneity was assessed using Cochran's Q, τ^2, and I^2 and was interpreted cautiously because all pooled analyses included only a small number of randomised comparisons.

Because the quantitative syntheses included only two to four studies, several alternative methods were used to assess the robustness of the primary estimates. These included restricted maximum likelihood estimation of τ^2 with Wald intervals, modified Hartung-Knapp intervals incorporating a variance safeguard, and Mantel-Haenszel risk ratios [9-11]. When τ^2 was estimated as zero, the REML/Wald analysis served as the principal alternative random-effects analysis. Modified Hartung-Knapp intervals were treated as conservative small-sample sensitivity analyses and were not interpreted mechanically when only two studies were available. Mantel-Haenszel analyses used uncorrected event counts, and the fungal-safety outcome was additionally examined using uncorrected risk differences. These analyses were included because inverse-variance DerSimonian-Laird estimates with continuity correction may be unstable when both the number of studies and the number of events are small; continuity corrections can materially alter sparse-event estimates and may attenuate them toward the null in some configurations.

Outcomes supported by a single randomised comparison, or measured using clinically non-equivalent definitions or scales, were synthesised descriptively. Continuous antifungal-exposure outcomes (including treatment duration, defined daily doses, antifungal-free survival, and early discontinuation) were not combined using a standardised mean difference because they represented different clinical constructs and were measured over different time frames.

Prespecified decision-specific analyses included fixed-time mortality, mortality under exposure-reduction strategies, and invasive Candida events diagnosed after randomisation under rule-out assignment. For the latter endpoint, only infections first diagnosed after randomisation or explicitly reported as subsequent or follow-up events were included; infections already present at enrolment were excluded when the original reports allowed them to be distinguished. This analysis estimated the effect of assignment to a biomarker-guided stopping policy and was not

interpreted as a per-protocol estimate of safety among patients in whom treatment was actually discontinued.

The pooled analysis of systemic antifungal receipt under rule-in assignment was exploratory. The descriptive mortality aggregation across opposing rule directions, alternative τ^2 estimators and interval methods, Mantel-Haenszel and risk-difference analyses, leave-one-out analyses, the proven-only Rouzé analysis, and missing-outcome scenarios, together with the low-activation Erb exclusion, were conducted post hoc as robustness analyses. Trials were selected for pooling according to clinical and methodological compatibility rather than according to whether the intervention produced an observed reduction in antifungal exposure.

Forest plots were generated for the core randomised evidence and combined in a three-panel figure preserving the direction of the treatment rule: systemic antifungal receipt under rule-in assignment; mortality under rule-out assignment; and post-randomisation invasive *Candida* events under rule-out assignment. *CandiSep* mortality remained a separate single-study rule-in estimate reported in text and tables rather than as a forest panel. Two-sided P values were calculated for the primary inverse-variance pooled analyses and reported together with RRs and 95% CIs. Interpretation prioritised effect estimates, absolute effects, confidence intervals, and model-dependent uncertainty rather than statistical significance alone.

Detailed formulas, endpoint definitions, pooling criteria, continuity-correction rules, subgroup definitions, estimator specifications, small-sample and sparse-event sensitivity analyses, direction-of-effect rules, and criteria for narrative-only synthesis are provided in Supplementary Table S3.

Meta-analyses were performed using Review Manager (RevMan), version 5.4.1 (The Cochrane Collaboration), which was also used to generate the forest plots. Additional statistical calculations and sensitivity analyses not implemented in RevMan were performed using Python version 3.13.5, NumPy version 2.3.5, and SciPy version 1.17.0. Formal assessment of funnel-plot asymmetry or small-study effects was not undertaken because no pooled outcome included ten or more randomised comparisons. Formal assessment of funnel-plot asymmetry or small-study

effects was not undertaken because no pooled outcome included ten or more randomised comparisons.

Structured narrative synthesis

The narrative synthesis followed SWiM reporting principles [12]. Studies were grouped a priori by design, fungal target, algorithmic intent, and outcome domain. Exact study-level effects and implementation measures were tabulated; statistical significance was not used for vote counting. When pooling was inappropriate, we compared the full decision architecture: rule direction (initiation, withholding, discontinuation, or de-escalation), trigger logic (single versus serial results with AND/OR combinations), threshold, sampling and result timing, baseline risk, the proportion in whom the algorithm became actionable, adherence and clinician override, and rescue or restart rules. The mixed-fungal ACLF trial was synthesised separately because its high-risk population and bundled confirmation-dependent pathway were incompatible with the *Candida* comparisons.

Narrative conclusions were restricted according to design and risk of bias. Non-randomised studies informed feasibility, implementation, post-test probability, and hypothesis-generating pathway design; they were not treated as independent causal estimates. Direction-of-effect language was used only when outcomes represented the same clinical construct, and limitations of each grouping are reported with the synthesis.

REFERENCES

1. Page, M.J.; McKenzie, J.E.; Bossuyt, P.M.; Boutron, I.; Hoffmann, T.C.; Mulrow, C.D.; Shamseer, L.; Tetzlaff, J.M.; Akl, E.A.; Brennan, S.E.*, et al.* The prisma 2020 statement: An updated guideline for reporting systematic reviews. *BMJ* **2021**, *372*, n71.
2. Rethlefsen, M.L.; Kirtley, S.; Waffenschmidt, S.; Ayala, A.P.; Moher, D.; Page, M.J.; Koffel, J.B. Prisma-s: An extension to the prisma statement for reporting literature searches in systematic reviews. *Syst Rev* **2021**, *10*, 39.
3. Sterne, J.A.C.; Savovic, J.; Page, M.J.; Elbers, R.G.; Blencowe, N.S.; Boutron, I.; Cates, C.J.; Cheng, H.Y.; Corbett, M.S.; Eldridge, S.M.*, et al.* Rob 2: A revised tool for assessing risk of bias in randomised trials. *BMJ* **2019**, *366*, l4898.
4. Sterne, J.A.; Hernan, M.A.; Reeves, B.C.; Savovic, J.; Berkman, N.D.; Viswanathan, M.; Henry, D.; Altman, D.G.; Ansari, M.T.; Boutron, I.*, et al.* Robins-i: A tool for assessing risk of bias in non-randomised studies of interventions. *BMJ* **2016**, *355*, i4919.
5. Whiting, P.F.; Tomlinson, E.; Rutjes, A.W.S.; Davenport, C.F.; Yang, B.; Westwood, M.E.; Takwoingi, Y.; Reitsma, J.B.; Hyde, C.; Bossuyt, P.M.M.*, et al.* Quadas-3: A revised tool for the quality assessment of diagnostic test accuracy studies. *Ann Intern Med* **2026**, *179*, 548-555.
6. Balshem, H.; Helfand, M.; Schunemann, H.J.; Oxman, A.D.; Kunz, R.; Brozek, J.; Vist, G.E.; Falck-Ytter, Y.; Meerpohl, J.; Norris, S.*, et al.* Grade guidelines: 3. Rating the quality of evidence. *J Clin Epidemiol* **2011**, *64*, 401-406.
7. Guyatt, G.; Oxman, A.D.; Akl, E.A.; Kunz, R.; Vist, G.; Brozek, J.; Norris, S.; Falck-Ytter, Y.; Glasziou, P.; DeBeer, H.*, et al.* Grade guidelines: 1. Introduction-grade evidence profiles and summary of findings tables. *J Clin Epidemiol* **2011**, *64*, 383-394.
8. DerSimonian, R.; Laird, N. Meta-analysis in clinical trials. *Control Clin Trials* **1986**, *7*, 177-188.

9. Rover, C.; Knapp, G.; Friede, T. Hartung-knapp-sidik-jonkman approach and its modification for random-effects meta-analysis with few studies. *BMC Med Res Methodol* **2015**, *15*, 99.

10. IntHout, J.; Ioannidis, J.P.; Borm, G.F. The hartung-knapp-sidik-jonkman method for random effects meta-analysis is straightforward and considerably outperforms the standard dersimonian-laird method. *BMC Med Res Methodol* **2014**, *14*, 25.

11. Sweeting, M.J.; Sutton, A.J.; Lambert, P.C. What to add to nothing? Use and avoidance of continuity corrections in meta-analysis of sparse data. *Stat Med* **2004**, *23*, 1351-1375.

12. Campbell, M.; McKenzie, J.E.; Sowden, A.; Katikireddi, S.V.; Brennan, S.E.; Ellis, S.; Hartmann-Boyce, J.; Ryan, R.; Shepperd, S.; Thomas, J*., et al.* Synthesis without meta-analysis (swim) in systematic reviews: Reporting guideline. *BMJ* **2020**, *368*, l6890.

TABLE S1. SEARCH STRATEGIES, SOURCE ACCOUNTING, AND PRISMA 2020 RECONCILIATION

This table reports all executed database and trial-register searches, exact strategies, raw source counts, deduplication decisions, record-level update reconciliation and report-level accounting. The original PubMed and Scopus searches were completed on 25 June 2026, CENTRAL on 28 June 2026, and ClinicalTrials.gov, WHO ICTRP and PROSPERO checks on 29 June 2026. PubMed, Scopus and CENTRAL were updated through 24 July 2026 using the same search concepts. The update produced 31 raw hits, 26 update-unique records, 22 overlaps with the original master and four genuinely new records. No language or date restrictions were applied to the original searches; date/year limits were used only to operationalise the update where supported by the interface. Search A was a verification search and was not imported as an additional screening stream.

The PRISMA identification denominator uses raw outputs from the original counted Search B and Search C streams, CENTRAL, ClinicalTrials.gov and WHO ICTRP, plus every raw hit returned by the 24 July PubMed/Scopus/CENTRAL update. Duplicate occurrences were then removed within the original streams, within the update and between the update and the original master before calculating the final unique screening denominator. PROSPERO was used only to assess review overlap and was not counted as a primary-study source.

Panel A. Documented search sources and raw outputs

Source	Search role	Date	Records / hits	PRISMA and synthesis handling
MEDLINE/PubMed	Search A: RCT-focused Candida verification	25 Jun 2026; verified 24 Jul 2026	51 (original verification run)	Verification search; overlapped with Search B and was not imported or counted as an additional PRISMA stream.
Scopus	Search A: RCT-focused Candida verification	25 Jun 2026; verified 24 Jul 2026	93 (original verification run)	Verification search; overlapped with Search B and was not imported or counted as an additional PRISMA stream.
MEDLINE/PubMed	Search B: expanded Candida stewardship/pathway evidence	25 Jun 2026	150	Original raw counted output; combined with 256 Scopus records and deduplicated to a 281-record Candida-focused master.
Scopus	Search B: expanded Candida stewardship/pathway evidence	25 Jun 2026	256	Original raw counted output; combined with 150 PubMed records and deduplicated to a 281-record Candida-focused master.
MEDLINE/PubMed	Search C: targeted non-Candida/mixed fungal check	25 Jun 2026	79	Original raw counted output; combined with 174 Scopus records and deduplicated to a 184-record Search C master.
Scopus	Search C: targeted non-Candida/mixed fungal check	25 Jun 2026	174	Original raw counted output; combined with 79 PubMed records and deduplicated to a 184-record Search C master.
Cochrane CENTRAL	Trial-focused fungal biomarker stewardship search	28 Jun 2026	29 trial records	Original counted trial records. The interface displayed 33 total Cochrane records; only the 29 CENTRAL trial records entered primary-study accounting.

Source	Search role	Date	Records / hits	PRISMA and synthesis handling
ClinicalTrials.gov	Formal trial-register search	29 Jun 2026	16	Original 16-row registry ledger screened individually.
WHO ICTRP	Formal broad trial-register search	29 Jun 2026	3	Original three registry records screened individually; the separate named-trial check returned no additional record.
MEDLINE/PubMed	24 July update: Search B	24 Jul 2026	3	Raw update hits; counted before update deduplication and prior-master comparison.
Scopus	24 July update: Search B	24 Jul 2026	26	Raw 2026 export screened against the original master; counted before update deduplication and prior-master comparison.
MEDLINE/PubMed	24 July update: Search C	24 Jul 2026	1	Raw update hit; duplicated the Rivas-Pinedo review already returned by the other update streams.
Scopus	24 July update: Search C	24 Jul 2026	1	Raw update hit; duplicated the Rivas-Pinedo review already returned by the other update streams.
Cochrane CENTRAL	24 July update	24 Jul 2026	0	No new CENTRAL trial record entered the update flow.
PROSPERO	Review overlap/protocol checks	29 Jun 2026	0 + 1	Review-overlap checks only; not counted as primary-study records.

Panel B. Audited PRISMA 2020 record reconciliation

PRISMA component	Count	Derivation / audit basis
Records identified from databases and registers	738	707 original raw records + 31 raw update hits (PubMed 4, Scopus 27, CENTRAL 0).
Duplicate or previously screened records removed	263	236 original duplicates + 5 duplicate occurrences within the update + 22 update records already present in the original master.
Unique records screened	475	738 - 263.
Records excluded before report retrieval	444	475 - 31 reports remaining after title/abstract or registry-record screening.
Reports sought for retrieval	31	Previous 29-report set + newly identified Albanell-Fernández + Abi Kheir, already present in the original screening master and advanced to full-text assessment during reconciliation.
Reports not retrieved	0	All 31 reports were retrieved or otherwise available for report-level eligibility assessment.
Reports assessed for eligibility	31	All reports remaining after title/abstract screening were assessed at full-text/report level.
Reports excluded after eligibility assessment	10	Reasons are reported individually in Supplementary Table S4; three antifungal-strategy RCT reports were excluded because biomarkers were ancillary and did not define the allocated intervention.
Reports included in the review	21	31 - 10.
Independent studies included in the review	19	Two included reports were linked analyses: Standl to CandiSep and Giacobbe to Posteraro.

Panel C. Cross-source duplicate blocks verified before screening

Duplicate/overlap block	Records removed	Verification basis
Within original Search B PubMed/Scopus	125	406 raw records -> 281-record master.
Within original Search C PubMed/Scopus	69	253 raw records -> 184-record master.
Between original Search B and Search C masters	26	281 + 184 -> 439-row bibliographic screening master.
CENTRAL duplicated in bibliographic master	9	Original documented record-level overlaps.
ClinicalTrials.gov duplicated in CENTRAL	6	Original documented registry/database overlaps.
WHO ICTRP duplicated in ClinicalTrials.gov	1	NCT00672841.
Duplicate occurrences within the July update	5	31 raw update hits -> 26 update-unique records.
July update records already in the original master	22	Record-level DOI/title comparison of the 26 update-unique records against the original screening master.
Total duplicate or previously screened records removed	263	125 + 69 + 26 + 9 + 6 + 1 + 5 + 22.

Panel D. Included-report and independent-study accounting

Evidence block	Included reports	Independent studies	Counting rule
Core *Candida* biomarker-guided randomised trials	5	5	Independent randomised study populations.
Separate mixed-fungal ACLF randomised trial	1	1	Independent study; synthesised separately from the *Candida* meta-analysis.
Primary narrative/pathway studies	13	13	Independent non-randomised or implementation/diagnostic-management populations. Albanell-Fernández was newly identified in the July update; Abi Kheir was already present in the original master and was included after full-text assessment during reconciliation.
Giacobbe 2019 DOOR report	1	0	Post hoc analysis of the Posteraro 2016 prospective observational cohort (198 original patients; 186 included in the DOOR analysis).
Standl 2026 diagnostic report	1	0	Secondary diagnostic endpoint report from the CandiSep randomised population.
Total	21	19	Reports are counted separately; linked reports are counted once at study level.

LAMBDA was eligible for narrative synthesis because continuation after day 1 was prespecified as BDG-dependent. It contributes feasibility/tolerability evidence only; the single-arm design provides no comparative treatment or safety effect.

Panel E. Exact search strategies

MEDLINE/PubMed - Search A: RCT-focused *Candida* core

("critical care"[tiab] OR "critically ill"[tiab] OR ICU[tiab] OR "intensive care"[tiab] OR sepsis[tiab] OR "septic shock"[tiab] OR "Critical Care"[Mesh] OR "Intensive Care Units"[Mesh] OR "Sepsis"[Mesh] OR "Shock, Septic"[Mesh]) AND (*Candida*[tiab] OR candidiasis[tiab] OR candidemia[tiab] OR candidaemia[tiab] OR "invasive candidiasis"[tiab] OR "invasive fungal infection"[tiab] OR antifungal*[tiab] OR "Antifungal Agents"[Mesh]) AND ("beta-D-glucan"[tiab] OR "beta D glucan"[tiab] OR "1,3-beta-D-glucan"[tiab] OR BDG[tiab] OR Fungitell[tiab] OR mannan[tiab] OR "anti-mannan"[tiab] OR antimannan[tiab] OR "fungal biomarker"[tiab] OR "fungal biomarkers"[tiab] OR CandiSep[tiab]) AND (random*[tiab] OR randomized controlled trial[pt] OR controlled clinical trial[pt] OR trial[tiab] OR placebo[tiab]) NOT (animals[mh] NOT humans[mh])

Scopus - Search A: RCT-focused *Candida* core

TITLE-ABS-KEY (("critical care" OR "critically ill" OR ICU OR "intensive care" OR sepsis OR "septic shock") AND (*Candida* OR candidiasis OR candidemia OR candidaemia OR "invasive candidiasis" OR "invasive fungal infection" OR antifungal OR antifungals OR echinocandin OR echinocandins OR fluconazole OR micafungin OR caspofungin OR anidulafungin) AND ("beta-D-glucan" OR "beta D glucan" OR "1,3-beta-D-glucan" OR BDG OR Fungitell OR mannan OR "anti-mannan" OR antimannan OR "fungal biomarker" OR "fungal biomarkers" OR CandiSep) AND (random OR randomized OR randomised OR trial OR placebo OR "controlled trial"))

MEDLINE/PubMed - Search B: expanded *Candida* stewardship/observational

("critical care"[tiab] OR "critically ill"[tiab] OR "critical illness"[tiab] OR ICU[tiab] OR "intensive care"[tiab] OR "intensive care unit"[tiab] OR "intensive care units"[tiab] OR sepsis[tiab] OR "septic shock"[tiab] OR "surgical ICU"[tiab] OR "Critical Care"[Mesh] OR "Intensive Care Units"[Mesh] OR "Sepsis"[Mesh] OR "Shock, Septic"[Mesh]) AND (*Candida*[tiab] OR candidiasis[tiab] OR candidemia[tiab] OR candidaemia[tiab] OR "invasive candidiasis"[tiab] OR "intra-abdominal candidiasis"[tiab] OR "deep-seated candidiasis"[tiab] OR "*Candida* peritonitis"[tiab] OR "invasive fungal infection"[tiab] OR "invasive yeast infection"[tiab] OR antifungal*[tiab] OR echinocandin*[tiab] OR fluconazole[tiab] OR micafungin[tiab] OR caspofungin[tiab] OR anidulafungin[tiab] OR "Antifungal Agents"[Mesh]) AND ("beta-D-glucan"[tiab] OR "beta D glucan"[tiab] OR "1,3-beta-D-glucan"[tiab] OR "1,3 β-D-glucan"[tiab] OR "β-D-glucan"[tiab] OR "β-glucan"[tiab] OR BDG[tiab] OR

Fungitell[tiab] OR mannan[tiab] OR "anti-mannan"[tiab] OR antimannan[tiab] OR "*Candida* antigen"[tiab] OR CAGTA[tiab] OR T2Candida[tiab] OR "fungal biomarker"[tiab] OR "fungal biomarkers"[tiab] OR CandiSep[tiab]) AND (stewardship[tiab] OR algorithm*[tiab] OR guided[tiab] OR "biomarker-guided"[tiab] OR discontinu*[tiab] OR interrupt*[tiab] OR terminat*[tiab] OR stop*[tiab] OR cessation[tiab] OR withdraw*[tiab] OR withholding[tiab] OR "de-escalation"[tiab] OR deescalation[tiab] OR empirical[tiab] OR empiric[tiab] OR preemptive[tiab] OR "pre-emptive"[tiab] OR consumption[tiab] OR exposure[tiab] OR duration[tiab]) NOT (animals[mh] NOT humans[mh])

Scopus - Search B: expanded *Candida* stewardship/observational

TITLE-ABS-KEY (("critical care" OR "critically ill" OR "critical illness" OR ICU OR "intensive care" OR "intensive care unit" OR "intensive care units" OR sepsis OR "septic shock" OR "surgical ICU") AND (*Candida* OR candidiasis OR candidemia OR candidaemia OR "invasive candidiasis" OR "intra-abdominal candidiasis" OR "deep-seated candidiasis" OR "*Candida* peritonitis" OR "invasive fungal infection" OR "invasive yeast infection" OR antifungal* OR echinocandin* OR fluconazole OR micafungin OR caspofungin OR anidulafungin) AND ("beta-D-glucan" OR "beta D glucan" OR "1,3-beta-D-glucan" OR "1,3 β-D-glucan" OR "β-D-glucan" OR "β-glucan" OR BDG OR Fungitell OR mannan OR "anti-mannan" OR antimannan OR "*Candida* antigen" OR CAGTA OR T2Candida OR "fungal biomarker" OR "fungal biomarkers" OR CandiSep) AND (stewardship OR algorithm* OR guided OR "biomarker-guided" OR discontinu* OR interrupt* OR terminat* OR stop* OR cessation OR withdraw* OR withholding OR "de-escalation" OR deescalation OR empirical OR empiric OR preemptive OR "pre-emptive" OR consumption OR exposure OR duration))

PubMed/MEDLINE - Search C: targeted non-*Candida* fungal-wide check

("critical care"[tiab] OR "critically ill"[tiab] OR "critical illness"[tiab] OR ICU[tiab] OR "intensive care"[tiab] OR "intensive care unit"[tiab] OR sepsis[tiab] OR "septic shock"[tiab] OR "acute respiratory failure"[tiab] OR "mechanically ventilated"[tiab] OR "Critical Care"[Mesh] OR "Intensive Care Units"[Mesh]) AND (*Aspergillus*[tiab] OR aspergillosis[tiab] OR "invasive aspergillosis"[tiab] OR "invasive pulmonary aspergillosis"[tiab] OR IPA[tiab] OR CAPA[tiab] OR "COVID-19-associated pulmonary aspergillosis"[tiab] OR *Pneumocystis*[tiab] OR "*Pneumocystis jirovecii*"[tiab] OR PJP[tiab] OR PCP[tiab] OR cryptococ*[tiab] OR "cryptococcal meningitis"[tiab] OR mucormycosis[tiab] OR Mucorales[tiab] OR mucor*[tiab] OR histoplasmosis[tiab] OR blastomycosis[tiab] OR coccidioidomycosis[tiab] OR "invasive mould infection"[tiab] OR "invasive mold infection"[tiab] OR "invasive fungal disease"[tiab]) AND (galactomannan[tiab] OR "galactomannan

antigen"[tiab] OR "*Aspergillus* antigen"[tiab] OR "BAL galactomannan"[tiab] OR "bronchoalveolar lavage galactomannan"[tiab] OR "*Aspergillus* PCR"[tiab] OR "*Aspergillus* polymerase chain reaction"[tiab] OR "lateral flow"[tiab] OR LFA[tiab] OR "beta-D-glucan"[tiab] OR "beta D glucan"[tiab] OR BDG[tiab] OR Fungitell[tiab] OR "*Pneumocystis* PCR"[tiab] OR "cryptococcal antigen"[tiab] OR CrAg[tiab] OR "Mucorales PCR"[tiab] OR "panfungal PCR"[tiab] OR "fungal biomarker"[tiab] OR "fungal biomarkers"[tiab] OR metagenomic[tiab] OR mNGS[tiab]) AND (random*[tiab] OR randomized controlled trial[pt] OR controlled clinical trial[pt] OR trial[tiab] OR placebo[tiab] OR stewardship[tiab] OR algorithm*[tiab] OR guided[tiab] OR "biomarker-guided"[tiab] OR "diagnostic-driven"[tiab] OR preemptive[tiab] OR "pre-emptive"[tiab] OR empirical[tiab] OR empiric[tiab] OR discontinu*[tiab] OR withholding[tiab] OR "de-escalation"[tiab] OR deescalation[tiab] OR stop*[tiab]) NOT (*Candida*[tiab] OR candidemia[tiab] OR candidaemia[tiab] OR candidiasis[tiab] OR "invasive candidiasis"[tiab]) NOT (animals[mh] NOT humans[mh])

Scopus - Search C: targeted non-*Candida* fungal-wide check

TITLE-ABS-KEY (("critical care" OR "critically ill" OR "critical illness" OR ICU OR "intensive care" OR "intensive care unit" OR sepsis OR "septic shock" OR "acute respiratory failure" OR "mechanically ventilated") AND (*Aspergillus* OR aspergillosis OR "invasive aspergillosis" OR "invasive pulmonary aspergillosis" OR IPA OR CAPA OR "COVID-19-associated pulmonary aspergillosis" OR *Pneumocystis* OR "*Pneumocystis jirovecii*" OR PJP OR PCP OR cryptococ* OR "cryptococcal meningitis" OR mucormycosis OR Mucorales OR mucor* OR histoplasmosis OR blastomycosis OR coccidioidomycosis OR "invasive mould infection" OR "invasive mold infection" OR "invasive fungal disease") AND (galactomannan OR "galactomannan antigen" OR "*Aspergillus* antigen" OR "BAL galactomannan" OR "bronchoalveolar lavage galactomannan" OR "*Aspergillus* PCR" OR "*Aspergillus* polymerase chain reaction" OR "lateral flow" OR LFA OR "beta-D-glucan" OR "beta D glucan" OR BDG OR Fungitell OR "*Pneumocystis* PCR" OR "cryptococcal antigen" OR CrAg OR "Mucorales PCR" OR "panfungal PCR" OR "fungal biomarker" OR "fungal biomarkers" OR metagenomic OR mNGS) AND (random* OR trial OR placebo OR stewardship OR algorithm* OR guided OR "biomarker-guided" OR "diagnostic-driven" OR preemptive OR "pre-emptive" OR empirical OR empiric OR discontinu* OR withholding OR "de-escalation" OR deescalation OR stop*) AND NOT (*Candida* OR candidemia OR candidaemia OR candidiasis OR "invasive candidiasis"))

Cochrane CENTRAL - trial-focused fungal biomarker stewardship check

("critical care" OR "critically ill" OR ICU OR "intensive care" OR sepsis OR "septic shock") AND (*Candida* OR candidiasis OR candidemia OR candidaemia OR "invasive candidiasis" OR "invasive fungal infection" OR antifungal OR antifungals OR echinocandin OR echinocandins OR fluconazole OR micafungin OR caspofungin OR anidulafungin) AND ("beta-D-glucan" OR "beta D glucan" OR "1,3-beta-D-glucan" OR BDG OR Fungitell OR mannan OR "anti-mannan" OR antimannan OR "fungal biomarker" OR "fungal biomarkers" OR CandiSep)

ClinicalTrials.gov - formal trial-register search

(*Candida* OR candidiasis OR candidemia OR candidaemia OR "invasive candidiasis" OR "invasive fungal infection" OR *Aspergillus* OR aspergillosis OR *Pneumocystis* OR cryptococcus OR mucormycosis) AND ("critical care" OR "critically ill" OR ICU OR "intensive care" OR sepsis OR "septic shock") AND ("beta-D-glucan" OR "beta D glucan" OR BDG OR Fungitell OR mannan OR "anti-mannan" OR antimannan OR galactomannan OR T2Candida OR PCR OR metagenomic OR sequencing OR biomarker) AND (antifungal OR antifungals OR echinocandin OR micafungin OR caspofungin OR anidulafungin OR fluconazole OR voriconazole OR isavuconazole OR amphotericin) AND (stewardship OR guided OR algorithm OR discontinuation OR interruption OR termination OR withholding OR "de-escalation" OR deescalation OR preemptive OR "pre-emptive" OR empirical OR empiric)

WHO ICTRP - formal broad trial-register search

(*Candida* OR candidiasis OR candidemia OR candidaemia OR *Aspergillus* OR aspergillosis OR *Pneumocystis*) AND (ICU OR "intensive care" OR sepsis OR "septic shock") AND ("beta-D-glucan" OR BDG OR mannan OR galactomannan OR T2Candida OR biomarker) AND (antifungal OR echinocandin OR micafungin OR fluconazole)

WHO ICTRP - named-trial registry check

CandiSep OR S-TAFE OR Rouze OR "De Pascale" OR Erb OR Hanson OR EMPIRICUS OR MSG-01 OR Knitsch

PROSPERO - protocol/overlap search

Candida biomarker antifungal stewardship systematic review

PROSPERO - exact CRD overlap check
CRD42022375425

Panel F. Complete database-update strategies and reconciliation

The following strings document the 24 July 2026 database update. PubMed used an explicit publication-date interval. Scopus used the complete 2026 export because day/month filtering is not uniformly available across record types; exported records were compared by DOI/title with the original master. CENTRAL was rerun using the original trial-focused strategy and produced no new trial record.

MEDLINE/PubMed - Search B update, 26 June to 24 July 2026

("critical care"[tiab] OR "critically ill"[tiab] OR "critical illness"[tiab] OR ICU[tiab] OR "intensive care"[tiab] OR "intensive care unit"[tiab] OR "intensive care units"[tiab] OR sepsis[tiab] OR "septic shock"[tiab] OR "surgical ICU"[tiab] OR "Critical Care"[Mesh] OR "Intensive Care Units"[Mesh] OR "Sepsis"[Mesh] OR "Shock, Septic"[Mesh]) AND (Candida[tiab] OR candidiasis[tiab] OR candidemia[tiab] OR candidaemia[tiab] OR "invasive candidiasis"[tiab] OR "intra-abdominal candidiasis"[tiab] OR "deep-seated candidiasis"[tiab] OR "Candida peritonitis"[tiab] OR "invasive fungal infection"[tiab] OR "invasive yeast infection"[tiab] OR antifungal*[tiab] OR echinocandin*[tiab] OR fluconazole[tiab] OR micafungin[tiab] OR caspofungin[tiab] OR anidulafungin[tiab] OR "Antifungal Agents"[Mesh]) AND ("beta-D-glucan"[tiab] OR "beta D glucan"[tiab] OR "1,3-beta-D-glucan"[tiab] OR "1,3 β-D-glucan"[tiab] OR "β-D-glucan"[tiab] OR "β-glucan"[tiab] OR BDG[tiab] OR Fungitell[tiab] OR mannan[tiab] OR "anti-mannan"[tiab] OR antimannan[tiab] OR "Candida antigen"[tiab] OR CAGTA[tiab] OR T2Candida[tiab] OR "fungal biomarker"[tiab] OR "fungal biomarkers"[tiab] OR CandiSep[tiab]) AND (stewardship[tiab] OR algorithm*[tiab] OR guided[tiab] OR "biomarker-guided"[tiab] OR discontinu*[tiab] OR interrupt*[tiab] OR terminat*[tiab] OR stop*[tiab] OR cessation[tiab] OR withdraw*[tiab] OR withholding[tiab] OR "de-escalation"[tiab] OR deescalation[tiab] OR empirical[tiab] OR empiric[tiab] OR preemptive[tiab] OR "pre-emptive"[tiab] OR consumption[tiab] OR exposure[tiab] OR duration[tiab]) NOT (animals[mh] NOT humans[mh]) AND ("2026/06/26"[Date - Publication] : "2026/07/24"[Date - Publication])

Scopus - Search B update, 2026 export reconciled through 24 July 2026

TITLE-ABS-KEY (("critical care" OR "critically ill" OR "critical illness" OR ICU OR "intensive care" OR "intensive care unit" OR "intensive care units" OR sepsis OR "septic shock" OR "surgical ICU") AND (Candida OR candidiasis OR candidemia OR candidaemia OR "invasive candidiasis" OR "intra-abdominal candidiasis" OR "deep-seated candidiasis" OR "Candida peritonitis" OR "invasive fungal infection" OR "invasive yeast infection" OR antifungal* OR echinocandin* OR fluconazole OR micafungin OR caspofungin OR anidulafungin) AND ("beta-D-glucan" OR "beta D glucan" OR "1,3-beta-D-glucan" OR "1,3 β-D-glucan" OR "β-D-glucan" OR "β-glucan" OR BDG OR Fungitell OR mannan OR "anti-mannan" OR antimannan OR "Candida antigen" OR CAGTA OR T2Candida OR "fungal biomarker" OR "fungal biomarkers" OR CandiSep) AND (stewardship OR algorithm* OR guided OR "biomarker-guided" OR discontinu* OR interrupt* OR terminat* OR stop* OR cessation OR withdraw* OR withholding OR "de-escalation" OR deescalation OR empirical OR empiric OR preemptive OR "pre-emptive" OR consumption OR exposure OR duration)) AND PUBYEAR = 2026

MEDLINE/PubMed - Search C update, 26 June to 24 July 2026

("critical care"[tiab] OR "critically ill"[tiab] OR "critical illness"[tiab] OR ICU[tiab] OR "intensive care"[tiab] OR "intensive care unit"[tiab] OR sepsis[tiab] OR "septic shock"[tiab] OR "acute respiratory failure"[tiab] OR "mechanically ventilated"[tiab] OR "Critical Care"[Mesh] OR "Intensive Care Units"[Mesh]) AND (Aspergillus[tiab] OR aspergillosis[tiab] OR "invasive aspergillosis"[tiab] OR "invasive pulmonary aspergillosis"[tiab] OR IPA[tiab] OR CAPA[tiab] OR "COVID-19-associated pulmonary aspergillosis"[tiab] OR Pneumocystis[tiab] OR "Pneumocystis jirovecii"[tiab] OR PJP[tiab] OR PCP[tiab] OR cryptococ*[tiab] OR "cryptococcal meningitis"[tiab] OR mucormycosis[tiab] OR Mucorales[tiab] OR mucor*[tiab] OR histoplasmosis[tiab] OR blastomycosis[tiab] OR coccidioidomycosis[tiab] OR "invasive mould infection"[tiab] OR "invasive mold infection"[tiab] OR "invasive fungal disease"[tiab]) AND (galactomannan[tiab] OR "galactomannan antigen"[tiab] OR "Aspergillus antigen"[tiab] OR "BAL galactomannan"[tiab] OR "bronchoalveolar lavage galactomannan"[tiab] OR "Aspergillus PCR"[tiab] OR "Aspergillus polymerase chain reaction"[tiab] OR "lateral flow"[tiab] OR LFA[tiab] OR "beta-D-glucan"[tiab] OR "beta D glucan"[tiab] OR BDG[tiab] OR Fungitell[tiab] OR "Pneumocystis PCR"[tiab] OR "cryptococcal antigen"[tiab] OR CrAg[tiab] OR "Mucorales PCR"[tiab] OR "panfungal PCR"[tiab] OR "fungal biomarker"[tiab] OR "fungal biomarkers"[tiab] OR metagenomic[tiab] OR mNGS[tiab]) AND (random*[tiab] OR randomized controlled trial[pt] OR controlled clinical trial[pt] OR trial[tiab] OR placebo[tiab] OR stewardship[tiab] OR algorithm*[tiab] OR guided[tiab] OR "biomarker-guided"[tiab] OR "diagnostic-driven"[tiab] OR preemptive[tiab] OR "pre-emptive"[tiab] OR empirical[tiab] OR empiric[tiab] OR discontinu*[tiab] OR withholding[tiab] OR "de-escalation"[tiab] OR deescalation[tiab] OR stop*[tiab]) NOT (Candida[tiab] OR candidemia[tiab] OR candidaemia[tiab]

OR candidiasis[tiab] OR "invasive candidiasis"[tiab]) NOT (animals[mh] NOT humans[mh]) AND ("2026/06/26"[Date - Publication] : "2026/07/24"[Date - Publication])

Scopus - Search C update, 2026 export reconciled through 24 July 2026

TITLE-ABS-KEY (("critical care" OR "critically ill" OR "critical illness" OR ICU OR "intensive care" OR "intensive care unit" OR sepsis OR "septic shock" OR "acute respiratory failure" OR "mechanically ventilated") AND (Aspergillus OR aspergillosis OR "invasive aspergillosis" OR "invasive pulmonary aspergillosis" OR IPA OR CAPA OR "COVID-19-associated pulmonary aspergillosis" OR Pneumocystis OR "Pneumocystis jirovecii" OR PJP OR PCP OR cryptococ* OR "cryptococcal meningitis" OR mucormycosis OR Mucorales OR mucor* OR histoplasmosis OR blastomycosis OR coccidioidomycosis OR "invasive mould infection" OR "invasive mold infection" OR "invasive fungal disease") AND (galactomannan OR "galactomannan antigen" OR "Aspergillus antigen" OR "BAL galactomannan" OR "bronchoalveolar lavage galactomannan" OR "Aspergillus PCR" OR "Aspergillus polymerase chain reaction" OR "lateral flow" OR LFA OR "beta-D-glucan" OR "beta D glucan" OR BDG OR Fungitell OR "Pneumocystis PCR" OR "cryptococcal antigen" OR CrAg OR "Mucorales PCR" OR "panfungal PCR" OR "fungal biomarker" OR "fungal biomarkers" OR metagenomic OR mNGS) AND (random* OR trial OR placebo OR stewardship OR algorithm* OR guided OR "biomarker-guided" OR "diagnostic-driven" OR preemptive OR "pre-emptive" OR empirical OR empiric OR discontinu* OR withholding OR "de-escalation" OR deescalation OR stop*) AND NOT (Candida OR candidemia OR candidaemia OR candidiasis OR "invasive candidiasis")) AND PUBYEAR = 2026

Cochrane CENTRAL - update through 24 July 2026

("critical care" OR "critically ill" OR ICU OR "intensive care" OR sepsis OR "septic shock") AND (Candida OR candidiasis OR candidemia OR candidaemia OR "invasive candidiasis" OR "invasive fungal infection" OR antifungal OR antifungals OR echinocandin OR echinocandins OR fluconazole OR micafungin OR caspofungin OR anidulafungin) AND ("beta-D-glucan" OR "beta D glucan" OR "1,3-beta-D-glucan" OR BDG OR Fungitell OR mannan OR "anti-mannan" OR antimannan OR "fungal biomarker" OR "fungal biomarkers" OR CandiSep)

TABLE S2. DATA-EXTRACTION FRAMEWORK AND STRUCTURED NARRATIVE/DIAGNOSTIC OUTCOME EXTRACTION

This table reports the prespecified extraction domains and report-level outcomes for evidence not pooled in the core *Candida* meta-analysis: one separately synthesised mixed-fungal RCT, thirteen primary narrative studies, and two linked reports. Core *Candida* RCT event counts and pooled estimates are reported in Supplementary Table S8. Reported diagnostic metrics, P values, and non-randomised treatment associations are descriptive and were not interpreted as causal treatment effects.

Cross-trial toxicity, cost, resistance, and ecology were too sparsely reported for synthesis. Hanson reported 15 possibly drug-related events in 10/21 pre-emptively treated participants, none serious. CandiSep reported median antifungal costs of €4451 versus €2800 per patient (P=0.52). No core trial quantified resistance selection or ecological consequences.

Abbreviations: ACLF, acute-on-chronic liver failure; AFT, antifungal therapy; AUC/AUROC, area under the receiver operating characteristic curve; BAL, bronchoalveolar lavage; BDG, β-(1→3)-D-glucan; CAGTA, *Candida albicans* germ-tube antibody; CAPA, COVID-19-associated pulmonary aspergillosis; DDD, defined daily doses; DOOR, desirability of outcome ranking; GMI, galactomannan index; IAC, intra-abdominal candidiasis; IC, invasive candidiasis; ICI, invasive *Candida* infection; ICU, intensive care unit; IFI, invasive fungal infection; IPA, invasive pulmonary aspergillosis; mNGS, metagenomic next-generation sequencing; NPV, negative predictive value; OR, odds ratio; PPV, positive predictive value; RCT, randomised controlled trial; T2MR, T2 magnetic resonance.

Panel A. Data-extraction and synthesis framework

Extraction domain	Variables extracted	Applied to	Purpose	Methodological constraint
Report identification and overlap	Author/year, PMID/DOI, country, design, recruitment period, setting, enrolment source, parent-study identifier, and linked-report status.	All reports	Establish the report-to-study map and prevent double counting.	Multiple reports from the same underlying population were linked and counted once at study level.
Population and clinical setting	Age, ICU/critical-care setting, clinical syndrome, immune status, organ support, sepsis/septic shock, surgery, and baseline fungal risk.	All reports	Assess applicability and baseline risk.	Mixed populations were eligible only when adult critical-care data were separable or the entire cohort was critical-care relevant.
Fungal target and case definition	*Candida*/ICI, candidemia, intra-abdominal candidiasis, *Aspergillus*/CAPA/IPA, *Pneumocystis*, mixed IFI, and definitions of proven/probable/possible disease.	All reports	Avoid combining biologically and clinically non-equivalent syndromes.	*Candida* and non-*Candida* treatment-effect evidence were not pooled together.

Extraction domain	Variables extracted	Applied to	Purpose	Methodological constraint
Biomarker or diagnostic strategy	Assay, manufacturer, specimen, threshold, timing, serial sampling, turnaround time, and concurrent microbiology/imaging.	All reports	Characterize the diagnostic component and timing of decision support.	Data-derived thresholds and delayed/batch testing were identified explicitly; diagnostic positivity was not treated as evidence of clinical benefit.
Algorithmic intent and decision rule	Initiation, pre-emptive treatment, withholding, interruption, early discontinuation, de-escalation, continuation, rescue treatment, or mixed reassessment.	Management-oriented reports	Define the therapeutic action triggered by the result.	Initiation and exposure-reduction pathways were interpreted separately.
Comparator and causal contrast	Randomised control, placebo, usual care, historical/before-after comparator, clinician-directed management, hypothetical strategy, or no comparator.	All reports	Determine whether a causal treatment-effect interpretation was possible.	Non-randomised, single-arm, and decision-analytic comparisons were not interpreted as randomised treatment effects.
Intervention fidelity and implementation	Proportion tested, result availability, algorithm activation, adherence, clinician override, timing of decisions, and reasons for non-implementation.	Trials and pathway studies	Assess whether the intended strategy was actually delivered.	Failure to activate or follow an algorithm was distinguished from lack of biological efficacy.
Antifungal exposure	Any systemic antifungal use, time to initiation, days of therapy, treatment duration, DDD, discontinuation, withholding, restart, consumption, and de-escalation.	Trials and management studies	Describe stewardship direction and magnitude.	Incompatible exposure constructs and scales were not pooled as a single continuous effect.
Clinical outcomes	28/30-day, ICU, hospital, or longest-follow-up mortality; clinical response; length of stay; organ-support-free days; and composite outcomes.	Randomised and narrative evidence	Summarize patient-important outcomes.	Fixed-time mortality was pooled only within compatible decision-rule strata; CandiSep rule-in mortality and non-core evidence were synthesised separately.
Mycological safety	Post-randomisation or newly diagnosed IFI, candidemia, breakthrough or new IFI, false-negative stop events, restart after discontinuation, and adjudication/follow-up window.	Trials and exposure-reduction pathways	Evaluate potential harm from withholding or stopping therapy.	Sparse events, heterogeneous definitions, and differential diagnostic surveillance limited certainty.
Diagnostic performance	Sensitivity, specificity, PPV, NPV, AUROC/AUC, likelihood/post-test probability, cutoff performance, serial testing, combinations, and calibration.	Diagnostic and linked reports	Support rule-in/rule-out plausibility and identify assay limitations.	Diagnostic accuracy was not equated with therapeutic effectiveness or safety.
Adverse events and resource use	Drug-related adverse events, toxicity-related discontinuation, treatment costs, diagnostic costs, antifungal consumption, and decision utility.	Intervention, pathway, and decision-analytic studies	Describe potential harms and resource implications.	Economic and decision-analytic outputs were retained as contextual evidence and not pooled with clinical outcomes.
Missing data, reporting, funding, and conflicts	Analysis population, withdrawals, missing assays/outcomes, selective outcome availability, funding source, in-kind assay support, and declared conflicts.	All reports	Support risk-of-bias, applicability, and interpretation judgments.	Unreported values were not imputed; internally inconsistent reported denominators were flagged rather than reconciled by assumption.
Synthesis handling	Evidence category, independent-study status, quantitative role, reason for narrative-only handling, and interpretive limitation.	All reports	Make the transition from extraction to synthesis reproducible.	Core *Candida* RCT event counts are reported in Supplementary Table S8; this table covers non-core, narrative, linked, and context-only evidence.

Panel B. Randomised evidence synthesised separately from the core *Candida* meta-analysis

These randomised comparisons were not pooled with the five core *Candida* biomarker-guided RCTs because the biomarker did not define allocation or because the population, fungal target, comparator, and decision pathway were clinically incompatible.

Study / evidence role	Population / setting	Biomarker and decision rule	Antifungal exposure / implementation	Clinical, mycological, or diagnostic outcomes	Interpretation and synthesis constraint
ST019 Verma et al., 2025 PMID 41268913; DOI 10.14309/ajg.0000000000003832	216 adults with acute-on-chronic liver failure, multiple IFI risk factors, and clinically suspected mixed IFI; 108 randomised per group.	Immediate empirical antifungal therapy versus a bundled diagnostic/biomarker-driven pre-emptive strategy. Trigger criteria included BDG (single ≥150 pg/mL or repeated ≥80 pg/mL), galactomannan (single ≥1.0 or repeated ≥0.5), cultures, imaging, and site-specific criteria. Serial biomarkers/cultures informed continuation, de-escalation, and cessation.	Antifungal therapy was initiated in 107/108 empirical-strategy patients and 89/108 pre-emptive-strategy patients. Nineteen pre-emptive-arm participants did not receive treatment because initiation criteria were not met, early death/discharge occurred, or consent/logistical issues intervened.	28-day survival was 38/108 (35%) with empirical therapy and 14/108 (13%) with the diagnostic/biomarker-driven strategy. Treatment success was 40/107 (37.4%) versus 15/89 (16.9%; P=0.002). New/breakthrough IFI occurred in 10.3% versus 16.9% (P=0.255); toxicity-related discontinuation occurred in 2 versus 3 patients.	Separate randomised evidence only. The highly selected liver-ICU population, mixed-fungal target, immediate-treatment contrast, and bundled diagnostic pathway preclude pooling with *Candida* RCTs and do not isolate the effect of an individual biomarker.

Panel C. Included structured narrative and linked-report outcome extraction

The rows below summarise management, exposure, safety, diagnostic, implementation, and decision-utility signals from thirteen primary narrative studies and two linked reports. Linked reports did not add independent participants.

Study / evidence role	Population / setting	Biomarker and decision rule	Antifungal exposure / implementation	Clinical, mycological, or diagnostic outcomes	Interpretation and synthesis constraint
ST009 Prattes et al., 2014 PMID 25040144; DOI 10.1111/myc.12221	66 ICU patients with clinically suspected IFI.	Serum BDG was obtained before the treatment decision; <60 pg/mL supported stopping, >120 pg/mL supported initiation, and intermediate results prompted repeat testing.	Initial therapy was started in 40 patients and withheld in 26. BDG changed the initial decision in 20/66: therapy was discontinued in 13 and initiated in 7.	None of the 13 patients whose therapy was discontinued developed IFI.	Retrospective, clinician-mediated, mixed-intent analysis without a concurrent protocolised comparator; no causal efficacy or safety inference.

Study / evidence role	Population / setting	Biomarker and decision rule	Antifungal exposure / implementation	Clinical, mycological, or diagnostic outcomes	Interpretation and synthesis constraint
ST010 Martínez-Jiménez et al., 2015 PMID 26311840; DOI 10.1093/jac/dkv241	100 adults receiving empirical antifungal therapy for suspected IC; 63 were in ICU.	Serial CAGTA and BDG were measured on days 0, 3, and 5, but results were blinded to treating clinicians. The study evaluated a potential serial-negative rule-out strategy.	Median empirical-treatment duration was 10 days. All biomarkers were negative in 31/58 patients without IC; the authors estimated that acting on these results could have supported discontinuation in at least 31% of the cohort.	Combined CAGTA/BDG sensitivity and NPV were 96.7% and 97.1% overall and 100%/100% in the ICU subgroup.	Diagnostic-management plausibility only. Because results were blinded, the report did not evaluate actual biomarker-guided discontinuation or clinical safety after stopping.
ST011 Posteraro et al., 2016 PMID 27125554; DOI 10.1093/jac/dkw112	198 septic ICU adults with *Candida* score ≥3; 47 had candidemia and 16 were classified as probable *Candida* infection.	Routine-care BDG (positive ≥80 pg/mL) informed initiation, avoidance, or early discontinuation.	Among 135 BDG-negative patients, 110 received no antifungal therapy. Of 25 initially treated BDG-negative patients, therapy was stopped after the result in 14. All 63 BDG-positive patients received early antifungal therapy.	All 47 candidemia episodes were BDG-positive and 135/151 patients without candidemia were BDG-negative (descriptive sensitivity 100% and specificity 89.4% in this cohort). The 97.9%/89.3% pair discussed in the article referred to a prior study and was not extracted as this cohort's estimate.	Retrospective observational management study with clinician selection and no randomised comparator. Diagnostic performance and exposure associations cannot establish treatment benefit or safety.
ST012 Nucci et al., 2016 PMID 27287231; DOI 10.1093/jac/dkw188	85 high-risk ICU adults receiving empirical anidulafungin in eight ICUs.	Blood cultures were obtained on days 1-2 and BDG on days 1-3. On day 4, therapy was stopped after negative cultures and three negative BDG tests; positive-biomarker and candidemia cohorts continued treatment.	All 21 patients in the negative-biomarker cohort discontinued anidulafungin on day 4.	No recurrent candidemia occurred through day 30 in the negative-biomarker cohort. Thirty-day mortality was 9/21 (42.8%); mortality differed non-significantly across the three non-randomised cohorts (*P*=0.28).	Prospective but non-randomised pilot cohort. Small groups, selected high-risk population, and non-comparable cohorts preclude confirmation of mycological safety.
ST013 Gill et al., 2019 PMID 31248660; DOI 10.1016/j.diagmicrobio.2019.04.016	206 ICU adults with suspected candidemia, negative blood cultures, and a negative rapid diagnostic test; 103 were evaluated in each period.	A negative T2Candida/T2MR pathway replaced a negative BDG pathway and was paired with stewardship review and encouragement to discontinue empirical anidulafungin.	Median anidulafungin duration was 2 days (IQR 1-5) with BDG and 1 day (IQR 1-2) with T2MR (*P*<0.001). Turnaround time was 3.8 versus 0.4 days. T2MR was associated with early discontinuation (adjusted OR 3.1, 95% CI 1.7-5.6). Therapy was restarted in 17% versus 13%.	Subsequent proven/probable candidemia after discontinuation occurred in 9% versus 3% (*P*=0.134). Crude mortality was higher in the later T2MR period (60% vs 43%; *P*=0.018), alongside higher baseline SOFA scores.	Retrospective before/after quasi-experiment with temporal and severity confounding. The mortality difference should not be attributed to T2MR; safety events were uncommon and not randomised.

Study / evidence role	Population / setting	Biomarker and decision rule	Antifungal exposure / implementation	Clinical, mycological, or diagnostic outcomes	Interpretation and synthesis constraint
ST014 Hare et al., 2020 PMID 34368697; DOI 10.1016/j.infpip.2020.100047	109 critical-care antifungal-treatment episodes after implementation of a diagnostic-driven pathway.	BDG was embedded in 48-72-hour reassessment. Pathway compliance was audited when results were available.	BDG was sent in 95/109 episodes and available at decision review in 60/95; 38/60 (63%) were managed according to the pathway. In colonized/no-IC episodes, median empirical-treatment duration was 5.5 days (IQR 4-7) with compliance versus 14.5 days (IQR 9-23) without compliance (*P*<0.001).	One proven IC event within 7 days occurred among 14 compliant colonized/no-IC episodes; 30-day mortality was 1/14 versus 1/16. Weekly testing did not produce an observed reduction in overall anidulafungin consumption.	Prospective service audit rather than a controlled intervention trial. Compliance comparisons are highly susceptible to selection, timing, and clinician-behaviour confounding.
ST015 Helweg-Larsen et al., 2021 PMID 34947026; DOI 10.3390/jof7121044	219 non-neutropenic ICU patients with sepsis and high risk of IC in two tertiary ICUs.	T2Candida PCR and *Candida* mannan antigen were used within a prospective stewardship programme to support early initiation, discontinuation, or abstention.	Positive testing contributed to early (<3-day) initiation in 13/101 patients. Negative paired testing contributed to rapid (<4-day) discontinuation or no therapy in 12/50 (24%). Overall, 52/219 (24%) received no antifungal therapy during hospitalisation.	For tests obtained within 3 days of therapy, combined sensitivity/specificity/PPV/NPV were 70%/90%/63%/93%. No reduction in overall antifungal use was demonstrated across comparison periods.	Prospective programme without randomised allocation; treatment decisions and temporal consumption comparisons were clinician-mediated and confounded.
ST016 Carbonell et al. (CAPA-IP), 2022 PMID 35884988; DOI 10.3390/biomedicines10071683	200 adults with critical COVID-19; 164 required invasive ventilation and tracheal-aspirate galactomannan was tested in 93 deteriorating patients.	A proposed CAPA-in-progress definition used tracheal-aspirate galactomannan index ≥0.5 and triggered pre-emptive systemic antifungal treatment.	Nineteen patients met CAPA-in-progress criteria. Seventeen were treated as soon as results became available; two died on the day of diagnosis. Median treatment duration was 19 days (range 3-39).	ICU mortality was 10/19 (52.6%) in CAPA-in-progress versus 50/145 (34.5%) in other ventilated patients (*P*=0.036); median ICU stay was 27 versus 11 days (*P*=0.008).	Prospective observational diagnostic-management study. Comparisons identify disease/severity associations and cannot establish benefit from pre-emptive treatment.
ST017 Salhotra et al., 2025 PMID 40416540; DOI 10.5005/jp-journals-10071-24961	100 ICU patients underwent BDG testing; the comparative analysis included 53 BDG-negative patients, 22 treated and 31 untreated.	Negative BDG (<60 pg/mL) was evaluated as support for withholding antifungal therapy; treatment remained clinician-directed.	22/53 BDG-negative patients received antifungal therapy and 31/53 did not.	Unadjusted ICU survival was 10/22 (45.5%) versus 25/31 (80.6%; *P*=0.008), with greater illness severity in treated patients. Antifungal therapy was not an independent survival predictor (reported OR 0.363; *P*=0.156). Two subsequent proven infections were C. auris.	Retrospective and confounded by indication. The published propensity-score section is internally inconsistent (it reports 31 matched pairs despite only 22 treated patients); its matched estimate should therefore not be relied upon without author clarification.

Study / evidence role	Population / setting	Biomarker and decision rule	Antifungal exposure / implementation	Clinical, mycological, or diagnostic outcomes	Interpretation and synthesis constraint
ST018 Zhao et al., 2025 PMID 40503829; DOI 10.1128/spectrum.00584-25	171 immunocompromised critically ill adults evaluated for IPA across six tertiary ICUs (67 IPA; 104 non-IPA).	BAL-fluid mNGS was compared with conventional microbiological testing; clinicians used results to adjust antimicrobial regimens.	Among IPA patients, regimen adjustments based on mNGS were classified as having a positive clinical impact in 90.70% versus 50.0% for conventional testing (P<0.05).	mNGS sensitivity/specificity/AUC were 94.03%/96.20%/0.951 versus 95.52%/78.85%/0.872 for combined conventional tests. Reported 28-day mortality was 46.51% with mNGS-guided adjustments versus 66.67% with conventional-test-guided adjustments (P<0.05).	Retrospective observational study with clinician-selected regimen changes; adjustments were broad antimicrobial interventions, and associations cannot be interpreted as causal antifungal effects.
ST020 Rinaldi et al. (LAMBDA), 2023 PMID 37838147; DOI 10.1016/j.ijantimicag.2023.106998	40 adults with sepsis after major intra-abdominal surgery and high risk of intra-abdominal candidiasis.	All patients received liposomal amphotericin B 5 mg/kg on day 1. On day 3, therapy stopped if baseline BDG was <80 pg/mL, continued at 3 mg/kg/day if BDG was >200 pg/mL or IAC was confirmed, and was serially guided for intermediate values.	Liposomal amphotericin B was discontinued in 26/40 (65%) after a negative baseline BDG result; standard-dose continuation occurred in 35%.	IAC was excluded in 33 patients, probable in 5, and proven in 2. No grade 3/4 severe drug-related adverse event was reported. Thirty-day mortality was 6/40 (15%); no death was attributed to amphotericin B or uncontrolled IAC.	Prospective single-arm phase 2 intervention study without a concurrent comparator. Findings support feasibility/tolerability, not comparative effectiveness or definitive safety.
ST021 Albanell-Fernández et al., 2026; PMID 42497386; DOI 10.1093/jac/dkag226	Prospective single-centre study of 90 critically ill adults receiving empirical echinocandin therapy; 83 without confirmed fungal infection entered the main BDG-positive versus BDG-negative exposure analysis.	Wako BDG was obtained within 48 h; ≥7 pg/mL defined positivity. Results were integrated with *Candida* score, clinical trajectory, cultures, imaging and specialist review. Negative results supported early discontinuation; positive results prompted assessment for false positivity, repeat testing and individualised continuation or de-escalation.	Among patients without confirmed fungal infection, early discontinuation occurred in 44/57 (77.2%) BDG-negative versus 5/26 (19.2%) BDG-positive patients (P<0.001). Median echinocandin duration was 2 [1-3] versus 4 [2-7] days (P=0.004) and total antifungal duration 3 [2-4] versus 8 [4-13] days (P<0.001). Stop recommendations were accepted in 45/57 (78.9%). Median echinocandin cost was €541.7 versus €1083.4.	Five *invasive candidiasis* cases occurred, all in the high-risk stratum; two had initially negative BDG and later positive cultures. Subsequent fungal infection within 30 days occurred in 4/57 versus 4/26 (P=0.232), mortality during treatment in 6/57 versus 5/26 (P=0.278), and 30-day post-treatment mortality in 18/57 versus 11/26 (P=0.342). In high-risk patients, sensitivity/specificity/PPV/NPV were 60%/60.3%/11.5%/94.6%.	Prospective implementation evidence, not a causal comparison. Groups were defined by biomarker result and differed in pre-test risk; treatment decisions also reflected clinical and microbiological information. The small number of infections does not establish safety. Use to inform feasibility, false-negative risk, turnaround and specialist-supported delivery.

Study / evidence role	Population / setting	Biomarker and decision rule	Antifungal exposure / implementation	Clinical, mycological, or diagnostic outcomes	Interpretation and synthesis constraint
ST022 Abi Kheir et al., 2026; PMID 42186584; DOI 10.1177/87551225261446911	Retrospective multisite pre-post cohort across six ICUs in one tertiary health system; 55 standard-of-care and 55 algorithm-period non-neutropenic adults receiving empirical micafungin.	Pharmacy-driven pathway required invasive-candidiasis risk assessment, BDG and blood cultures, daily pharmacist review and structured recommendations. BDG <60 pg/mL was negative, 60-80 indeterminate and >80 positive. Negative results were intended to support stopping after 48 h reassessment; positive/indeterminate results prompted continuation and reassessment.	Overall micafungin duration did not differ: 95.4 [49.2-206.4] h with the algorithm versus 94.7 [45.0-146.3] h with standard care (*P*=0.840). Mean result time was 60.1 ±30.9 h. Results within 48 h were associated with 53.0 [46.1-98.1] versus 140.7 [72.8-226.6] h (*P*=0.014). Among 29 negative results, 20 led to discontinuation within 24 h and 9 did not; 7/26 positive/indeterminate results were also followed by discontinuation. Protocol-concordant negative-result stopping was associated with 47.6 [35.6-68.8] h (*P*=0.005 versus standard care).	Hospital mortality was 24/55 in both groups; confirmed *invasive candidiasis* occurred in 4/55 versus 1/55 (*P*=0.363), ICU LOS 10 versus 8 days (*P*=0.720), hospital LOS 27 versus 26 days (*P*=0.760), and 30-day readmission 8/55 versus 14/55 (*P*=0.160). Sensitivity/specificity/NPV/PPV were 75%/55%/96.6%/11.5%; dialysis was associated with false positivity.	Historical pre-post implementation study at serious risk of confounding and selection bias. The primary programme-level effect was null. Timely-result and adherence comparisons were post-baseline, non-randomised and susceptible to indication/selection bias; they are implementation signals, not causal treatment effects or safety estimates.
ST011-L Giacobbe et al., 2019 PMID 30506332; DOI 10.1007/s10096-018-3441-1	Post hoc decision-analytic re-analysis of the Posteraro cohort; 186/198 parent-cohort patients were included after excluding 12 with suspected deep-seated candidiasis.	Observed BDG-based early decisions were compared with a hypothetical strategy of empirical antifungal therapy for all patients using DOOR rankings.	The analysis ranked early treatment/withholding choices and associated treatment costs rather than measuring a new intervention.	The BDG-based strategy had a 67.81% probability of a more cost-effective choice (95% CI 67.32-68.30), 19.68% probability of a less cost-effective choice, and 12.50% probability of an equivalent choice.	Linked report; no additional independent population. Post hoc decision model based on observational data and a hypothetical comparator; not a clinical treatment-effect estimate.
ST004-L Standl et al., 2026 PMID 42023876; DOI 10.1128/jcm.01500-25	Linked CandiSep diagnostic analysis of 339 participants after three withdrawals; 48 had ICI and 14 candidemia.	BDG, two mannan-antigen assays, and five anti-*Candida* antibody assays were evaluated at manufacturer and data-derived cutoffs, alone and in combinations.	No new therapeutic intervention or exposure contrast was evaluated; the report informed threshold and assay interpretation only.	At 80% specificity, sensitivity for ICI/candidemia was 46%/64% for BDG, 38%/64% for Platelia mannan, and 38%/50% for Serion mannan. Antibody assays performed poorly and were influenced by colonisation. Simultaneous or sequential combinations did not improve diagnostic performance over single antigen assays.	Linked report; no additional independent participants. Cutoffs were cohort-derived, candidemia events were few, confidence intervals were wide, and the analysis does not establish therapeutic effectiveness.

TABLE S3. STATISTICAL RULES, ENDPOINT DEFINITIONS, POOLING CRITERIA, AND SENSITIVITY ANALYSES

This table specifies the analytic hierarchy. The review was prospectively registered in PROSPERO (CRD420261432481) on 25 June 2026. The intervention-synthesis eligibility framework required an explicit link between a fungal biomarker or fungal diagnostic result and an antifungal management decision; this criterion was applied during full-text/report-level assessment. Reports in which biomarkers were ancillary and did not define the management strategy were excluded and are documented in Supplementary Table S4. Random-effects meta-analysis was prespecified in PROSPERO, but the registration did not name a variance estimator. Before quantitative pooling, DerSimonian-Laird inverse-variance analysis was selected as the primary calculation. Exposure-reduction mortality and post-randomisation invasive *Candida* events were prespecified decision-specific analyses. The rule-in treatment-receipt pool was exploratory. REML/Wald, modified Hartung-Knapp, Mantel-Haenszel, risk-difference, proven-only Rouzé, leave-one-out and missing-outcome analyses, together with the low-activation Erb exclusion, were post hoc robustness checks. The ACLF randomised comparison remained separate from *Candida* evidence, and LAMBDA remained narrative because it had no concurrent comparator.

Panel A. Statistical estimand, model, and calculation rules

Item	Rule applied	Rationale / safeguard
Unit of analysis	Independent randomised study population; linked reports did not contribute additional participants.	Prevents double-counting across primary clinical reports and linked diagnostic-performance reports.
Primary quantitative evidence set	Core *Candida* biomarker-guided randomised trials in critically ill adults.	Restricts treatment-effect estimation to randomised comparisons in which the biomarker was part of the therapeutic decision algorithm.
Effect measure for dichotomous outcomes	Crude risk ratio (RR) with 95% confidence interval (CI).	Maintains direct clinical interpretability and consistent direction of effect across outcomes.
Log-scale pooling	RRs were analysed on the natural logarithmic scale. Study-level standard errors were calculated from 2 x 2 event counts.	Standard inverse-variance approach for pooled RR estimates.
Formula used for study-level SE	SE[ln(RR)] = sqrt(1/a − 1/n1 + 1/c − 1/n0), where a and c are events and n1 and n0 are the analysis denominators reported for that outcome.	Applied to published outcome-analysis populations after continuity correction when required; randomised and analysed denominators are reported separately.
Zero-event cells	For inverse-variance log-RR calculations, 0.5 was added to all four cells when one randomised arm contained zero events. Double-zero studies were non-informative for RR pooling. Mantel-Haenszel pooled RRs used uncorrected counts because a single-zero arm remains estimable.	Makes the only continuity correction explicit and avoids unnecessarily correcting Mantel-Haenszel estimates.
Minimum threshold for pooling	Pooling required at least two informative randomised comparisons with compatible endpoint definition, time point, fungal target, and algorithmic intent.	Prevents quantitative synthesis from single-study or construct-incompatible evidence.

Item	Rule applied	Rationale / safeguard
Primary model	Random-effects inverse-variance meta-analysis using the DerSimonian-Laird estimator for between-study variance.	Random-effects synthesis was prespecified in PROSPERO. The registration did not name a variance estimator; DerSimonian-Laird was selected before quantitative pooling and was accompanied by small-sample sensitivity analyses.
Small-sample uncertainty analysis	REML estimated τ^2. With $w_i=1/(v_i+\tau^2)$, $q=\Sigma w_i(y_i-\mu)^2/(k-1)$, SE_HK=$\sqrt{(q/\Sigma w_i)}$, and SE_mHKSJ=max[SE_REML/Wald, SE_HK]; the interval used $t(k-1)$ critical values. Mantel-Haenszel RRs were also reported.	The explicit variance safeguard prevents a modified HKSJ interval from being narrower than the REML/Wald interval. With k=2 it remains a conservative stress test, not a mechanical inferential rule.
Sparse-data interval sensitivity	For fungal safety, RR analyses were supplemented with risk-difference analyses using uncorrected arm risks; alternative zero-cell handling was used as a sensitivity rather than a replacement for the primary RR analysis.	Shows whether conclusions depend on the 0.5 zero-cell continuity correction and emphasizes absolute uncertainty.
Heterogeneity	Cochran Q, tau-squared, and I-squared were reported when pooling was performed.	I-squared was not overinterpreted when the number of studies was small.
Two-sided *P* values	*P* values were derived from the inverse-variance log-RR model.	Reported alongside RR, 95% CI, and heterogeneity statistics.
Decision-rule definitions	Rule-in: a result-contingent increase in treatment initiation/acceleration. Rule-out: a result-contingent reduction in continuation of already-started therapy. Risk-adaptive hybrid: immediate cover in a prespecified high-risk stratum followed by a result-informed continuation decision.	Separates observed randomised strategies from a conceptual hybrid trial construct and assigns direction-specific success criteria.

Panel B. Endpoint definitions and pooling decisions

Endpoint / evidence domain	Definition used for extraction	Pooling and reporting decision
Global 28- or 30-day mortality	All-cause mortality at 28 or 30 days in core *Candida* biomarker-guided RCTs with comparable short-term endpoints.	Retained only in Supplementary Table S8 as a descriptive contextual aggregation. It is absent from the main forest inference, is not interpreted as a unitary intervention effect, and has no unitary GRADE rating.
Hanson ICU-discharge survival	Survival at ICU discharge was reported as rounded percentages (85% versus 81%); exact event counts were not reported.	Narrative only. Exact death counts were not reconstructed and the outcome was not pooled.
Exposure-reduction mortality	Mortality restricted to discontinuation, interruption, or early-termination algorithms.	Pooled as the decision-specific mortality/safety analysis for discontinuation, interruption, and early-termination algorithms.
Post-randomisation invasive *Candida* events under rule-out assignment	Invasive *Candida* events first diagnosed after randomisation or explicitly reported as subsequent/follow-up under assignment to an exposure-reduction strategy. Infections present at enrolment were excluded when reports separated them. For Rouzé, the prespecified proven-or-probable endpoint (4/54 versus 1/55) was primary; proven-only events were examined in sensitivity analysis.	Pooled as an intention-to-treat policy-safety analysis. It does not estimate safety only among patients in whom the stopping gate opened. The proven-only sensitivity was not substituted for the prespecified endpoint.
Systemic antifungal use in initiation/pre-emptive algorithms	Proportion of patients receiving systemic antifungal therapy in initiation or pre-emptive core RCTs.	Pooled as exploratory exposure-direction analysis; interpreted as antifungal exposure, not as clinical benefit.

Endpoint / evidence domain	Definition used for extraction	Pooling and reporting decision
Early antifungal treatment within 96 hours	CandiSep single-study endpoint describing early antifungal administration.	Reported descriptively/illustratively only; not pooled because it was a single-study endpoint.
Antifungal exposure duration and consumption endpoints	Duration of therapy, days of therapy, defined daily doses, early discontinuation, interruption, and antifungal-free endpoints.	Not pooled as a single continuous or standardised effect because endpoints reflected different constructs, time windows, and implementation mechanisms.
ICU or hospital mortality	Mortality at time points other than the primary 28/30-day endpoint.	Narrative or tabular only unless endpoint timing was compatible with a prespecified mortality sensitivity.
Adverse events and serious adverse events	Any reported adverse event or serious adverse event outcome.	Narrative only; no pooled estimate because definitions, attribution, and reporting completeness differed.
Length of stay and support-free days	ICU length of stay, hospital length of stay, ventilator-free days, organ-support-free days, or related continuous endpoints.	Narrative only; no pooled estimate because scales and clinical constructs were not equivalent.
Non-randomised, pathway, LAMBDA, non-*Candida*, linked, and context-only evidence	Observational/pathway studies, the single-arm LAMBDA phase 2 pathway, diagnostic-management studies, linked reports, and diagnostic context.	Not pooled with randomised treatment-effect estimates; summarised by design and permitted inference. LAMBDA informs feasibility/tolerability only.
Separate mixed-fungal ACLF randomised strategy trial	Immediate empirical versus diagnostic/biomarker-driven pre-emptive antifungal therapy in 216 adults with ACLF; primary outcome 28-day survival.	Reported separately. Not pooled with *Candida* trials because fungal target, baseline risk, population, comparator, and bundled decision pathway were clinically incompatible.

Panel C. Sensitivity, subgroup, exploratory, and robustness analyses

Sensitivity / subgroup analysis	Rule applied	Interpretation guardrail
De Pascale missing-outcome and leave-one-out sensitivity	Restored the 60/60 randomised denominators under all-missing-alive, known day-0 death, best-case, and worst-case scenarios; also repeated the pool without De Pascale.	Assesses dependence on 12 post-randomisation exclusions; sensitivity estimates remain inconclusive and are reported in Supplementary Table S8.
Exposure-reduction mortality analysis (prespecified)	Restricted to discontinuation, interruption, or early-termination algorithms.	Decision-specific analysis; does not establish non-inferiority or safety.
Fungal-safety analysis (prespecified)	Invasive *Candida* events diagnosed after randomisation in strict-core exposure-reduction trials.	Assesses whether exposure reduction was accompanied by a detectable *Candida* safety signal; underpowered for definitive safety.
Fungal-safety robustness	Post hoc assessment of estimator choice, absolute risk, zero-cell handling, and the Rouzé proven-only definition.	Used to avoid overinterpreting rare-event estimates. The proven-or-probable endpoint remained primary; proven-only results are labelled as sensitivity.
Initiation/pre-emptive antifungal-use analysis (exploratory)	Pooled systemic antifungal use in biomarker-positive initiation/pre-emptive algorithms.	Interpreted as an exposure-increasing effect, not as evidence of patient-centred benefit; it was not presented as a prespecified primary outcome.

Sensitivity / subgroup analysis	Rule applied	Interpretation guardrail
CandiSep early antifungal-use endpoint	Single-study RR for antifungal treatment within 96 hours.	Illustrates magnitude of early exposure acceleration; not a pooled endpoint.
Zero-event robustness (post hoc analytic sensitivity)	Continuity correction documented whenever a zero-event cell affected RR calculation.	Prevents hidden analytical choices in sparse outcomes.
Model-choice robustness (post hoc analytic sensitivity)	REML/Wald, modified HKSJ, and Mantel-Haenszel estimates were compared with the a priori DerSimonian-Laird primary calculation; risk difference was added for sparse fungal safety.	Tests dependence on estimator and zero-cell handling without changing the prespecified clinical estimand or replacing the a priori primary calculation after seeing the results.
Biomarker-type subgrouping	Descriptive comparison by biomarker class only when clinically meaningful.	No formal interaction testing was performed because study numbers were too small.
Direction-of-effect checks	Outcome direction checked so RR > 1 consistently meant more events in the biomarker-guided arm.	Avoids interpretation errors across mortality, fungal events, and antifungal exposure.
Risk-adaptive hybrid	No pooled estimate. ACLF and LAMBDA were used only to define a comparative trial question with delay, later exposure, and fungal-safety co-criteria.	Conceptual trial construct; not a validated phenotype or current standard of care.

Panel D. Non-pooling rules and reporting safeguards

Safeguard	Rule applied	Purpose
No pooling of observational/pathway studies with RCTs	Non-randomised and pathway studies were summarised narratively.	Avoids confounding-driven treatment-effect estimates.
No pooling of diagnostic performance with treatment effects	Sensitivity, specificity, AUROC, cutoff, and decision-curve data were not pooled with clinical treatment outcomes.	Diagnostic discrimination is not equivalent to patient-centred treatment benefit.
No pooling of *Candida* and non-*Candida* evidence	*Candida*-focused randomised evidence was analysed separately from non-*Candida* or mixed fungal diagnostic-management evidence.	Avoids combining distinct pathogens, diagnostics, treatment indications, and outcome mechanisms.
Separate handling of the ACLF randomised trial	The ACLF trial was retained as randomised evidence but not pooled with *Candida* trials.	Preserves the randomised result without creating a clinically incoherent combined estimate.
No double-counting of linked reports	Giacobbe was linked to Posteraro and Standl to CandiSep; each was retained only for unique decision-analytic or diagnostic-performance information.	Prevents adding participants from the same study populations twice while preserving report-level interpretive contributions.
No pooling of initiation and discontinuation endpoints as one stewardship construct	Initiation/pre-emptive and exposure-reduction algorithms were interpreted separately.	Biomarker positivity used to start therapy and biomarker negativity used to stop/withhold therapy address different clinical decisions.
No standardised mean difference for heterogeneous exposure endpoints	Duration, consumption, discontinuation, interruption, and antifungal-free endpoints were not collapsed into one standardised continuous effect.	Avoids pooling endpoints with different clinical meanings.

Safeguard	Rule applied	Purpose
No funnel plot or small-study-effect test	No pooled outcome included enough randomised comparisons for a meaningful assessment.	Avoids misleading small-study-effect inference with $k < 10$.
No causal inference from context-only diagnostic evidence	*Pneumocystis* and other context-only diagnostic records were used to support evidence-gap interpretation only.	Prevents diagnostic context from being presented as stewardship treatment evidence.
Reproducibility and audit trail	Extracted randomised event counts, study-level effects, sensitivity analyses, and outcome-specific pooling decisions are reported in Supplementary Table S8.	Ensures that all quantitative claims can be traced to study-level data and prespecified rules.
Search update and protocol-to-report transparency	PubMed, Scopus and CENTRAL were updated through 24 July 2026 using the same search concepts; ClinicalTrials.gov and WHO ICTRP had been searched on 29 June 2026. The database update returned 31 raw hits, 26 update-unique records, 22 prior-master overlaps and four genuinely new records. Albanell-Fernández was newly identified and included after full-text assessment; three new records were excluded at title/abstract. Abi Kheir was already present in the original master and proceeded to full-text assessment during reconciliation. The intervention-synthesis eligibility framework required an explicit biomarker-to-antifungal-action link and was applied during full-text/report-level assessment; three antifungal-strategy RCT reports in which biomarkers were ancillary and did not define the allocated intervention were excluded.	Separates raw search yield, deduplication, prior-master reconciliation, report-level eligibility decisions, and post hoc analytic robustness from outcome-driven changes to primary inference.

TABLE S4. REPORTS EXCLUDED AFTER FULL-TEXT OR REPORT-LEVEL ELIGIBILITY ASSESSMENT

This table documents the ten reports excluded after full-text or report-level assessment. Exclusion required failure to meet the intervention-synthesis criteria: an evaluable link between a fungal biomarker or fungal diagnostic result and an antifungal management decision or stewardship outcome. Diagnostic accuracy alone, hypothetical management implications, service evaluations without a defined biomarker-guided strategy, small diagnostic case series without an evaluable stewardship effect, broad non-fungal-specific molecular diagnostic pathways, and randomised antifungal-strategy trials in which biomarkers were ancillary rather than allocation-defining were not eligible for the intervention synthesis.

Panel A. Summary of full-text/report-level exclusion categories

Exclusion category	Count	Reports
Diagnostic-only or diagnostic-performance study without an antifungal-management intervention	3	Talento 2017; Calvet 2024; Elnagar 2026
Antifungal stewardship service evaluation without a defined biomarker-guided management strategy	1	Logan 2022
Diagnostic algorithm or early-recognition report without an evaluable stewardship effect	2	Hamam 2021; Shen 2023
Broad, non-fungal-specific molecular diagnostic strategy outside the intervention scope	1	Guo 2026
Randomised antifungal-strategy trial in which the fungal biomarker was ancillary and did not define the allocated intervention	3	MSG-01; Knitsch 2015; EMPIRICUS
Total	10	31 reports assessed - 10 excluded = 21 included reports

Panel B. Report-level exclusion decisions

Report and identifiers	Design and population	Biomarker / diagnostic strategy	PRISMA exclusion category	Full-text rationale and handling
Talento 2017 PMID 28384600 DOI 10.1016/j.jcrc.2017.03.025	Prospective observational diagnostic-performance study; 100 patients receiving mixed-specialty critical care for at least 7 days.	Serial serum BDG, serum/BAL galactomannan, and *Aspergillus* fumigatus PCR; diagnostic classification and assay-performance assessment.	Diagnostic-only/performance study without an antifungal-management intervention.	Biomarker assays were performed in batches and results were not available to intensivists. Antifungal work-up and treatment were physician-directed rather than protocol-driven. The report evaluated diagnostic performance and hypothetical stewardship implications, not a biomarker-linked treatment intervention or evaluable antifungal exposure/safety effect. Handling: Excluded from intervention synthesis; may be cited only as diagnostic background.

Report and identifiers	Design and population	Biomarker / diagnostic strategy	PRISMA exclusion category	Full-text rationale and handling
Logan 2022 PMID 35756574 DOI 10.1093/jacamr/dlac055	Prospective multisite service evaluation of 4,781 ICU admissions; 305 patients received antifungal therapy.	Description of antifungal consumption, prescribing rationale, post-prescription review, de-escalation, BDG turnaround time, and treatment duration by BDG result.	No defined biomarker-guided management strategy.	The evaluation described routine prescribing and compared treatment duration across positive, negative, and untested BDG groups. It did not test a predefined biomarker-based start/stop algorithm, allocate management according to biomarker results, or estimate a biomarker-guided intervention effect. De-escalation reflected routine multidisciplinary review rather than a specified fungal-biomarker pathway. Handling: Excluded from intervention synthesis; retained as background on ICU antifungal prescribing and implementation barriers.
Calvet 2024 PMID 39083132 DOI 10.1186/s13613-024-01337-8	Diagnostic interpretation and probability-modelling study using published test-performance estimates and a cohort of 2,243 immunocompromised adults with acute respiratory failure.	Bayesian post-test probabilities for *Pneumocystis jirovecii* pneumonia according to PCR and BDG results across clinical-risk subgroups.	Diagnostic-only/performance study without an antifungal-management intervention.	The study modelled diagnostic probabilities and clinician interpretation. It did not evaluate a treatment algorithm, antifungal or anti-*Pneumocystis* initiation/withholding, treatment duration, stewardship exposure, or management-related safety outcomes. Handling: Excluded from intervention synthesis; retained only as *Pneumocystis* diagnostic context.
Hamam 2021 PMID 33683480 DOI 10.1186/s13613-021-00827-3	Retrospective pilot diagnostic-classification study using 35 pathology-proven IPA cases from the French RESSIF database; 27 had complete data.	Comparison of AspICU and a biomarker-enriched BM-AspICU classification incorporating galactomannan and *Aspergillus* qPCR.	Diagnostic algorithm without an evaluable therapeutic-management or stewardship outcome.	The report tested whether a biomarker-enriched case definition reclassified known pathology-proven IPA cases. It did not implement the algorithm prospectively, measure algorithm-triggered antifungal decisions, or report antifungal exposure, de-escalation, restart, or management-related safety outcomes. Handling: Excluded from intervention synthesis; retained only as non-*Candida* diagnostic-algorithm context.
Shen 2023 PMID 37026960 DOI 10.1097/MD.0000000000033399	Prospective observational diagnostic report of 31 hematological patients with pulmonary infection, including seven severe *Pneumocystis* cases identified by blood mNGS.	Peripheral-blood mNGS for early *Pneumocystis* recognition; subsequent trimethoprim/sulfamethoxazole with or without caspofungin was described.	Early-recognition case series without an evaluable stewardship effect.	Only seven mNGS-identified *Pneumocystis* cases contributed to the management description. There was no comparator, predefined diagnostic-treatment pathway, antifungal-exposure endpoint, or evaluable estimate of the effect of mNGS-guided management on stewardship or safety. The report therefore functioned as a small diagnostic-management case series. Handling: Excluded from intervention synthesis; retained only as molecular-diagnostic background.
Elnagar 2026 PMID 41365233 DOI 10.1016/j.diagmicrobio.2025.117212	Diagnostic cohort study of 79 severely ill COVID-19 ICU patients.	Serum BDG and galactomannan evaluated against ECMM/ISHAM and modified AspICU CAPA definitions.	Diagnostic-only/performance study without an antifungal-management intervention.	The report estimated CAPA incidence, risk factors, outcomes, and diagnostic performance. It did not test a biomarker-triggered antifungal strategy or report treatment initiation, discontinuation, exposure, override, or management-related safety outcomes attributable to the biomarker result. Handling: Excluded from intervention synthesis; retained only as CAPA diagnostic context.
Guo 2026 DOI 10.3389/fcimb.2025.1724935	Multicentre retrospective propensity score-matched study of 162 immunocompromised patients with opportunistic pulmonary infections.	BAL-fluid mNGS versus conventional microbiological testing; broad antimicrobial escalation/de-escalation and clinical outcomes were assessed.	Broad, non-fungal-specific molecular diagnostic strategy outside the intervention scope.	The population included mixed opportunistic infections and the intervention was a pan-pathogen molecular diagnostic pathway. Reported treatment modifications were broad antimicrobial changes and were not extractable as a fungal-specific antifungal decision strategy. The study therefore did not estimate fungal biomarker-guided antifungal stewardship. Handling: Excluded from intervention synthesis; may be discussed only as broad molecular-diagnostic context.

Report and identifiers	Design and population	Biomarker / diagnostic strategy	PRISMA exclusion category	Full-text rationale and handling
Ostrosky-Zeichner et al. (MSG-01), 2014; PMID 24550378; DOI 10.1093/cid/ciu074	Phase 4, multicentre, double-blind, placebo-controlled randomised trial in 222 high-risk ICU adults.	Twice-weekly BDG monitoring and a clinical prediction rule were ancillary assessments within a caspofungin-versus-placebo antifungal-strategy trial.	Randomised antifungal-strategy trial in which the fungal biomarker was ancillary and did not define the allocated intervention.	Randomisation allocated caspofungin prophylaxis or placebo; biomarker results did not determine treatment allocation or define the randomised therapeutic algorithm. The trial therefore estimates an antifungal-drug strategy rather than a fungal biomarker-guided stewardship policy. Handling: Excluded after full-text reassessment; not included in narrative or quantitative synthesis.
Knitsch et al., 2015; PMID 26270686; DOI 10.1093/cid/civ707	Exploratory, multicentre, double-blind, placebo-controlled trial in 241 surgical ICU adults undergoing surgery for intra-abdominal infection.	BDG was an exploratory predictor available in a subset; pre-emptive micafungin was randomised on clinical-risk grounds and was not triggered by BDG.	Randomised antifungal-strategy trial in which the fungal biomarker was ancillary and did not define the allocated intervention.	The allocated contrast was micafungin versus placebo, not a biomarker-linked management rule. Incomplete exploratory BDG measurements cannot convert the randomised drug comparison into a biomarker-guided intervention. Handling: Excluded after full-text reassessment; not included in narrative or quantitative synthesis.
Timsit et al. (EMPIRICUS), 2016; PMID 27706483; DOI 10.1001/jama.2016.14655	Multicentre, double-blind, placebo-controlled randomised trial in 260 ICU adults with ICU-acquired sepsis, Candida colonisation, and multiple organ failure.	Serial BDG was measured as a secondary/ancillary variable; empirical micafungin for 14 days was compared with placebo irrespective of biomarker result.	Randomised antifungal-strategy trial in which the fungal biomarker was ancillary and did not define the allocated intervention.	Biomarker results did not determine randomised allocation, treatment initiation, or duration. The study estimates empirical micafungin rather than a fungal biomarker-guided stewardship strategy. Handling: Excluded after full-text reassessment; not included in narrative or quantitative synthesis.

TABLE S5. CHARACTERISTICS OF INCLUDED REPORTS AND SYNTHESIS CLASSIFICATION

This table presents report-level design, population, fungal target, diagnostic or biomarker strategy, therapeutic decision function, synthesis classification, and counting status for all included reports. Multiple reports from the same underlying population were linked and counted once at study level. Diagnostic-performance and decision-analytic reports were retained only for their specific interpretive role and did not add participants to therapeutic meta-analysis.

Panel A. Counting conventions

Evidence category	Reports / independent studies	Counting and synthesis rule
Core *Candida* biomarker-guided randomised trials	5 reports / 5 independent RCTs	Five RCTs randomised 677 participants; 661 entered the published analysis populations. Quantitative synthesis was restricted to compatible outcome definitions, time points, comparators, and decision rules.
Separate mixed-fungal randomised strategy trial	1 report / 1 independent RCT	The ACLF trial was synthesised separately because its liver-ICU population, mixed-fungal target, empirical comparator, and bundled diagnostic/biomarker pathway were not compatible with the *Candida* meta-analysis.
Structured narrative primary studies	13 reports / 13 independent studies	Non-randomised management, implementation, diagnostic-management, or biomarker-guided pathway studies. These reports were not pooled with randomised treatment-effect evidence.
Linked non-randomised decision-analytic report	1 linked report / 0 additional independent studies	Giacobbe et al. re-analysed the Posteraro cohort. The report contributes decision-analytic information but no additional independent participants.
Linked *Candida* diagnostic-performance report	1 linked report / 0 additional independent studies	Standl et al. analysed the CandiSep cohort. The report contributes diagnostic-performance information but no additional independent participants.
Overall evidence base	21 included reports / 19 independent studies	Two linked reports were retained at report level but did not add independent study populations. The unit of quantitative analysis remained the independent randomised study population.

Panel B. Report-level characteristics, classification, and quantitative role

Study ID / report	Design	Population / setting	Fungal target and test(s)	Decision rule or diagnostic function	Synthesis classification	Quantitative role and counting status
Core *Candida* biomarker-guided randomised trials						

Study ID / report	Design	Population / setting	Fungal target and test(s)	Decision rule or diagnostic function	Synthesis classification	Quantitative role and counting status
ST001 | Hanson et al., 2012 PMID 22879929; DOI 10.1371/journal.pone.0042282	Single-centre, open-label randomised pilot trial (3:1 allocation)	64 adult surgical/medical ICU patients expected to require at least 5 total ICU days and considered at increased risk of invasive candidiasis	*Candida*; twice-weekly serum β-(1→3)-D-glucan (BDG; Fungitell)	In the surveillance arm, a subsequent BDG value ≥60 pg/mL triggered pre-emptive anidulafungin; the comparator was clinician-directed empirical therapy	Core biomarker-guided RCT; initiation/pre-emptive strategy	Independent RCT. Contributed to the initiation/pre-emptive antifungal-use analysis; ICU-discharge survival (85% versus 81%) was reported narratively because exact death counts were unavailable.
ST002 | Rouzé et al. (S-TAFE), 2017 PMID 28936678; DOI 10.1007/s00134-017-4932-8	Prospective, single-centre, open-label randomised controlled trial	110 randomised; 109 analysed mixed-ICU adults receiving empirical antifungal therapy for suspected invasive *Candida* infection	*Candida*; serum BDG, mannan, and anti-mannan on days 0 and 4	A prespecified multimarker algorithm determined whether empirical therapy could be discontinued before day 7; controls followed guideline-based routine care	Core biomarker-guided RCT; prespecified exposure-reduction subgroup	Independent RCT. Contributed to the exposure-reduction mortality, post-randomisation fungal-event, and antifungal-exposure syntheses.
ST003 | De Pascale et al., 2020 PMID 32891170; DOI 10.1186/s13054-020-03265-y	Single-centre, open-label randomised trial	120 randomised; 108 included in the available-case analysis after empirical antifungal therapy had been started	*Candida*; serial serum BDG at enrolment and every 48-72 hours (Fungitell)	In the intervention arm, therapy was stopped after the first negative BDG result (<80 pg/mL); controls continued according to clinical and guideline-based criteria	Core biomarker-guided RCT; prespecified exposure-reduction subgroup	Independent RCT. Contributed to the exposure-reduction mortality, post-randomisation fungal-event, and antifungal-exposure syntheses.
ST004 | Bloos et al. (CandiSep), 2022 PMID 35708758; DOI 10.1007/s00134-022-06733-x	Open-label, multicentre randomised controlled trial in 18 German ICUs	342 adults randomised with sepsis and predefined risk factors for invasive *Candida* infection; 339 contributed to the published analysis population	*Candida*; two consecutive serum BDG samples obtained during the first 2 study days	In addition to culture-directed targeted therapy, any BDG value ≥80 pg/mL triggered antifungal initiation; continuation after discordant serial results followed the trial algorithm	Core biomarker-guided RCT; initiation strategy	Independent RCT. Contributed to the rule-in systemic antifungal-receipt synthesis and the separate CandiSep 28-day mortality estimate.
ST005 | Erb et al., 2023 PMID 37823695; DOI 10.1128/aac.00725-23	Prospective, single-centre, open-label randomised intervention study	41 adult ICU patients with newly started systemic antifungal therapy and no proven, probable, or putative fungal infection at enrolment	*Candida*; serum BDG and mannan measured on antifungal-treatment days 1 and 2	Therapy was discontinued in the intervention arm only when all BDG and mannan measurements were negative; controls received standard care	Core biomarker-guided RCT; prespecified exposure-reduction subgroup	Independent RCT. Contributed to the exposure-reduction mortality, post-randomisation fungal-event, and antifungal-exposure syntheses.

Study ID / report	Design	Population / setting	Fungal target and test(s)	Decision rule or diagnostic function	Synthesis classification	Quantitative role and counting status
Separate mixed-fungal randomised evidence						
ST019 | Verma et al., 2025 PMID 41268913; DOI 10.14309/ajg.0000000000003832	Pragmatic randomised trial	216 adults with acute-on-chronic liver failure, multiple invasive-fungal-infection risk factors, and clinical suspicion of invasive fungal infection	Mixed invasive fungal infection; BDG, galactomannan, cultures, imaging, and other mycological criteria	Immediate empirical antifungal therapy was compared with diagnostic/biomarker-driven pre-emptive therapy; prespecified stewardship rules also informed continuation, cessation, and rescue treatment	Separate mixed-fungal randomised strategy evidence	Independent RCT. Synthesised separately because the population, fungal target, comparator, and bundled pathway were incompatible with the *Candida* RCTs; no contribution to *Candida* pooling.
Structured narrative primary studies						
ST009 | Prattes et al., 2014 PMID 25040144; DOI 10.1111/myc.12221	Retrospective ICU analysis	66 ICU patients with clinically suspected invasive fungal infection	Mixed/panfungal; serum BDG obtained before antifungal initiation	BDG results prompted re-evaluation of the initial decision to initiate or discontinue systemic antifungal therapy	Structured narrative; mixed-fungal biomarker-informed management	Independent observational study. Narrative only; no randomised treatment-effect estimate.
ST010 | Martínez-Jiménez et al., 2015 PMID 26311840; DOI 10.1093/jac/dkv241	Prospective observational study	100 adults starting empirical antifungal therapy for suspected invasive candidiasis, including 63 ICU patients	*Candida*; serial CAGTA and BDG on days 0, 3, and 5	Biomarker results were blinded to treating clinicians; diagnostic analyses evaluated whether serial negative results could support future discontinuation of unnecessary empirical therapy	Structured narrative; diagnostic rule-out evidence in treated patients	Independent observational study. Narrative diagnostic-management plausibility only; no biomarker-guided treatment-effect estimate.
ST011 | Posteraro et al., 2016 PMID 27125554; DOI 10.1093/jac/dkw112	Retrospective analysis of an observational ICU cohort	198 septic ICU adults with *Candida* score ≥3 at the time of BDG testing	*Candida*; serum BDG (cutoff ≥80 pg/mL)	BDG results informed antifungal initiation, avoidance, or early discontinuation in routine clinical practice	Structured narrative; *Candida* biomarker-management evidence	Independent study population. Narrative only; parent cohort for the linked Giacobbe decision-analytic report.
ST012 | Nucci et al., 2016 PMID 27287231; DOI 10.1093/jac/dkw188	Prospective, multicentre, non-randomised pilot cohort in eight ICUs	85 high-risk ICU adults started on empirical anidulafungin	*Candida*; blood cultures on days 1-2 and serial BDG on days 1-3	On day 4, anidulafungin was discontinued after negative blood cultures and repeatedly negative BDG; positive-biomarker and candidemia cohorts continued treatment	Structured narrative; observational discontinuation pathway	Independent cohort. Narrative only; no randomised treatment-effect estimate.

Study ID / report	Design	Population / setting	Fungal target and test(s)	Decision rule or diagnostic function	Synthesis classification	Quantitative role and counting status
ST013 | Gill et al., 2019 PMID 31248660; DOI 10.1016/j.diagmicrobio.2019.04.016	Retrospective quasi-experimental study across a four-hospital health system	206 ICU adults receiving empirical anidulafungin for suspected candidemia, with negative blood cultures and a negative rapid diagnostic test	*Candida*; negative BDG strategy versus negative T2Candida/T2MR strategy	Compared rapid-test-supported discontinuation, anidulafungin days of therapy, restart, and subsequent candidemia before and after T2MR implementation	Structured narrative; rapid-diagnostic stewardship evidence	Independent non-randomised study. Narrative only; no meta-analysis.
ST014 | Hare et al., 2020 PMID 34368697; DOI 10.1016/j.infpip.2020.100047	Prospective clinical audit and service-improvement study	109 critical-care antifungal-treatment episodes after implementation of a diagnostic-driven pathway	*Candida*; serum BDG embedded in an antifungal-stewardship pathway	Prospectively audited pathway compliance, empirical-treatment duration, 30-day safety, subsequent invasive candidiasis, and antifungal consumption	Structured narrative; implementation pathway	Independent pathway study. Narrative only; no meta-analysis.
ST015 | Helweg-Larsen et al., 2021 PMID 34947026; DOI 10.3390/jof7121044	Prospective antifungal-stewardship cohort in two tertiary ICUs	219 non-neutropenic ICU patients with sepsis and high risk of invasive candidiasis	*Candida*; T2Candida PCR and *Candida* mannan antigen	Screening within an antifungal-stewardship programme supported early initiation, early discontinuation, or abstention from antifungal therapy	Structured narrative; rapid-diagnostic stewardship evidence	Independent programme study. Narrative only; no meta-analysis.
ST016 | Carbonell et al. (CAPA-IP), 2022 PMID 35884988; DOI 10.3390/biomedicines10071683	Prospective observational ICU study	200 critically ill adults with COVID-19; tracheal-aspirate galactomannan was tested in 93 clinically deteriorating patients	*Aspergillus*/CAPA; tracheal-aspirate galactomannan with culture, PCR, and BDG context	A proposed CAPA-in-progress classification (galactomannan index ≥0.5) prompted pre-emptive systemic antifungal treatment	Structured narrative; non-*Candida* diagnostic-management evidence	Independent observational study. Narrative only; no *Candida* pooling or causal treatment-effect estimate.
ST017 | Salhotra et al., 2025 PMID 40416540; DOI 10.5005/jp-journals-10071-24961	Retrospective cohort study	100 ICU patients underwent BDG testing; the comparative analysis included 53 BDG-negative patients	*Candida*/IFI risk; negative serum BDG (<60 pg/mL)	Compared ICU survival and clinical characteristics in BDG-negative patients who received versus did not receive antifungal therapy	Structured narrative; observational withholding evidence	Independent cohort. Narrative only; confounding by indication precludes causal inference.
ST018 | Zhao et al., 2025 PMID 40503829; DOI 10.1128/spectrum.00584-25	Multicentre retrospective observational study in six tertiary ICUs	171 immunocompromised critically ill adults evaluated for invasive pulmonary aspergillosis (67 IPA; 104 non-IPA)	*Aspergillus*/IPA; bronchoalveolar-lavage-fluid metagenomic next-generation sequencing (mNGS) versus conventional microbiological tests	Evaluated diagnostic performance and clinician-mediated antimicrobial-regimen escalation, de-escalation, or targeted adjustment	Structured narrative; non-*Candida* molecular diagnostic-management evidence	Independent retrospective study. Narrative only; treatment-adjustment associations were not interpreted as causal antifungal effects.
ST020 | Rinaldi et al. (LAMBDA), 2023 PMID 37838147; DOI 10.1016/j.ijantimicag.2023.106998	Prospective, single-centre, interventional phase 2 study	40 adults with sepsis after major intra-abdominal surgery and high risk of intra-abdominal candidiasis; most required ICU-level organ support	*Candida*/intra-abdominal candidiasis; baseline and serial serum BDG plus operative cultures	All patients received liposomal amphotericin B 5 mg/kg on day 1; therapy stopped on day 3 if baseline BDG was <80 pg/mL, continued if >200 pg/mL or infection was confirmed, and was serially guided for intermediate results	Structured narrative; prospective biomarker-guided intervention pathway	Independent single-arm intervention study. Narrative only; no concurrent comparator and no contribution to randomised pooling.

Study ID / report	Design	Population / setting	Fungal target and test(s)	Decision rule or diagnostic function	Synthesis classification	Quantitative role and counting status
ST021 | Albanell-Fernández et al., 2026; PMID 42497386; DOI 10.1093/jac/dkag226	Prospective, single-centre, non-randomised implementation study	90 critically ill adults receiving empirical echinocandin therapy; 83 without confirmed fungal infection entered the main result-defined exposure comparison	*Candida*; serum β-(1→3)-D-glucan measured with the Fujifilm Wako assay within 48 h; positivity threshold ≥7 pg/mL	BDG was integrated with *Candida* score, clinical trajectory, cultures, imaging and specialist review. A negative result could support early discontinuation; positive or unexplained results prompted reassessment, repeat testing and individualised continuation or de-escalation.	Structured narrative; prospective biomarker-supported rule-out implementation study	Independent study. Exposure, adherence, false-negative, subsequent-infection and cost data were synthesised narratively; no causal treatment-effect or definitive safety estimate.
ST022 | Abi Kheir et al., 2026; PMID 42186584; DOI 10.1177/87551225261446911	Retrospective, multisite, historical pre-post implementation cohort	110 non-neutropenic critically ill adults receiving empirical micafungin across six ICUs in one tertiary health system; 55 standard-care and 55 algorithm-period patients	*Candida*; serum β-(1→3)-D-glucan classified as negative <60 pg/mL, indeterminate 60-80 pg/mL or positive >80 pg/mL	A pharmacy-driven pathway combined risk-factor screening, BDG and blood cultures, daily pharmacist review and structured recommendations. Negative results were intended to support stopping after reassessment; positive/indeterminate results supported continuation and further evaluation.	Structured narrative; comparative non-randomised pharmacy-driven rule-out implementation study	Independent study. Overall programme-level micafungin duration, turnaround, adherence, outcome and diagnostic-performance data were synthesised narratively; no pooling with RCTs.
Linked reports: no additional independent study population						
ST011-L | Giacobbe et al., 2019 PMID 30506332; DOI 10.1007/s10096-018-3441-1	Post hoc decision-analytic re-analysis	Posteraro cohort: 198 ICU patients in the parent study; 186 included in the DOOR analysis after exclusion of 12 patients with suspected deep-seated candidiasis	*Candida*; observed BDG-based strategy versus a hypothetical universal empirical-treatment strategy	Applied desirability-of-outcome-ranking methods to compare early diagnostic/therapeutic choices and costs	Linked non-randomised decision-analytic report	Linked to ST011. Counted as a separate report but not as an additional independent study; not meta-analysed.
ST004-L | Standl et al., 2026 PMID 42023876; DOI 10.1128/jcm.01500-25	Linked diagnostic-performance analysis of the CandiSep randomised cohort	339 CandiSep participants analysed after three consent withdrawals from the 342 randomised parent cohort	*Candida*; BDG, two mannan-antigen assays, and five anti-*Candida* antibody assays	Evaluated manufacturer and data-derived cutoffs, AUROC, serial testing, decision curves, and simultaneous/sequential assay combinations	Linked *Candida* diagnostic-performance report	Linked to ST004. Counted as a separate report but not as an additional independent study; no participants were added to therapeutic meta-analysis.

Abbreviations: ACLF, acute-on-chronic liver failure; AFT, antifungal therapy; AUROC, area under the receiver operating characteristic curve; BDG, β-(1→3)-D-glucan; CAGTA, *Candida albicans* germ-tube antibody; CAPA, COVID-19-associated pulmonary aspergillosis; DOOR, desirability of outcome ranking; IAC, intra-abdominal candidiasis; ICU, intensive care unit; IFI, invasive fungal infection; IPA, invasive pulmonary aspergillosis; mNGS, metagenomic next-generation sequencing; RCT, randomised controlled trial; T2MR, T2 magnetic resonance.

TABLE S6. OUTCOME-SPECIFIC RISK-OF-BIAS JUDGMENTS AND DESIGN-APPROPRIATE APPRAISAL

This table appraises 21 included reports representing 19 independent studies. Randomised results were assessed with RoB 2 for the effect of assignment to intervention. Comparative non-randomised intervention-effect estimates were assessed with ROBINS-I (2016). Diagnostic-accuracy estimates were assessed at the estimate level with QUADAS-3 (version 1.2). Reports without a defensible comparative causal estimand were not assigned a pseudo-summary risk-of-bias score; instead, their design limitations and permitted use were recorded explicitly. Imprecision, inconsistency, indirectness, and publication bias are not risk-of-bias domains and are addressed separately in GRADE.

Panel A. Appraisal framework and decision rules

Evidence type	Tool / version	Unit of assessment	Judgment categories	Operational rule
Randomised trials	RoB 2, 22 August 2019; individually randomised parallel-group version	Specific result; effect of assignment to intervention	Low risk; Some concerns; High risk	Open-label status alone did not raise D2. Small sample size and early stopping were not treated as bias unless they generated a domain-specific problem.
Comparative non-randomised intervention studies	ROBINS-I, original 2016 version	Specific comparative result and explicit target-trial contrast	Low; Moderate; Serious; Critical; No information	Used only where a concurrent or historical comparison attempted to estimate an intervention effect. ROBINS-I V2 was not used because the November 2025 release remains a draft.
Diagnostic-accuracy studies	QUADAS-3, version 1.2 (2026)	Selected accuracy estimate for a stated synthesis question	Low; High; Unclear, with an overall estimate-level judgment	Participants, index test, target condition, and analysis were assessed for risk of bias; applicability was assessed for the first three domains.
Single-arm, uncontrolled, audit, and management-impact reports	Structured design-limitations appraisal	No causal treatment-effect estimand	No numerical or categorical overall score	Used to delimit feasible claims about implementation, diagnostic-management association, or hypothesis generation.
Decision-analytic report	Structured model appraisal	Decision-model result	No ROBINS-I or diagnostic-accuracy score	Assessed for source data, comparator, assumptions, uncertainty analysis, and external validity.

Low	Some concerns / Moderate / Unclear	Serious	High	Critical	No information	Combined applicability cells are shaded by the most serious component

RoB 2 domains: D1, randomisation process; D2, deviations from intended interventions; D3, missing outcome data; D4, measurement of the outcome; D5, selection of the reported result. All RoB 2 judgments below target the effect of assignment to intervention.

Panel B1. RoB 2 judgments for core *Candida* and separate mixed-fungal randomised evidence

Study / result	D1	D2	D3	D4	D5	Overall	Support for judgment
ST001 Hanson 2012 - ICU-discharge survival (descriptive)	Low	Low	Low	Low	Low	Low	Randomisation and assignment-based analysis were preserved. Observed crossover/non-adherence was treated as pathway delivery rather than a deviation caused by trial context; outcomes and antifungal receipt were objectively recorded.
ST001 Hanson 2012 - systemic antifungal use	Low	Low	Low	Low	Low	Low	Randomisation and assignment-based analysis were preserved. Observed crossover/non-adherence was treated as pathway delivery rather than a deviation caused by trial context; outcomes and antifungal receipt were objectively recorded.
ST002 Rouzé 2017 - 28-day mortality	Low	Low	Low	Low	Low	Low	Computer-generated allocation, concealed sequential opaque envelopes, intention-to-treat analysis, complete fixed-time mortality, and prospective registration support low risk.
ST002 Rouzé 2017 - early discontinuation / duration	Low	Low	Low	Low	Low	Low	The stopping algorithm and primary endpoint were prespecified. The recommendation was followed in 29/32 eligible cases; the three exceptions reflected death before recommendation or delayed laboratory availability and did not constitute differential trial-context deviations likely to bias the assignment effect.
ST002 Rouzé 2017 - subsequent proven/probable ICI	Low	Low	Low	Some concerns	Low	Some concerns	The outcome was prespecified and based on microbiology/histopathology, but unblinded clinicians could influence the intensity and timing of post-discontinuation diagnostic investigation for rare events.
ST003 De Pascale 2020 - 30-day mortality	Low	Some concerns	Some concerns	Low	Some concerns	Some concerns	Twelve of 120 randomised participants were omitted after randomisation, including protocol violations and one day-0 death in the BDG group; the analysis therefore did not fully preserve assignment. Ten percent of randomised participants lacked analysed outcomes. Missing-outcome and leave-one-out sensitivity analyses are reported in Supplementary Table S8.
ST003 De Pascale 2020 - antifungal duration / interruption	Low	Some concerns	Some concerns	Low	Some concerns	Some concerns	The post-randomisation exclusions can bias an assignment-based exposure effect, especially where exclusions were related to protocol implementation or test availability. Duration was objectively recorded, but incomplete reporting leaves uncertainty about selective analysis.
ST003 De Pascale 2020 - subsequent ICI	Low	Some concerns	Some concerns	Some concerns	Some concerns	Some concerns	In addition to post-randomisation exclusions and incomplete reporting, rare fungal events could be differentially detected in an open-label stopping trial.
ST004 CandiSep 2022 - 28-day mortality	Low	Low	Low	Low	Low	Low	Central randomisation, assignment-based analysis and objective outcome ascertainment were preserved. Imperfect execution estimates real pathway delivery and was not treated as bias from trial-context deviations.
ST004 CandiSep 2022 - systemic / early antifungal use	Low	Low	Low	Low	Low	Low	Central randomisation, assignment-based analysis and objective outcome ascertainment were preserved. Imperfect execution estimates real pathway delivery and was not treated as bias from trial-context deviations.

Study / result	D1	D2	D3	D4	D5	Overall	Support for judgment
ST005 Erb 2023 - 28-day mortality	Low	Low	Low	Low	Low	Low	Random-number allocation with sequential sealed envelopes, complete analysis of all 41 randomised participants, prospectively specified outcomes, and objective mortality support low risk. The small sample and planned futility stopping affect precision, not risk of bias.
ST005 Erb 2023 - DDD through day 28	Low	Low	Low	Low	Low	Low	The prespecified intervention allowed clinician veto; this was part of the intended strategy. DDD was objectively recorded for all randomised participants.
ST005 Erb 2023 - proven *Candida* infection to day 28	Low	Low	Low	Some concerns	Low	Some concerns	Culture-confirmed events were objective, but the open-label stopping decision could influence diagnostic intensity after discontinuation; events were also very sparse. Sparsity itself is addressed in GRADE.
ST019 Verma 2025 ACLF - 28-day survival	Some concerns	Some concerns	Low	Low	Low	Some concerns	The registry confirms parallel randomisation and blinded endpoint adjudication, but the available report package does not describe sequence generation or allocation concealment and provides limited information on strategy deviations. The primary outcome and time point were prospectively registered and all 216 randomised participants were represented in the survival analysis.
ST019 Verma 2025 ACLF - antifungal receipt / timing	Some concerns	Some concerns	Low	Low	Low	Some concerns	Randomisation/concealment reporting is incomplete, and adherence/deviation reporting is limited. Antifungal initiation and timing were objectively recorded for the randomised groups.
ST019 Verma 2025 ACLF - treatment success among treated patients	Some concerns	High	Low	Low	Low	High	The analysis excluded randomised participants who did not initiate antifungal therapy and conditioned on a post-randomisation variable; treatment success also required survival beyond 7 days. This cannot estimate the effect of assignment without substantial selection bias. The result was not used as a randomised treatment-effect estimate.

Panel C. ROBINS-I (2016) judgments for comparative non-randomised intervention-effect results

Study / target result	Confounding	Selection	Classification	Deviations	Missing data	Measurement	Reported result	Overall	Support for judgment
ST013 Gill 2019 - negative-T2MR era versus negative-BDG era; anidulafungin duration	Serious	Serious	Low	Serious	Moderate	Low	Moderate	Serious	Historical allocation, secular practice changes, baseline severity differences, and clinician-directed discontinuation leave important residual confounding and selection bias despite adjusted analyses. Days of therapy were objectively recorded.

Study / target result	Confounding	Selection	Classification	Deviations	Missing data	Measurement	Reported result	Overall	Support for judgment
ST017 Salhotra 2025 - BDG-negative treated versus untreated; ICU survival	Critical	Serious	Moderate	Serious	Moderate	Low	Critical	Critical	Treatment was strongly confounded by clinical indication and illness severity. The reported propensity-score analysis is internally inconsistent with the stated group sizes (31 matched treated patients despite 22 treated participants), so the adjusted estimate could not be reproduced and was excluded from causal interpretation.
ST022 Abi Kheir 2026 - algorithm period versus historical standard care; micafungin duration	Serious	Serious	Moderate	Serious	Moderate	Low	Moderate	Serious	Historical allocation permits secular changes in case mix, prescribing and laboratory workflow. The algorithm group contained numerically more high-risk, surgical and immunocompromised patients; selection from electronic reports and differential eligibility assessment may have altered enrolment. Micafungin duration was objective, but implementation fidelity and result timing varied. The overall pre-post estimate is at serious risk of bias; analyses restricted to timely or compliant cases select post-baseline subgroups and are not causal.

Target trial for Gill: eligible culture-negative ICU adults receiving empirical anidulafungin would be assigned at the same clinical decision point to a negative-T2MR-supported or negative-BDG-supported discontinuation strategy.

Target trial for Salhotra: BDG-negative ICU adults would be assigned to continued antifungal therapy or withholding at the same decision point.

Target trial for Abi Kheir: eligible non-neutropenic ICU adults starting empirical micafungin would be assigned contemporaneously to a pharmacy-driven BDG pathway or standard care with identical laboratory access and follow-up.

None of the three observational studies approximated its target trial sufficiently for a low- or moderate-risk causal estimate.

Panel D. Structured appraisal of non-comparative, single-arm, audit, and management-impact reports

Study / report	Design / counterfactual	Selection / confounding	Fidelity, ascertainment, and reporting limitations	Permitted interpretation
ST009 Prattes 2014	Retrospective decision-impact cohort; no concurrent comparator	Testing and treatment selected by clinical suspicion; BDG contribution cannot be separated from concurrent information	Small cohort; management attribution and follow-up not standardised	Decision-impact feasibility only; no effectiveness or safety claim.

Study / report	Design / counterfactual	Selection / confounding	Fidelity, ascertainment, and reporting limitations	Permitted interpretation
ST011 Posteraro 2016	Retrospective observational management cohort; no protocol control	Treatment decisions reflected severity, pre-test probability, and clinician response	Cointerventions and diagnostic verification were not standardised; selective outcome availability possible	Narrative pathway evidence only; parent cohort for Giacobbe.
ST012 Nucci 2016	Prospective multicentre single-arm stopping cohort	Eligibility for stopping required repeatedly negative cultures/BDG, creating a selected low-risk subgroup	No concurrent comparator; small event counts; safety ascertainment depended on follow-up intensity	Feasibility and rule-out plausibility only; comparative safety not established.
ST014 Hare 2020	Prospective audit/service-improvement pathway	Episode selection, compliance, clinician override, and secular trends limit attribution	Episode-level analysis and small subgroups; no adjusted concurrent comparison	Implementation evidence only.
ST015 Helweg-Larsen 2021 - management outcomes	Prospective stewardship programme; no randomised or contemporaneous control	Testing and treatment/withholding remained clinician-directed; programme participants were highly selected	Historical consumption comparison and pathway-specific classifications; incomplete separation of test and clinical judgment	Feasibility and management association only; diagnostic estimates separately assessed with QUADAS-3.
ST016 Carbonell 2022	Prospective diagnostic-management cohort	Galactomannan testing was restricted to deteriorating patients; treatment followed disease classification	Small CAPA subgroup; severity-driven ascertainment and no comparable untreated pathway group	Pathway description and disease association only.
ST018 Zhao 2025 - management-impact/mortality claims	Retrospective paired-testing cohort; no intervention assignment	"Positive clinical impact" was retrospectively classified, and treatment changes were clinician-mediated and not antifungal-specific	Mortality contrasts lack an independent counterfactual and are vulnerable to immortal-time, indication, and post-result classification bias	Hypothesis generation only; no causal mortality benefit claim. Diagnostic accuracy separately assessed with QUADAS-3.
ST020 LAMBDA 2023	Prospective single-arm phase 2 biomarker-guided intervention	Selected high-risk postoperative cohort; no concurrent comparator	n=40; sparse infections/adverse events; all patients received day-1 therapy before the BDG-guided stop/continue rule	Feasibility, tolerability, and exposure description only; comparative effectiveness and definitive safety not established.
ST021 Albanell-Fernández 2026	Prospective single-centre biomarker-supported discontinuation cohort; no contemporaneous no-algorithm comparator	Groups were defined by BDG result and differed in pre-test risk; treatment decisions also used clinical course, microbiology and specialist judgment, so the effect of the pathway or assay cannot be isolated	Small cohort, only five *invasive-candidiasis* cases, result-dependent management, incomplete adherence, weekday/weekend turnaround differences and limited power for subsequent infection or mortality	Feasibility, implementation, false-negative and exposure association only; comparative effectiveness and fungal safety are not established.

Panel E1. QUADAS-3 synthesis questions and ideal test-accuracy trials

Synthesis question	Question	Ideal test-accuracy trial
Candida rule-out / rule-in	In critically ill adults undergoing diagnostic or empirical-treatment reassessment, what are the sensitivity and specificity of prespecified fungal-biomarker rules for invasive *Candida* infection or candidemia?	Prospective consecutive ICU cohort; index test interpreted at a prespecified threshold without knowledge of target-condition status; uniform independent reference standard excluding the index test; complete verification and clinically relevant follow-up.
Aspergillus molecular diagnosis	In immunocompromised critically ill adults with suspected IPA, what are the sensitivity and specificity of BALF mNGS at a prespecified positivity rule?	Prospective consecutive cohort; mNGS interpreted blinded at a prespecified threshold; independent EORTC/MSGERC-compatible target-condition adjudication not incorporating mNGS; complete paired verification.

Panel E2. QUADAS-3 estimate-level risk-of-bias and applicability judgments

Report / selected estimate	Participants	Index test	Target condition	Analysis	Overall RoB	Applicability: participants / index / target	Support for judgment
ST010 Martínez-Jiménez 2015 - serial combined CAGTA/BDG for proven+probable IC	High	Low	High	High	High	High / Low / High	Selected patients had already started empirical therapy and complete-case analysis excluded those with fewer than three samples. Biomarker thresholds and blinded index interpretation were prespecified, but the composite probable-IC target condition was partly subjective and affected by prior treatment; multiple serial models were explored.
ST015 Helweg-Larsen 2021 - combined T2MR/mannan classification of IC	Unclear	High	High	High	High	High / High / High	Consecutive enrolment was not clearly documented. T2MR/mannan results were incorporated directly into "likely/possible IC" definitions, and final classification was retrospective; this creates incorporation and review bias. The pathway-specific target condition is not directly transferable to independent accuracy assessment.
ST018 Zhao 2025 - BALF mNGS for IPA	High	Low	High	High	High	High / Low / High	Retrospective complete-case selection required simultaneous mNGS and conventional tests. A prespecified mNGS positivity rule was reported, but final diagnosis was made by clinicians using mNGS, conventional tests, imaging, and clinical data, so the target condition was not independent of the index test. ROC estimates are therefore circular.

Report / selected estimate	Participants	Index test	Target condition	Analysis	Overall RoB	Applicability: participants / index / target	Support for judgment
ST004-L Standl 2026 - manufacturer-cutoff antigen assays	Low	Low	Unclear	High	High	Low / Low / High	The multicentre parent cohort was prospectively assembled, and manufacturer thresholds were fixed. However, ICI classification was limited to 96 hours, 14 patients lacked the second sample, event counts-especially candidemia-were small, and the clinically relevant target-condition follow-up was shorter than the stewardship question.
ST004-L Standl 2026 -optimised cutoffs and assay combinations	Low	High	Unclear	High	High	Low / High / High	Cutoffs and combinations were selected from the same cohort using ROC, regression, and decision-tree procedures. Bootstrap internal validation does not remove optimism or multiplicity, and no external validation was available; candidemia estimates were based on 14 cases.
ST011 Posteraro 2016 - BDG ≥80 pg/mL for contemporaneous candidemia	High	Low	High	High	High	High / Low / High	Retrospective selection required sepsis, *Candida* score ≥3 and available BDG. Blood culture was an imperfect reference for invasive disease, verification and follow-up were not standardised, and the descriptive estimate was not prospectively designed as an accuracy study.
ST021 Albanell-Fernández 2026 - Wako BDG ≥7 pg/mL for *invasive candidiasis* in high-risk patients	High	Low	High	High	High	High / Low / High	Participants were selected after empirical echinocandin initiation and stratified by *Candida* score; only five invasive-candidiasis cases occurred. The threshold was prespecified, but clinical/microbiological follow-up and treatment influenced target-condition ascertainment, and sensitivity/specificity estimates were extremely unstable. Predictive values were strongly prevalence-dependent and partly adjusted to assumed incidence.
ST022 Abi Kheir 2026 - BDG classification for confirmed *invasive candidiasis*	High	Low	High	High	High	High / Low / High	The diagnostic analysis was embedded in a retrospective implementation cohort and contained only five confirmed cases; one patient lacked a classified BDG result. Verification was clinician-directed, treatment could affect culture yield, and diagnostic performance was not the primary study objective. The reported NPV and PPV are therefore highly uncertain and not transportable as treatment thresholds.

QUADAS-3 applicability categories are reported in the order participants / index test / target condition. “High” indicates a concern regarding direct applicability to the stated synthesis question, not necessarily poor conduct of the original study.

Panel F. Structured appraisal of the linked decision-analytic report

Report	Source data	Comparator	Key assumptions	Uncertainty / validation	Permitted use
ST011-L Giacobbe 2019	Post hoc re-analysis of 186/198 participants from the Posteraro cohort	Observed BDG-informed decisions versus a hypothetical universal empirical-treatment strategy	DOOR rankings, treatment-cost inputs, and assumptions about alternative treatment choices; no randomised counterfactual	Bootstrap confidence intervals were reported, but there was no external validation or prospective calibration	Decision-utility context only; not a clinical treatment-effect estimate and no additional independent population.

Panel G. Cross-tool interpretation safeguards

Safeguard	Rule applied
Risk of bias versus imprecision	Small sample size, sparse events, wide confidence intervals, and early stopping for futility were not used to inflate risk-of-bias judgments unless they produced a specific bias mechanism. These limitations are handled in GRADE and interpretation.
Open-label trials	Lack of blinding was not treated as automatic bias. D2 or D4 was raised only where clinician behaviour, treatment crossover, or diagnostic ascertainment could plausibly bias the specific result.
Diagnostic accuracy versus treatment effect	A high or low diagnostic-accuracy risk-of-bias judgment does not establish clinical benefit or safety. Diagnostic reports were used only for assay behaviour, thresholds, and pathway plausibility.
Critical ROBINS-I result	The Salhotra propensity-score survival estimate was excluded from causal interpretation. Descriptive cohort information can be retained if clearly separated from the invalid adjusted estimate.
ACLF randomised trial	The separate ACLF trial was not pooled with *Candida* RCTs. Its RoB 2 appraisal applies to its own randomised strategy contrast and does not imply compatibility with the *Candida*estimand.

Abbreviations. ACLF, acute-on-chronic liver failure; BDG, beta-(1→3)-D-glucan; CAGTA, *Candida albicans* germ-tube antibody; DDD, defined daily doses; IC/ICI, invasive *Candida* infection; IFI, invasive fungal infection; IPA, invasive pulmonary aspergillosis; mNGS, metagenomic next-generation sequencing; RCT, randomised controlled trial; RoB 2, Cochrane Risk of Bias 2; ROBINS-I, Risk Of Bias In Non-randomised Studies of Interventions; QUADAS-3, Quality Assessment of Diagnostic Accuracy Studies 3.

Addendum to Panel E. Study flow and analytic missingness for selected diagnostic estimates

Report	Study flow	Missingness / analytic implication
Martínez-Jiménez 2015	100 treated adults enrolled; 63 were in ICU.	Serial complete-case denominators varied because participants with fewer than three samples were excluded.
Posteraro 2016	201 eligible high-risk septic ICU patients identified; 3 excluded; 198 analysed.	Three were excluded because timing from first BDG result to treatment initiation could not be determined.
Helweg-Larsen 2021	219 non-neutropenic ICU patients entered the prospective programme.	Testing and final classification were pathway-dependent; paired-test availability and clinician-directed verification limited an independent accuracy estimand.
Zhao 2025	171 paired-testing complete cases from six ICUs (67 IPA; 104 non-IPA).	Retrospective inclusion required available BALF mNGS and conventional testing; the source population denominator was not reported.
Standl 2026	342 randomised in CandiSep; 3 withdrew; 339 analysed.	Fourteen early deaths had only the first serum sample; 664 sera were analysed.
Albanell-Fernández 2026	7471 ICU admissions; 124 patients with sepsis/risk factors received empirical antifungal therapy; 92 met entry criteria; 2 lacked analysable BDG; 90 were analysed.	The main exposure comparison excluded seven patients with confirmed fungal infection (83 analysed). Diagnostic performance was based on only five *invasive-candidiasis* cases; result-defined groups were non-comparable.
Abi Kheir 2026	318 encounters screened: 178 in the standard-care period and 140 in the algorithm period; 123 and 85 were excluded, leaving 55 patients per group.	Eligibility was reconstructed from electronic records in a historical comparison. In the algorithm group, 22 results were positive, 4 indeterminate and 28 negative; one patient was not represented in those categories, and diagnostic estimates were based on five confirmed cases.

TABLE S7. GRADE SUMMARY OF FINDINGS AND CERTAINTY ASSESSMENT

Certainty was assessed for randomised treatment-effect evidence within 21 included reports representing 19 independent studies. Because biomarker-guided discontinuation, biomarker-triggered initiation, and the mixed-fungal ACLF strategy address materially different decisions, they are presented as separate comparisons. A descriptive mortality aggregation across opposing rule directions is confined to Supplementary Table S8 and is not assigned a unitary GRADE rating.

Panel A. GRADE framework and prespecified decision rules

Domain	Operational rule
Starting certainty	Randomised evidence started at high certainty. Non-randomised, diagnostic-accuracy, single-arm, and decision-analytic evidence was not graded as randomised treatment-effect evidence.
Comparison structure	Separate Summary of Findings sections were used for: (1) *Candida* discontinuation/interruption/termination strategies; (2) *Candida* initiation/pre-emptive strategies; and (3) the mixed-fungal ACLF randomised strategy.
Risk of bias	Outcome-specific RoB 2 judgments from Supplementary Table S6 were considered at the body-of-evidence level. A trial-level “some concerns” judgment did not automatically trigger downgrading unless the affected result contributed materially to the evidence body.
Inconsistency	Direction, magnitude, clinical construct, and statistical heterogeneity were considered together. I^2=0% was not interpreted as proof of homogeneity when few studies were available. Single-study outcomes were not downgraded for inconsistency.
Indirectness	Downgrading was applied only when the population, intervention, comparator, or outcome did not directly answer the stated comparison. The ACLF trial was judged against its own liver-ICU question and not against the *Candida* stopping question.
Imprecision and thresholds	For binary clinical outcomes, appreciable benefit and harm were operationalised as RR 0.75 and RR 1.25. Approximate equal-allocation OIS used two-sided alpha 0.05 and 80% power. Confidence intervals, OIS, event counts, and estimator robustness were considered together. Modified HKSJ intervals were retained as conservative stress tests and were not used mechanically when tau^2=0 or k=2.
Publication bias / missing evidence	Formal small-study-effect testing was not performed because no synthesis included ten studies. No automatic downgrade was applied, but undetected missing evidence remains possible.
Language of interpretation	Moderate certainty: “probably”; low certainty: “may”; very-low certainty: “the evidence is very uncertain.” No estimate was interpreted as proving equivalence, non-inferiority, or safety.

Panel B1. *Candida* exposure-reduction strategies versus usual care

Outcome	Evidence	Strategy arm	Control arm	Relative / study-level effect	Absolute effect	Certainty	GRADE interpretation
28- or 30-day all-cause mortality	3 RCTs; 258 participants	34/126	38/132	DL RR 0.94 (95% CI 0.63-1.40); modified HKSJ RR 0.94 (0.39-2.26)	Control risk 288/1,000; 272/1,000 with the strategy (183-404); 16 fewer/1,000 (105 fewer to 116 more)	Low	Imprecision −2. Rouzé and Erb were low risk for mortality; De Pascale had some concerns but leave-one-out and full-denominator missing-outcome analyses preserved the conclusion. The CI crosses appreciable benefit and harm, and OIS is far from met. The strategy may have little or no effect on short-term mortality, but important benefit or harm remains possible.

Outcome	Evidence	Strategy arm	Control arm	Relative / study-level effect	Absolute effect	Certainty	GRADE interpretation
Invasive *Candida* events diagnosed after randomisation	3 RCTs; 258 participants	10/126	6/132	DL RR 1.85 (95% CI 0.51-6.65); REML/Wald RR 2.00 (0.72-5.50); modified HKSJ RR 2.00 (0.15-25.80)	Control risk 45/1,000; 84/1,000 (23-302); 39 more/1,000 (22 fewer to 257 more)	Very low	Risk of bias −1; indirectness −1; imprecision −2. Only 16 events diagnosed after randomisation were observed; the prespecified proven-or-probable Rouzé endpoint was used, baseline infections were excluded when reports separated them, and policy-level fungal safety is unresolved.
Reduction in systemic antifungal exposure	3 RCTs; 258 participants	Different constructs	Different constructs	Not pooled: Rouzé early stop 29/54 vs 1/55; De Pascale median duration 2 vs 10 days; Erb mean 11.3 vs 10.5 DDD	No common absolute effect was estimable	Low	Inconsistency −1; imprecision −1. Rouzé and De Pascale showed marked reductions, whereas the small, early-terminated Erb trial did not because the rule rarely became actionable. Endpoints were not combinable and the evidence base was small. Biomarker-supported stopping may reduce exposure when the gate opens, but magnitude and reproducibility are uncertain.

Panel B2. *Candida* initiation or pre-emptive strategies versus usual care

Outcome	Evidence	Strategy arm	Control arm	Relative effect	Absolute effect	Certainty	GRADE interpretation
28-day all-cause mortality (CandiSep)	1 RCT; 339 analysed participants	58/172	51/167	RR 1.10 (95% CI 0.81-1.51)	Control risk 305/1,000; 337/1,000 (247-460); 32 more/1,000 (58 fewer to 154 more)	Low	Imprecision −2. Mortality was objectively ascertained under assignment-based analysis, but this single trial was underpowered and the CI remained compatible with clinically important benefit and harm.
Receipt of systemic antifungal therapy	2 RCTs; 403 participants	124/219	51/184	DL RR 2.06 (95% CI 1.58-2.67); modified HKSJ RR 2.06 (0.38-11.21)	Control risk 277/1,000; 570/1,000 (439-740); 293 more/1,000 (162-463 more) using the DL interval	Moderate	Imprecision −1. Both trials showed increased treatment receipt and the conventional RR excludes no effect; only two trials contributed and OIS was not met. Initiation-oriented algorithms probably increase systemic antifungal treatment, although the magnitude is uncertain and this is not evidence of clinical benefit.

Hanson 2012 reported ICU-discharge survival as rounded percentages (85% versus 81%). Exact death counts were not reconstructed; the outcome is reported narratively and contributes to no quantitative mortality synthesis.

Panel B3. Mixed-fungal ACLF strategy: confirmation-dependent pre-emptive therapy versus immediate empirical therapy

Outcome	Evidence	Confirmation-dependent arm	Immediate empirical arm	Effect estimate	Absolute effect	Certainty	GRADE interpretation
28-day all-cause mortality	1 pragmatic RCT; 216 participants	94/108 deaths	70/108 deaths	RR 1.34 (95% CI 1.15-1.57); crude RD +22.2 percentage points (Newcombe 95% CI +10.9 to +32.9)	222 more deaths/1,000 (109-329 more)	Low	Risk of bias -1; imprecision -1. In this selected liver-ICU population, the confirmation-dependent strategy may increase mortality relative to immediate empirical treatment. The result does not isolate the effect of one biomarker.

The comparison is graded for adults with ACLF and suspected mixed invasive fungal infection. No indirectness downgrade was applied within that exact question. Limited reporting of randomisation/concealment and strategy deviations informed risk of bias; the confidence interval crossed the prespecified RR 1.25 threshold and the approximate OIS was narrowly unmet.

Panel C. Approximate optimal information size and imprecision audit

Outcome	Control risk	Thresholds	Approximate OIS: benefit / harm	Observed N	Interpretation
Exposure-reduction mortality	28.8%	RR 0.75 / 1.25	1,140 / 1,326	258	OIS not met; CI crosses both appreciable-benefit and appreciable-harm thresholds.
Post-randomisation invasive *Candida* safety	4.5%	RR 0.75 / 1.25	9,284 / 11,796	258	OIS profoundly unmet; very sparse events and extreme interval width.
CandiSep 28-day mortality	30.5%	RR 0.75 / 1.25	1,054 / 1,214	339	OIS not met; CI excludes RR 0.75 but includes RR 1.25.
Initiation/pre-emptive antifungal use	27.7%	RR 0.75 / 1.25	1,200 / 1,402	403	OIS not met; conventional and small-sample intervals give different impressions of precision.
ACLF 28-day mortality	64.8%	RR 0.75 / 1.25	292 / 234	216	OIS narrowly unmet for the harm threshold; CI excludes no effect but crosses RR 1.25.

OIS values are approximate equal-allocation sample sizes calculated from the observed control risk, two-sided alpha 0.05, 80% power, and RR thresholds 0.75 and 1.25. They support-but do not replace-judgment based on confidence intervals, event counts, clinical thresholds, and model robustness.

Panel D. Domain-level rationale by graded outcome

Outcome	Risk of bias	Inconsistency	Indirectness	Imprecision	Missing evidence	Final certainty
Exposure-reduction mortality	Not serious at body level: Rouzé and Erb were low risk for mortality; De Pascale had some concerns, but leave-one-out and full-denominator missing-outcome analyses preserved the conclusion that important benefit or harm remained possible.	Not serious: point estimates were compatible; few trials limit detection of heterogeneity.	Not serious: directly addresses stopping or shortening treatment.	Very serious: n=258; DL 0.63-1.40; OIS far from met; both appreciable benefit and harm remain possible. Modified HKSJ was a conservative stress test, not a mechanical downgrade rule.	Not assessable; no downgrade.	Low
Post-randomisation invasive *Candida* safety	Serious: open-label stopping could alter diagnostic intensity; De Pascale also had post-randomisation exclusions.	Not serious statistically; I^2=24.3%, but sparse data make heterogeneity estimates weak.	Serious: event definitions and observation windows differed.	Very serious: 16 events; DL 0.51-6.65; modified HKSJ 0.15-25.80; OIS profoundly unmet.	Not assessable; no downgrade.	Very low
Exposure reduction	Not serious at body level: outcome ascertainment was objective or explicitly defined; study-level concerns were not judged sufficient to downgrade the body of evidence for risk of bias.	Serious: marked reductions in two trials were not reproduced in Erb.	Not serious for the qualitative question of whether an explicit stopping rule can reduce exposure in eligible patients.	Serious: small total evidence base, non-combinable endpoints, and a null low-activation trial make the stability and magnitude of the effect uncertain.	Not assessable; no downgrade.	Low
CandiSep mortality	Not serious: all RoB 2 domains were judged low for the assignment-based mortality effect.	Not applicable: one study.	Not serious: direct initiation-strategy mortality outcome.	Very serious (−2): OIS was not met and the CI remained compatible with clinically important benefit and harm.	Not assessable; no downgrade.	Low
Initiation/pre-emptive antifungal use	Not serious at body level: assignment-based treatment receipt was objectively recorded; deviations would be expected to attenuate rather than create the consistent direction.	Not serious: both estimates favoured increased use.	Not serious: directly measures treatment receipt under initiation/pre-emptive rules.	Serious: only two trials and OIS not met. The conventional interval establishes direction, while the conservative k=2 HKSJ stress test shows uncertainty in magnitude.	Not assessable; no downgrade.	Moderate
ACLF mortality	Serious: sequence generation/concealment and deviations were incompletely reported.	Not applicable: one study.	Not serious for the ACLF liver-ICU question.	Serious: OIS narrowly unmet and CI crosses the RR 1.25 threshold.	Not assessable; no downgrade.	Low

Panel E. Randomised analyses not assigned a separate certainty rating

Analysis	Result / evidence	Reason not separately graded
Descriptive 28/30-day mortality aggregation	Four RCTs; RR 1.04 (95% CI 0.81-1.33); modified HKSJ 0.70-1.55.	Combines opposing rule directions and is retained only in Supplementary Table S8; no unitary intervention effect or certainty rating.
Rule-out mortality missing-outcome robustness	Leave-one-out without De Pascale RR 0.88 (95% CI 0.52-1.49); all-missing-alive RR 0.93 (0.62-1.39); known day-0 death restored RR 0.96 (0.64-1.42); best case RR 0.82 (0.56-1.20); worst case RR 1.09 (0.70-1.71).	Robustness analyses restored the randomised denominators under explicit scenarios; none resolved clinically important benefit or harm. Full calculations are in Supplementary Table S8.

Analysis	Result / evidence	Reason not separately graded
CandiSep treatment within 96 hours	RR 8.16 (95% CI 4.39-15.16).	Single-study mechanistic exposure endpoint, subsumed under systemic treatment receipt.
ACLF treatment success among treated participants	40/107 versus 15/89 among those who initiated therapy.	Conditions on post-randomisation treatment and survival; high risk of selection bias for an assignment-effect interpretation.
LAMBDA outcomes	Prospective single-arm phase 2 pathway; 26/40 discontinued after negative BDG; 30-day mortality 6/40.	No concurrent comparator; feasibility/tolerability evidence only.

Abbreviations. ACLF, acute-on-chronic liver failure; BDG, beta-(1→3)-D-glucan; CI, confidence interval; DDD, defined daily doses; DL, DerSimonian-Laird; GRADE, Grading of Recommendations Assessment, Development and Evaluation; HKSJ, modified Hartung-Knapp small-sample inference; OIS, optimal information size; RCT, randomised controlled trial; RD, risk difference; RR, risk ratio.

Certainty ratings apply to the specified outcome, population, comparison, and decision rule. They do not apply to fungal biomarkers as a unitary intervention and do not establish equivalence, non-inferiority, or mycological safety.

TABLE S8. RANDOMISED EVENT COUNTS, STUDY-LEVEL EFFECTS AND SENSITIVITY ANALYSES

This table reports randomised event counts, study-level effects, pooled estimates, estimator and sparse-data sensitivities, the separate ACLF randomised comparison, and the unpooled LAMBDA single-arm pathway. Observational and diagnostic reports did not contribute to quantitative pooling.

Panel A. Pooled randomised analyses and sensitivity estimates

Analysis and source PMIDs	Studies	Biomarker-guided	Control	Effect estimate	Heterogeneity	Role in manuscript
Descriptive 28/30-day mortality aggregation PMIDs: 28936678; 32891170; 35708758; 37823695	k=4	92/298	89/299	RR 1.04 (95% CI 0.81-1.33); *P*=0.75	I^2=0%; τ^2=0	Supplement-only descriptive aggregation across opposing rule directions; no unitary effect or GRADE rating.
Exposure-reduction mortality PMIDs: 28936678; 32891170; 37823695	k=3	34/126	38/132	RR 0.94 (95% CI 0.63-1.40); *P*=0.78	I^2=0%; τ^2=0	Decision-specific rule-out mortality analysis; Figure 2(b); low certainty owing to very serious imprecision.
CandiSep 28-day mortality PMID: 35708758	k=1	58/172	51/167	Crude RR 1.10 (95% CI 0.81-1.51)	Not applicable	Separate rule-in mortality estimate; reported in main text/Table 2 rather than a single-study forest panel; low certainty.
Post-randomisation invasive *Candida* events under rule-out assignment PMIDs: 28936678; 32891170; 37823695	k=3	10/126	6/132	RR 1.85 (95% CI 0.51-6.65); *P*=0.35	I^2=24.3%; τ^2=0.348	Intention-to-treat policy-safety analysis using proven-or-probable Rouzé events; Figure 2(c); 16 events; very low certainty.
Systemic antifungal receipt in rule-in trials PMIDs: 22879929; 35708758	k=2	124/219	51/184	RR 2.06 (95% CI 1.58-2.67); *P*<0.001	I^2=0%; τ^2=0	Exposure-direction pool; Figure 2(a); moderate certainty for increased treatment receipt.
Early antifungal treatment within 96 h (CandiSep) PMID: 35708758	k=1	84/172	10/167	RR 8.16 (95% CI 4.39-15.16); *P*<0.001	Not applicable	Single-study illustrative estimate; not pooled or separately GRADE-rated.

Panel B. Study-level event counts and effects used in pooled and sensitivity analyses

Panel B1. Mortality and fungal-safety analyses

Analysis	Study / PMID	Algorithmic intent	Biomarker	Biomarker-guided	Control	Study-level effect	Analysis set
Mortality-rule-out	Rouzé 2017; PMID 28936678	discontinuation/interruption	multimarker	15/54	15/55	RR 1.02 (95% CI 0.55-1.87)	110 randomised; 109 analysed
Mortality-rule-out	De Pascale 2020; PMID 32891170	discontinuation/interruption	BDG alone	15/53	15/55	RR 1.04 (95% CI 0.56-1.91)	120 randomised; 108 available-case
Mortality-rule-out	Erb 2023; PMID 37823695	termination	BDG + mannan	4/19	8/22	RR 0.58 (95% CI 0.21-1.62)	41 randomised/ analysed
Mortality-rule-in	CandiSep 2022; PMID 35708758	initiation/pre-emptive	BDG alone	58/172	51/167	RR 1.10 (95% CI 0.81-1.51)	342 randomised; 339 analysed
Post-randomisation invasive *Candida* events-rule-out	Rouzé 2017; PMID 28936678	discontinuation/interruption	multimarker	4/54	1/55	RR 4.07 (95% CI 0.47-35.29)	110 randomised; 109 analysed; prespecified proven-or-probable endpoint
Post-randomisation invasive *Candida* events-rule-out	De Pascale 2020; PMID 32891170	discontinuation/interruption	BDG alone	0/53	2/55	RR 0.21 (95% CI 0.01-4.22)	120 randomised; 108 available-case
Post-randomisation invasive *Candida* events-rule-out	Erb 2023; PMID 37823695	termination	BDG + mannan	6/19	3/22	RR 2.32 (95% CI 0.67-8.02)	41 randomised/ analysed

Event-definition note. The policy-safety pool used invasive *Candida* events first diagnosed after randomisation or explicitly described as subsequent/follow-up. For Rouzé, the prespecified proven-or-probable endpoint was primary (4/54 versus 1/55); the proven-only counts (2/54 versus 1/55) were retained as a labelled sensitivity. In De Pascale, 11 infections present at enrolment (6/53 versus 5/55) were excluded; the two follow-up infections (0/53 versus 2/55) entered the pool. This definition avoids counting prevalent disease as a stopping-policy safety event.

Panel B2. Antifungal-use analyses

Analysis	Study / PMID	Algorithmic intent	Biomarker	Biomarker-guided	Control	Study-level effect	Analysis set
Systemic antifungal receipt	Hanson 2012; PMID 22879929	pre-emptive	BDG alone	25/47	5/17	RR 1.81 (95% CI 0.83-3.96)	64 randomised/analysed
Systemic antifungal receipt	CandiSep 2022; PMID 35708758	initiation/pre-emptive	BDG alone	99/172	46/167	RR 2.09 (95% CI 1.58-2.76)	342 randomised; 339 analysed
Treatment within 96 h	CandiSep 2022; PMID 35708758	initiation/pre-emptive	BDG alone	84/172	10/167	RR 8.16 (95% CI 4.39-15.16)	342 randomised; 339 analysed

Panel C. Randomised exposure and secondary endpoints not pooled as a single continuous effect

Study / PMID	Endpoint family	Strategy arm / pathway A	Comparator / pathway B	Quantitative handling	Reason for non-pooling / separation
Hanson 2012 PMID 22879929	Systemic antifungal treatment received; anidulafungin duration among treated	25/47 received systemic antifungals; treated patients median 13 days (range 2-27)	5/17 received systemic antifungals	Included only in the rule-in systemic-antifungal-receipt pool; ICU-discharge survival 85% versus 81% reported narratively.	Pre-emptive initiation algorithm; exact mortality counts were not reported and were not reconstructed.
Rouzé 2017 PMID 28936678	Early discontinuation before day 7; antifungal duration	29/54 stopped before day 7; median duration 6 days (IQR 4-13)	1/55 stopped before day 7; median duration 13 days (IQR 12-14)	Narrative exposure endpoint; early-stop binary endpoint not pooled with duration/DDD outcomes.	Construct differs from De Pascale duration and Erb DDD.

Study / PMID	Endpoint family	Strategy arm / pathway A	Comparator / pathway B	Quantitative handling	Reason for non-pooling / separation
De Pascale 2020 PMID 32891170	Antifungal duration during first 30 days; stopping rates over time	Median duration 2 days (IQR 1-3)	Median duration 10 days (IQR 6-13)	Narrative exposure endpoint; not converted to mean difference or SMD.	Median/IQR duration and time-specific stop rates not construct-equivalent to other trials.
Erb 2023 PMID 37823695	Antifungal consumption until day 28; therapy stopped by algorithm	Mean 11.3 DDD (SD 7.4); therapy stopped in 2/19	Mean 10.5 DDD (SD 6.3); 7/22 would have met stopping criteria by biomarkers	Narrative exposure endpoint; not pooled with duration outcomes.	Small early-terminated trial; DDD endpoint did not show exposure reduction; algorithm activation was low.
CandiSep 2022 PMID 35708758	Antifungal therapy during trial; early therapy within 96 h; antifungal-free survival	99/172 received systemic antifungals; 84/172 within 96 h; antifungal-free survival at day 28: 52/172	46/167 received systemic antifungals; 10/167 within 96 h; antifungal-free survival at day 28: 87/167	Antifungal-use binary endpoints analysed separately for initiation/pre-emptive exposure; not pooled with strict-core exposure reduction.	Biomarker positivity triggered treatment initiation rather than discontinuation.
Verma / ACLF 2025 PMID 41268913	Separate mixed-fungal randomised strategy: 28-day survival, antifungal initiation, treatment success	Immediate empirical: 38/108 alive (70/108 deaths); antifungals 107/108; treatment success 40/107	Confirmation-dependent pre-emptive: 14/108 alive (94/108 deaths); antifungals 89/108; treatment success 15/89	Separate from *Candida* pooling. Death RR for confirmation-dependent versus empirical 1.34 (95% CI 1.15-1.57); crude RD +22.2 percentage points (Newcombe 95% CI +10.9 to +32.9). Reported mortality HR for empirical versus pre-emptive 0.64 (0.47-0.88).	ACLF/liver-ICU population, mixed-fungal target, high pre-test risk, and bundled empirical-versus-confirmation-dependent pathway were incompatible with *Candida* trial estimands.
Rinaldi / LAMBDA 2023 PMID 37838147	Single-arm phase 2 BDG-guided L-AmB pathway after major intra-abdominal surgery	All 40 received L-AmB 5 mg/kg on day 1; 26/40 stopped on day 3 after baseline BDG <80 pg/mL; 30-day deaths 6/40	No concurrent comparator	Narrative feasibility/tolerability only; no comparative effect estimate	Single-arm design; cannot establish comparative efficacy, mortality benefit, or safety

Panel D. Estimator, small-sample, common-effect, and risk-difference sensitivity analyses

Analysis	k	DerSimonian-Laird RR (95% CI)	REML / modified HKSJ sensitivity RR (95% CI)	Mantel-Haenszel RR (95% CI)	Absolute-effect or missing-outcome sensitivity	Interpretation

Analysis	k	DerSimonian-Laird RR (95% CI)	REML / modified HKSJ sensitivity RR (95% CI)	Mantel-Haenszel RR (95% CI)	Absolute-effect or missing-outcome sensitivity	Interpretation
Descriptive 28/30-day mortality aggregation	4	1.04 (0.81-1.33)	REML/Wald 1.04 (0.81-1.33); mHKSJ 1.04 (0.70-1.55)	1.03 (0.81-1.32)	+12 deaths/1000 (−55 to +98)	Supplement-only aggregation across opposing rule directions; no unitary causal interpretation.
Exposure-reduction mortality	3	0.94 (0.63-1.40)	REML/Wald 0.94 (0.63-1.40); mHKSJ 0.94 (0.39-2.26)	0.94 (0.63-1.39)	−16 deaths/1000 (−105 to +116)	Insufficient precision to establish or exclude mortality harm.
Exposure-reduction mortality excluding De Pascale	2	0.88 (0.52-1.49)	τ^2=0; direction unchanged	Not separately used	Leave-one-out analysis	The central conclusion did not depend on De Pascale.
Exposure-reduction mortality: De Pascale missing-outcome scenarios	3	All missing alive 0.93 (0.62-1.39); known day-0 death restored 0.96 (0.64-1.42)	Best case 0.82 (0.56-1.20); worst case 1.09 (0.70-1.71)	Not separately used	Restored 60/60 randomised denominators; worst-case I^2=22.7%	No scenario resolved important benefit or harm.
Post-randomisation invasive *Candida* events under rule-out assignment	3	1.85 (0.51-6.65)	REML/Wald 2.00 (0.72-5.50); mHKSJ 2.00 (0.15-25.80)	1.83 (0.71-4.71)	DL RD +2.4% (−6.8% to +11.7%); MH RD +3.7% (−1.9% to +9.3%)	Sixteen events; no policy-level fungal-safety margin established.
Post-randomisation invasive *Candida* events: Rouzé proven-only sensitivity	3	1.65 (0.54-4.98)	REML/Wald 1.70 (0.61-4.78); mHKSJ 1.70 (0.16-17.72)	1.47 (0.55-3.93)	8/126 versus 6/132; I^2=6.2%; τ^2=0.078	Uses 2/54 versus 1/55 for Rouzé and reproduces the prior estimate. This sensitivity does not replace the prespecified proven-or-probable endpoint.
Systemic antifungal receipt in rule-in trials	2	2.06 (1.58-2.67)	REML/Wald 2.06 (1.58-2.67); mHKSJ 2.06 (0.38-11.21)	2.05 (1.58-2.67)	+293 treated/1000 (+162 to +463)	Direction consistent; magnitude and transportability remain uncertain.
Exposure-reduction mortality excluding low-activation Erb (post hoc)	2	1.03 (0.67-1.58)	τ^2=0; I^2=0%	Not separately used	Rouzé + De Pascale only	Conclusion unchanged; benefit and harm remain compatible.
Post-randomisation invasive Candida events excluding low-activation Erb (post hoc)	2	1.12 (0.06-20.13)	τ^2=2.645; I^2=59.7%	Not separately used	Rouzé + De Pascale only	Sensitivity addresses gate-activation concern; fungal-safety uncertainty widens markedly and remains unresolved.

Sparse-event, small-sample, and missing-outcome note. Risk-difference sensitivity used uncorrected arm risks. Modified HKSJ intervals were retained as conservative stress tests; with $\tau^2=0$, REML/Wald was the principal random-effects sensitivity. De Pascale sensitivity restored the 60/60 randomised denominators under explicit scenarios and included a leave-one-out analysis. Hanson ICU-discharge percentages were not converted into event counts. No model can establish policy-level fungal safety with 16 events.
